# Local inflammation at the salmon louse (*Lepeophtheirus salmonis*) attachment site contributes to copepodid rejection in coho salmon (*Oncorhynchus kisutch*)

**DOI:** 10.1007/s00441-025-03976-0

**Published:** 2025-06-04

**Authors:** Lene Sveen, Mark D. Fast, Torstein Tengs, Rachel A. Kline, Judit Aguilar Marti, Dominic Kurian, Gerrit Timmerhaus, Marianne Vaadal, Ross D. Houston, James E. Bron, Sean J. Monaghan, Haitham H. Mohammed, Rose Ruiz Daniels, Sarah Salisbury, Diego Robledo, Mark Braceland, Miroslava Hansen, Nicholas Robinson

**Affiliations:** 1 Nofima Muninbakken 9-13, Langnes, NO-9291 , Tromsø, Breivika Box 6122 Norway; 2 Hoplite Research Lab, Department of Pathology and Microbiology, AVC-UPEI, Charlottetown, PE Canada; 3 Benchmark Genetics, 1 Pioneer Building, Edinburgh Technopole, Penicuik, Milton Bridge UK; 4 Institute of Aquaculture, University of Stirling, Stirling, UK; 5 Onda (Formerly Centre for Aquaculture Technologies Canada – CATC), Prince Edward Island, Souris Canada; 6 Department of Rangeland, Wildlife and Fisheries Management, Texas A&M University, College Station, TX 77843 USA; 7 The Roslin Institute and Royal (Dick) School of Veterinary Studies, University of Edinburgh, Edinburgh, UK; 8 WellFish Tech Ltd. F117, University of the West of Scotland, High St., Paisley, PA1 2BE UK; 9 Norwegian Veterinary Institute, Elizabeth Stephansens Vei 1, Ås, 1433 Norway; 10 Deakin University, Victoria, 3225 Australia

**Keywords:** *L. salmonis*, Atlantic salmon, Chum salmon, Coho salmon, Pink salmon

## Abstract

The study investigates the susceptibility of Atlantic salmon (*Salmo salar*) and Pacific salmon species (pink salmon, *Oncorhynchus gorbuscha*; coho salmon, *Oncorhynchus kisutch*; and chum salmon, *Oncorhynchus keta*) to the parasitic salmon lice (*Lepeophtheirus salmonis*). The research had two main objectives: to characterize the morphology of the scaly skin in four salmonid species and to compare the cellular response at the louse attachment site in coho salmon and Atlantic salmon. Three consecutive challenge trials were conducted, with successful louse infestation only achieved across all four species in the third trial using mild anesthesia with tricaine methanesulfonate. Skin and fin samples were collected at 12, 24, 36, 48, 60, and 168 h post-infestation (hpi) for histological, proteomic, and spatial transcriptomic analyses. Results showed that chum salmon had significantly higher mucous cell coverage (30–40%) in the epithelium of scaly skin compared to Atlantic salmon (10%). At the louse attachment site in coho salmon, there was a greater influx of inflammatory cells at 36–48 hpi compared to Atlantic salmon. Proteomic analysis at 12 hpi and 36 hpi in coho salmon showed upregulation of neutrophil degranulation and formyl-methionyl-leucyl-phenylalanine signaling. Additionally, spatial transcriptomics at the attachment site showed local upregulation of inflammatory gene markers. These findings suggest that coho salmon mount a rapid and large-scale inflammatory response driven by neutrophils to louse attachment within the first 48 hpi. Overall, the study emphasizes the significance of local changes at the host-parasite interface for resistance mechanisms against salmon lice.

**Supplementary Information:**

The online version contains supplementary material available at 10.1007/s00441-025-03976-0.

## Introduction

Atlantic salmon (*Salmo salar*) are one of the most valuable finfish in aquaculture, but their sustainable production is hampered by both salmon louse (*Lepeophtheirus salmonis*) parasitism and the treatments used to remove lice (Fast 2014; Sommerset et al. 2023). *L. salmonis* exhibits a moderate level of host specificity, primarily infesting fish belonging to the *Salmo* and *Oncorhynchus* genera (Pike & Wadsworth 1999). Variations in susceptibility to *L. salmonis* infestations have been noted among salmonid species (Johnson & Albright 1992a; Pike & Wadsworth 1999) with Pacific species such as coho salmon (*Oncorhynchus kisutch*), pink salmon (*Oncorhynchus gorbuscha*), and chum salmon (*Oncorhynchus keta*) regarded as more resistant compared to the highly susceptible Atlantic salmon (Jones et al. 2007; Sutherland et al. 2014). However, there is ongoing debate in the literature regarding which salmonid species is most resistant to salmon louse infestation. Experimental trials with *L. salmonis* have shown that chum salmon are more susceptible to lice compared to pink salmon (Jones et al. 2007) and Atlantic salmon (Sutherland et al. 2014). In contrast, another study found that wild pink salmon exhibited higher lice prevalence compared to chum salmon (Nagasawa 2001). Previous experimental challenge trials with *L. salmonis* infestation in pink salmon and chum salmon have shown that sedation with anesthesia is necessary to successfully infest the fish (Jones et al. 2007; Sutherland et al. 2014). Anesthesia suppresses natural avoidance behaviors such as flashing, jumping, and rapid swimming, which are normal responses for pathogen evasion in salmonids (Bui et al. 2017).

In contrast, Atlantic salmon and coho salmon are generally not sedated in experimental infestation models with *L. salmonis*. While lice attach to both species, coho salmon reject the salmon lice within days, whereas infestations persist in Atlantic salmon (Fast et al. 2002b; Johnson & Albright 1992a). Multiple hypotheses exist regarding how coho salmon exhibit resistance to *L. salmonis*. One proposed mechanism involves hyperplasia of epithelial cells, particularly keratinocytes, along with the encapsulation of the lice (Braden et al. 2023; Johnson & Albright 1992a; Salisbury et al. 2024). This hyperplasia can be quite pronounced, sometimes visible to the naked eye, and is always localized to the attachment site of the lice. Additionally, coho salmon possess sacciform cells in the epithelial layer of their skin (Johnson & Albright 1992a), which are absent in Atlantic salmon. Sacciform cells have been observed throughout louse infestation in coho salmon skin (Braden et al. 2023), but it remains uncertain whether their numbers increase during infection or if they are consistently present. Lastly, histological analysis of the louse attachment site in coho salmon has revealed an influx of inflammatory cells into dermal tissue 1 day post-infestation (Johnson & Albright 1992b). Recently, single-nuclei RNA sequencing (snRNAseq) suggested that macrophages may play a key role in the coho salmon’s immune response to *L. salmonis* infestation (Salisbury et al. 2024). Due to the low number of neutrophils detected in this snRNAseq study, it was not possible to assess the transcriptomic response of this cell type in response to *L. salmonis* infestation (Salisbury et al. 2024). However, bulk transcriptomic analysis suggests that neutrophils play an important role in coho salmon’s response to *L. salmonis*, which is supported by elevated expression of the gene *C-type lectin domain family 4 member E* (*clec4e*) in infested individuals (Braden et al. 2023). *Clec4e* is an immune receptor involved in recognizing damage-associated molecular patterns and pathogen-associated molecular patterns. Since *clec4e* has recently been identified as a marker for neutrophils in coho salmon (Salisbury et al. 2024), neutrophils may play a potential role in detecting tissue damage at the attachment site, potentially influencing the host’s local immune response to lice.

Atlantic salmon are highly susceptible to *L. salmonis*. Unlike coho salmon, Atlantic salmon lack sacciform cells in the epithelium and do not exhibit extensive hyperplasia at the louse attachment site (Johnson & Albright 1992a). However, it is clear that Atlantic salmon initiate a rapid and large-scale cellular immune response in the skin when infested with lice (Krasnov et al. 2012; Skugor et al. 2008; Tadiso et al. 2011). In Atlantic salmon, the T-cell response is thought to dominate in lice-infested skin, with an increase in T-cell markers and the number of T-cells upon infestation (Braden et al. 2015; Fast et al. 2006; Salisbury et al. 2024; Skugor et al. 2008). Despite this rapid and large-scale immune response, the salmon louse is not rejected in Atlantic salmon. Transcriptomic analysis of lice-infested skin suggests that *L. salmonis* suppresses the immune response in Atlantic salmon by downregulating key host immune pathways, including those involved in inflammation and wound healing (Braden et al. 2020; Tully & Nolan 2002; Umasuthan et al. 2020). Furthermore, recent studies suggest that the localized transcriptomic response at the specific point of louse attachment differs from the more general response in the skin further away from the attachment site (Øvergård et al. 2023; Sveen et al. 2021a; Umasuthan et al. 2020). Additionally, the transcriptomic response in the upper layer of the skin (epithelium and scales) differs from that in the deeper dermal tissue during lice infestation in response to salmon lice infestation (Sveen et al. 2021a), suggesting that different cell types in the skin respond in different ways to lice infection. As transcriptome analysis only measures the number of mRNA molecules, downregulation of genes may indirectly suggest a change in cell populations, such as fewer inflammatory cells at the site of attachment. Considering this, labial gland proteins from the salmon louse have received attention due to their immunosuppressive role (Midtbø et al. 2024; Øvergård et al. 2022). The salmon louse has labial gland ducts that extend into the oral cavity of the louse (Øvergård et al. 2022), and the labial gland proteins may be of particular importance for host settlement, as they have been found to dampen immune cell activity (Midtbø et al. 2024). If the lice manage to dampen the immune response at the narrow interface between the host and the parasite, they could, in principle, feed on host tissue undisturbed.

The attachment site of the parasite is the specific location on a host’s body where the ectoparasite attaches and resides to feed (Gonzalez-Alanis et al. 2001; Kabata 1974; Pike et al. 1993). Infestation with *L. salmonis* begins with the infectious copepodid stage (Hamre et al. 2013; Johnson & Albright 1991), which attaches to the epithelium of the fish’s fins, body, or gills using specialized appendages, such as antennae and maxillipeds, which have hooks that help the parasite grip onto the host tissue. After the initial attachment, the copepodids molt into the chalimus stage. At this point, the parasite is sessile, attaching itself to the host using a frontal filament (Bron 1993; Gonzalez-Alanis et al. 2001). In terms of anatomy, the mouth tube is located relatively close to the frontal filament, but they each serve different functions. The frontal filament is used primarily for attachment, anchoring the louse to the host, while the mouth tube is designed specifically for feeding (Kabata 1974). Hence, it has previously been suggested that the anchoring of the louse at this stage using the frontal filament may be a weak point in the development cycle of *L. salmonis* where the lice is most vulnerable (Gonzalez-Alanis et al. 2001); however, it has been unclear whether more resistant salmonid species like coho salmon are able to target this proposed weak point in the life cycle by weakening the attachment by the parasite to the host.

The current study had two aims. First, we aimed to characterize the morphology of the scaly skin of the body in four salmonid species, focusing on both infested and control fish, but not at the exact point of louse attachment. Second, we aimed to compare the cellular response at the direct point of attachment. Due to difficulties in establishing infestation in the Pacific salmonid species, a total of three consecutive challenge trials were conducted on the same group of fish. The first two trials were performed on unsedated fish challenged with *L. salmonis*, but these attempts did not result in any lice attaching to pink salmon and chum salmon. Hence, a third challenge trial was conducted on sedated fish, using the same group as in the previous two attempts. The third trial was successful, and tissue samples were collected from infested fish at four time points within the first 2.5 days post-infestation (dpi): 12, 24, 36, and 48 h post-infestation (hpi), followed by a sampling at 60 hpi and a final sampling at 168 hpi. Uninfested control samples were collected from all four species at 36 and 168 hpi. Skin samples collected from the body flanks were examined in all species, focusing on quantitative differences in epithelial area, mucous cell number, and mucous cell area across all four salmonid species. Furthermore, this study undertook molecular and histological analyses of the lice attachment site in coho salmon and Atlantic salmon to better characterize the local responses at the lice attachment site.

## Material and methods

### Source of fish and animal husbandry

Coho salmon and pink salmon were provided by Quinsam River Hatchery, Nanaimo, British Columbia, and transferred to Level III Quarantine Facility (CATC), Prince Edward Island, Canada (March 2021), at an estimated weight for coho salmon of 1–2 g, and an estimated weight of 0.25–0.3 g for pink salmon at shipment. The chum salmon were transferred at an estimated weight of 0.6 g from Inch Creek Hatchery, Dewdney, British Columbia, to the same Level III Quarantine Facility (CATC), Prince Edward Island, Canada (March 2021). Shipment of the salmon was done according to industry standard and involved packaging of the fish in chilled coolers containing freshwater from the source, supersaturated with oxygen (~250%), and a transport density of 75–100 g in shipping boxes (30 × 15 × 16 inch) with overnight transportation. Total transit time did not exceed 36 h. For Atlantic salmon, two thousand eggs from 10 families were donated to the project by StofnFiskur HF (Benchmark, Iceland) and synchronized to 360°d at delivery and shipped chilled on ice to The Center for Aquaculture Technologies Souris Canada (CATC) in late November 2020.

All four fish species were acclimated to ambient recirculating conditions (freshwater, 10–12 °C) over several hours in 150-L tanks. All four species were maintained at 12-h light:12-h dark cycles, flow rate of 2–4 L/min, and 90–101% dissolved oxygen. When the population average weight reached 15 g, along with visual indicators of smoltification such as loss of parr marks, Instant Ocean® (Blacksburg, USA) was slowly added to the partially recirculating system such that salinity was increased by four to five ppt per day to achieve a salinity of 32–33 ppt over a time period of 7 days. The fish were fed to satiation 2–3 times daily with a commercially available diet (Skretting; Skretting, Crum initially, and later 2.3 Nutra RC).

### Three experimental challenge attempts with *L. salmonis*

The challenge experiments were designed to collect samples from lice-infested fish, aiming to compare skin and molecular responses among four salmonid species with varying levels of susceptibility to *Lepeophtheirus salmonis*. In total, 600 naïve fish, 150 of each salmonid species, Atlantic salmon, coho salmon, pink salmon, and chum salmon, were incorporated into the lice challenge trials. The fish were transferred from the rearing unit at CATC to the lice challenge unit CATC. On the day of distribution to the study tanks, the bulk weights of a minimum of 50 individuals per species were recorded. Average sizes (± std) for the four species were as follows: Atlantic salmon 40.0 ± 8.98 g; coho salmon 12.5 ± 2.85 g; pink salmon 50.1 ± 19.34 g; chum salmon 80.6 ± 21.1 g. The study consisted of eight study groups, as outlined in Table 1. Fish challenged with lice were kept in triplicate tanks, whereas duplicated tanks were used for control fish. All fish were in good health prior to being enrolled in the study.

**Table 1:** Number of study groups, number of fish tanks, and number of fish per tank at the start of the experiment

Group	Salmonid species	Treatment	Number of fish per tank	Number of tanks	Total number of treated fish
1	Atlantic salmon	Infestation	30	3	90
2	Atlantic salmon	Control	30	2	60
3	Coho salmon	Infestation	30	3	90
4	Coho salmon	Control	30	2	60
5	Pink salmon	Infestation	30	3	90
6	Pink salmon	Control	30	2	60
7	Chum salmon	Infestation	30	3	90
8	Chum salmon	Control	30	2	60

In order to achieve lice settlement on all four salmonid species, a total of three consecutive *L. salmonis* immersion challenge trials were conducted on the same batch of fish. The first two challenge attempts were performed on unsedated fish (challenge 1 (13th October 2021), challenge 2 (16th October 2021)). However, the first two attempts did not result in lice attachment on chum salmon and pink salmon. To facilitate a comparative analysis across the four salmonid species, all fish were sedated during the final challenge trial (challenge 3, October 26, 2021), which was conducted as previously described by Jones et al. (2007) and Poley et al. (2018) and described in more detail below. This last challenge trial with sedated fish resulted in lice settlement on all four of the salmonid species, and samples were collected during this trial for downstream analysis. For clarity, all fish in the experiment (except the controls) underwent three challenges with the parasite *L. salmonis*. Details of each challenge trial and samples collected during the third sampling are outlined in the following paragraphs, with a general overview provided in Table 2.

**Table 2:** Overview of three consecutive challenge trials carried out on four different salmonid species. The table summarizes the key parameters of three salmon lice challenge attempts, including the year, date, fish species, number of fish per species, experimental system, sedation method (tricaine methanesulfonate, TMS), challenge method, duration, and the number of lice per fish used across up to three challenge attempts. Recirculating aquaculture system (RAS), flow-through system (FT)

Year	Date	Challenge attempt	Challenged fish, per species	System	TMS	Challenge method	Duration	Number of lice per fish	#Lice/L
2021	13 th of Oct	1	90	RAS	No	100 L container	3 h	82.2	0.88
2021	16 th of Oct	2	75	RAS	No	In fish tank	2 h	149.3	1.1
2021	26 th of Oct	3	66	FT	Yes	10-L baths	15 min	49.8	4.98

*L. salmonis* production and challenge methodologies used a combination of those described by Jones et al. (2007) and Poley et al. (2018). In short, egg strings from adult female lice (*L. salmonis*) were collected from Atlantic salmon farms located in the Bay of Fundy, Atlantic Canada. Copepodids were generated by incubating the strings in a static laboratory seawater system. Concentration and viability of copepodids were validated under a stereo light microscope immediately before each challenge. The copepodids were aliquoted for challenge exposure in each individual tank. The challenge duration and concentration of copepodids per fish were adjusted depending on the challenge test methodology.

The experimental setup of the first and the second challenge involved tanks supplied with recirculating aquaculture system (RAS) saltwater at a concentration of 32–37 ppt. The holding temperature was maintained at 12 ± 1 °C throughout the study. Twenty tanks with a combined capacity of 135 L were used. Each of the salmonid species, pink salmon, coho salmon, chum salmon, and Atlantic salmon, were distributed across five tanks, comprising three infestation tanks and two control tanks. At the start of the experiment, the setup included 30 fish per replicate tank and a total of 150 fish per species. The water flow rate within the experimental tanks was maintained at approximately 2–4 L/min, ensuring one tank exchange per hour. Dissolved oxygen saturation levels were kept between 90 and 120% to ensure adequate oxygenation for the fish. A standard photoperiod of 12 h of light followed by 12 h of darkness simulated natural light conditions and was regulated throughout the study. Fish were fed to satiation once daily with a commercial pelleted diet appropriate for their size. Salinity, temperature, and dissolved oxygen levels were monitored and maintained within the specified ranges. Fish were observed twice daily for mortality and signs of morbidity as part of standard husbandry procedures.

In the first challenge attempt, 12 tanks (30 fish per tank) were infested with *L. salmonis* copepodids. Prior to exposure, fish were netted from their original tanks and equally distributed into 100-L challenge containers. The fish were challenged with 82.2 copepodids per fish in their respective container for 3 h. Additionally, fish from the control tanks were netted and kept in 100-L tanks without lice exposure receiving similar treatment as the infested fish. Supplemental oxygen was added during the infestation procedure to maintain 6.0–9.0 mg/L of oxygen for the 3-h exposure period. After exposure, fish were transferred back to their original tanks. The fish were assessed after 12 h post-exposure (time A, supplemental file 1) for salmon lice. Since the first challenge did not produce significant infestation levels in chum salmon and pink salmon, a second challenge was carried out.

The second challenge followed a similar procedure but reduced the number of fish to 25 per tank due to fish sampled in the first challenge. This time, the fish were exposed to lice directly in their respective tanks, at a lice concentration set to 149.3 lice per fish for 2 h. Despite this, lice counts at 12 hpi and 24 hpi showed low numbers of lice in chum salmon and pink salmon. Hence, a third challenge attempt was then conducted on sedated fish. Due to practical reasons at the facility, fish were transferred from the RAS unit to a flow-through system, maintaining the same tank size, water flow, and light intensity. During the third challenge, fish were anesthetized in their respective tanks with tricaine methanesulfonate (TMS) (100 mg/L). The anesthetized fish (22 per tank) were netted and placed in individual 10-L baths with 1095 *L. salmonis* copepodids (i.e., concentration of 49.8 lice per fish) for 15 min. Dissolved oxygen was supplemented to the 10-L tanks using air-stones during lice exposure. After 15 min in the 10-L baths, the fish, lice, and water were transferred back to their respective tanks.

### Lice counts and sampling of tissue from the third challenge

**Fig. 1:**
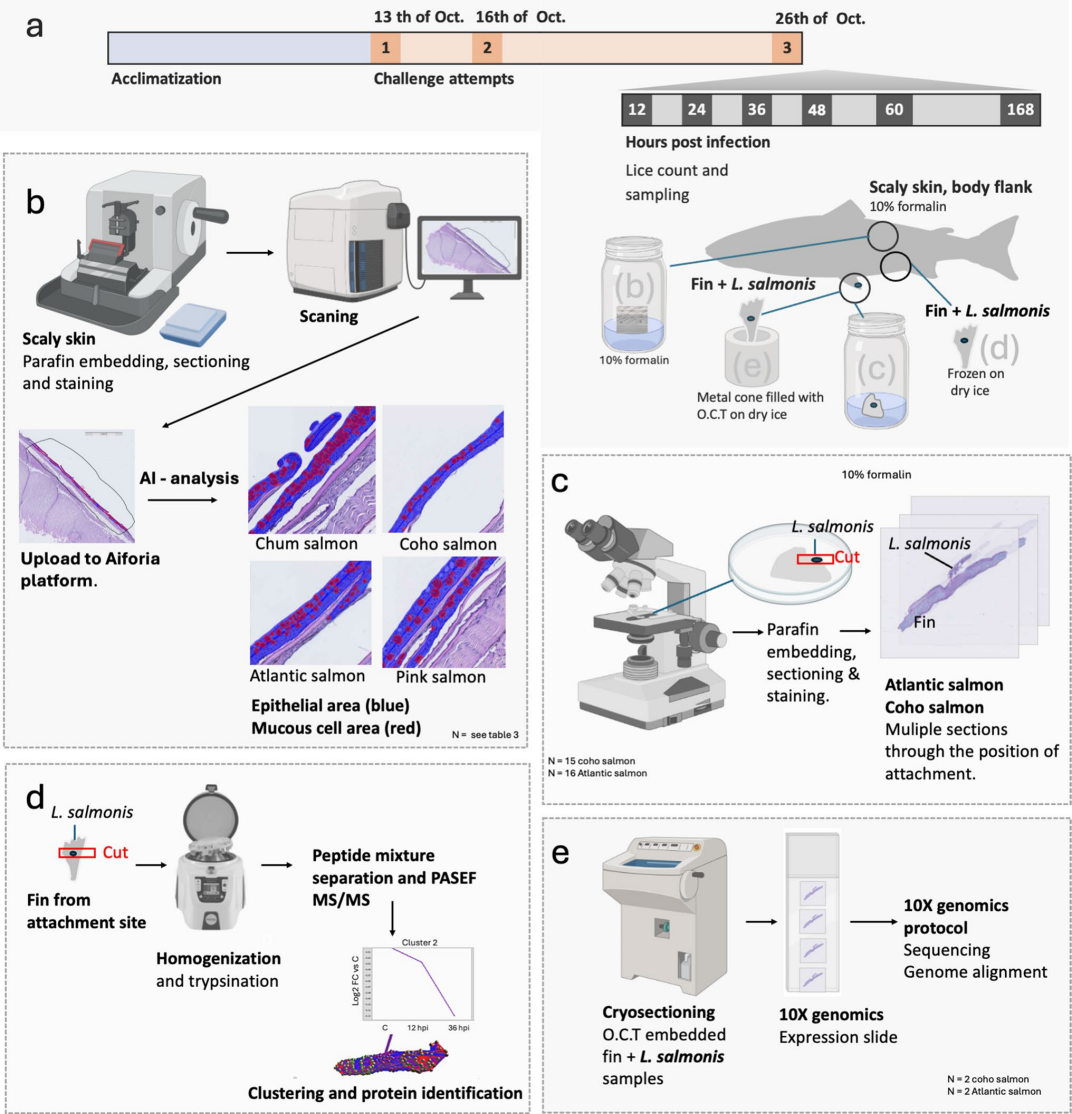
Tissue sampling protocol and downstream analysis for the third challenge trial. **a** Tissue samples were collected at 12, 36, 48, and 60 hours post-infestation, with a final sampling at 168 hpi. At each time point, skin and fin samples were collected for downstream analysis, including quantitative analysis of mucous cells from the scaly skin body flank (**b**), histology of the attachment site (**c**), proteomics of the attachment site (**d**), and spatial transcriptomics of the attachment site (**e**). ** b** Scaly skin from the body flank, with no lice attached, was preserved in 10% neutral buffered formalin for quantitative histological analysis of mucous cell populations. **c** Fins with lice attached were preserved in 10% neutral buffered formalin. The fins were trimmed under a stereomicroscope at the attachment point, prior to paraffin embedding and sectioning. Serial sections were made through the attachment site of lice. **d** Skin with lice attached was homogenized, followed by trypsinization and separation of the fragments. Identification of the peptides was done using PASEf MS/MS for peptide identification. **e** Fins with lice attached were transferred to a metal cone pre-filled with O.C.T. solution and frozen on dry ice. The frozen tissue was sectioned on a cryostat onto 10× Genomics expression slides, following the 10× Genomics protocol for spatial transcriptomic analysis. The figure was generated in PowerPoint (Microsoft) using elements from BioRender

In the third challenge, tissue sampling and lice counts were performed at six time points post-infestation: 12 hpi, 24 hpi, 36 hpi, 48 hpi, 60 hpi, and 168 hpi (Fig. 1). Control samples of naïve fish were collected at 36 hpi and 168 hpi (Table 2). Prior to sampling, the fish were mildly sedated using TMS (Syndel, Nanaimo, BC, Canada) at an anesthetic dose of 100 mg/L, followed by a lethal blow to the head. For each sampling time point, three fish per tank, corresponding to nine fish per species, were randomly selected for sampling, except for the final sampling, where all the remaining fish were sacrificed, with seven fish per tank and 21 fish per treatment. At sampling, each fish was placed in a separate container to facilitate the counting of total lice per fish, including both attached and detached lice. The fish were processed by bleeding (caudal vein puncture; 1-mL syringe with 26-g needle), and the opercula, gill arches, and fins were excised and placed in Petri dishes containing sea water. The attached *L. salmonis* were then counted by life stage and tissue type (body, fins, or gills) using a stereomicroscope, their life stage was recorded, and tissue attachment site was assessed.

Following the lice counts, skin samples were collected from a standardized position on the flank of each fish (skin with scales). In total, 215 skin samples from the body flank of infected and control fish were transferred to 10% buffered formalin (Fisher-Scientific, Mississauga, ON, Canada), intended for quantitively histological analysis of epithelial tissue and mucous cells. Additionally, skin and fin samples with *L. salmonis* attached were collected and transferred to 10% buffered formalin. Note that not all fish had lice; hence, few samples were collected (outlined below). Furthermore, from each fish, one sample with lice (if present) was collected for spatial transcriptomic analysis. The tissue with lice was transferred to a 1.5-cm-long, 8-mm diameter metal tube, filled with O.C.T. Embedding Matrix (CellPath, UK), and held on ice until fully frozen. The samples were then stored at − 80 °C. Samples for histological analysis and spatial transcriptomics were shipped from Canada to Norway using Cencora World Courier™ with temperature loggers. A full list of lice counts and samples collected during the third challenge attempt can be found in supplementary file 1.

### Processing of tissue samples for histological examination

Histological samples of skin and fin were analyzed for two purposes: first, to compare the skin from the body flanks of infected and naïve fish across four salmonid species; and second, to assess the *L. salmoni*s attachment sites in coho salmon and Atlantic salmon (Fig. 1). In total, 217 samples from the body flank of the fish were collected; out of these, 112 were processed for downstream analysis. The samples from body site selected for downstream analysis included tissue from all four salmonid species, from 12 hpi, 36 hpi, and 168 hpi, and control samples from 36 and 168 hpi. For tissue samples intended for the assessment of attachment site, only samples with *L. salmonis* attached on the fixated formalin samples were considered. Some lice detached from the samples during storage in formalin, making it impossible to verify their exact attachment sites. As a result, these samples were excluded from further analysis. As a result, only 30 samples with lice attached were processed for histological examination, 15 samples from coho salmon, and 15 samples form Atlantic salmon. All these 30 samples were trimmed under a stereoscope at the exact position of the lice (~ 3 × 3 mm) before processing and embedding the samples in paraffin.

The embedding, sectioning, and staining of the samples were conducted in the same way for samples collected from the body flank, and fin samples with lice. Sample preparation was carried out at the Veterinary Institute in Harstad, Norway. In short, all tissue samples were embedded in paraffin using LOGOS EVO advanced tissue processor (Milestone, Bergamo, Italy). The paraffin embedded tissue sections were trimmed and sectioned at 2.5 µm. For the samples collected at the body site, one section was mounted on a glass slide per sample. For samples collected at the attachment site, multiple parallel sections were done for each sample. The parallel sections were mounted on three cover glasses, one glass stained with Alcian Blue and PAS, while remaining glass slides were kept for special staining purposes. The multiple sectioning of the tissue at the attachment site left no extra sample material available for additional analysis.

The tissue sections were hydrated in water and stained with 1% Alcian blue (AB, Alfa Aesar) in 3% acetic acid for 15 min, transferred to 1% periodic acid (VWR) for 10 min, followed by Schiffs (Sigma-Aldrich®, Saint-Louis, MO, USA) reagent for 15 min, and finally for 30 s in hematoxylin (VWR, Radnor, PA, USA) before dehydration. The AB/PAS staining differentiates between acidity of the mucins, staining mucous cells blue (acidic), and purple (mixed acidic/neutral) or pink (neutral). MOVAT staining was carried out on two samples from coho salmon with lice, and one sample from Atlantic salmon with lice, following the protocol outlined by Suvarna et al. (2018). All the stained tissue sections were scanned at × 20 with Hammamatsu Nanozoomer (Shizuoka, Japan) S360 and uploaded in the Aiforia® software for visualization. Some selected sections were viewed and photographed at higher magnifications (× 40, × 60, and × 100) with Zeiss Axio Observer Z1 (Zeiss, Oberkochen, Germany). The sections were analyzed by a trained histologist. Information regarding the following parameters was recorded on the scanned slides in a 2 × 2-mm window: presence of lice on the tissue section (yes/no); if lice were present, the direction of the section through the lice body (sagittal or horizontal); estimation of inflammatory cell count with normal morphology (round cell body), categorized as low (10–50), moderate (51–150), high (151–500), or very high (> 500); and estimation of inflammatory cell count with irregular morphology (swollen, fragmented cell body and nuclei), categorized using the same intervals. More information on the histology samples, such as position, time point, and lice stage can be found in supplementary file 1, complemented by a picture of the attachment site for the samples in supplementary file 2.

### Immunohistochemistry

The purpose of the immunohistochemistry (IHC) analysis using primary antibodies for proliferating cell nuclear antigen (PCNA) and tumor necrosis factor alpha (TNF-α) was to investigate the biological responses in the skin of coho salmon infected with *L. salmonis*. PCNA was used to assess cell proliferation, while TNF-α served as a marker for inflammation. IHC analysis with primary antibodies for PCNA and TNF-α (Vertebrate Antibodies, UK) was performed on five selected samples from coho salmon (supplementary file 1). In brief, IHC was carried out by first blocking de-paraffinized tissue sections with horse serum (Merck KGaA, Darmstadt, Germany) for 20 min. This was followed by the application of the primary antibody, either PCNA (dilution 1:60) or TNF-α (dilution 1:2000). The slides were then washed twice in PBS before being incubated with ImmPress AP-anti-Rabbit IgG for TNF-α (Vector Laboratories, CA, USA) or AP-anti-mouse IgG for PCNA (Vector Laboratories) for 30 min. The ImmPACT Vector Red substrate kit (Vector Laboratories) with alkaline phosphatase was used for 30 min according to the protocol, followed by a 30-s counterstain with hematoxylin (Merck KGaA). The slides were visualized as described in the previous section.

### AI-based analysis of skin from body flank

**Fig. 2:**
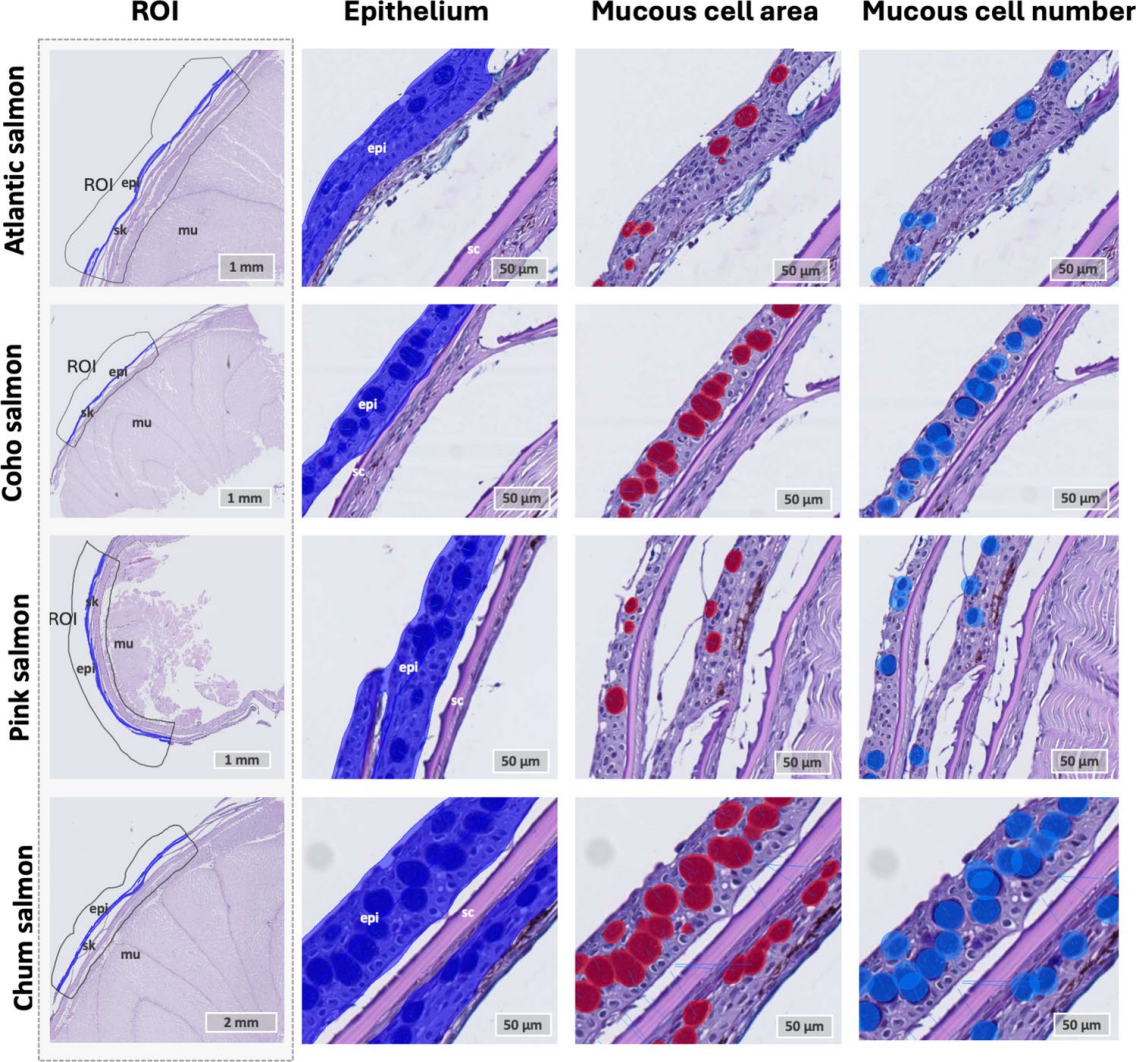
Artificial intelligence-based analysis of skin form body flank. The complete tissue sections from scaly skin were digitized and uploaded into the Aiforia® software, and each section was assigned a region of interest (ROI), avoiding the edges of the samples. The Nofima in-house AI analysis pipeline for skin was applied according to Sveen et al. (2021a, b). The network automatically detected epithelial areas and mucous cell areas by segmentation, and mucous cell numbers by object detection. Artificial color overlays were provided by the AI-algorithm post-analysis. In the figure, epithelial tissue is marked dark blue, mucous cell area red, and object detection of mucous cells blue

Skin samples from the body flank were analyzed using Nofima’s skin-AI model on the Aiforia® platform. In short, the analysis uses a neural network that automatically segments multiple features of the skin based on a manually annotated training set, as described by Sveen et al. (2021b) and Fig. 2. The AI analysis was conducted on tissue samples collected from the body flank of all four salmonid species at the time points outlined in Table 3. Note that the number of samples per time point varied as it was difficult to collect superior-quality skin samples for all the four fish species during the very intensive sampling routine. As such, skin samples which after processing did not show excellent quality, meaning that all skin layers were intact, were excluded from the AI-analysis.

**Table 3:** Sample distribution for AI-histological analysis of fish skin from body flank. The table shows salmonid species, number of control samples, and number of infested samples

Species	Control (*N*)	Lice infested samples—12 hpi	Lice infested samples- 36 hpi	Total number of infested samples
Atlantic salmon	6	4	5	9
Chum salmon	10	8	6	14
Coho salmon	9	6	7	13
Pink salmon	10	7	8	15

On the Aiforia® platform, a region of interest (ROI) was manually assigned to each section, excluding the edges of samples and areas at the edges of the tissue section which often contain artifacts such as cracks and discoloration. The Nofima in-house AI model, fish skin 5.2 released in 2019, was used on the samples (Table 3). After the analysis, a trained histologist manually assessed the color overlay presented by the model to determine which features were assigned to each class. The overlay of the detected areas was satisfactory, and the AI model developed for Atlantic salmon skin effectively detected similar features in Pacific species. The identified epithelial tissue included the total area of keratinocytes and secretory cells of the epithelium (mucous cells and sacciform cells). Additionally, the overlay of the mucous cell area matched well with the mucous cells, and the mucous cell detector classified mucous cells as objects for numerical registration. Since the skin of the four salmonid species appears similar to the naked eye, with no major visual differences, the AI model was able to detect similar features across species.

After performing AI analysis using Aiforia®, the results were exported in CSV format and further analyzed in R (version 4.2.2), as outlined by Sveen et al. (2021b). The samples were tested for significance between infested and control samples within each species, for 12 hpi vs. control, 36 hpi vs. control, and a combined approach (12 hpi + 36 hpi) vs. controls (ANOVA, *p* > 0.05), no significant findings were found. Species differences were analyzed using ANOVA. Where significant differences were detected (*p* < 0.05), Tukey post hoc tests were performed, with group differences indicated by lower-case letters in the figures. Groups not sharing a letter were significantly different (*p* < 0.05, TukeyHSD, R stats package). Data visualization was done using custom functions in base R and the beeswarm package, with statistical results displayed at the top of each chart using the aov function from R’s stats package. Groups not sharing a letter were significantly different (*p* < 0.05, TukeyHSD in R’s stats package). The results were visualized using custom functions in base R and the beeswarm package. Statistical results were added at the top of each chart using the aov function from the stats package in base R.

### Proteomic analysis of skin samples

Frozen coho pectoral fin samples with lice attached (*n* = 3) and Atlantic salmon pectoral fin samples (*n* = 4) with lice attached, and within each species, pectoral fin at both 12 hpi (*n* = 3 in both species), and 36 hpi (*n* = 3 in both species) after infection with *L. salmonis* were dissected from the area of the lice attachment site and homogenized in Precellys CKMix ceramic beads vials in lysis buffer (5% SDS, 50 mM TEAB) at an approximately 1:10 ratio of sample weight in mg to volume buffer in microliter. Tryptic digestion was performed using a protocol modified from Protifi S-Trap spin columns, with reduction performed using 25 mM DTT, alkylation with 10 mM IAA, and digestion with porcine trypsin (Promega) at an enzyme to protein ratio of 1:10. For each sample, 200 ng of tryptic peptide mixtures was separated onto a 75 μm × 250 mm IonOpticks Aurora 3 C18 column (Ion Opticks Pty Ltd., Australia). A gradient of basic reversed-phase buffers (Buffer A: 0.1% formic acid, 98% H_2_O, 2% acetonitrile; Buffer B: 0.1% formic acid, 100% acetonitrile) was run on a Ultimate 3000 RSLCnano (Thermo) at a flow rate of 200 nL/min at 50 °C. Data was acquired for 60 min along a gradient on tims TOF HT (Bruker Daltonik GmbH, Bremen, Germany) with a Captive Spray ion source (Bruker Daltonik) using a diaParallelAccumulationSerialFragmentation() acquisition method consisting of 12 cycles including a total of 32 mass width windows (27.2 Da width, from 400 to 1201 Da) with 2 mobility windows each, making a total of 64 windows covering the ion mobility range (1/K_0_) from 0.65 to 1.45 V s/cm^2^. Optimal m/z and ion mobility windows were designed with py_diAID utility using a DDA-PASEF run previously acquired from a pool of the analyzed samples. For DDA-PASEF acquisition, the full scans were recorded from 100 to 1700 m/z spanning from 1.45 to 0.65 Vs/cm^2^ in the mobility (1/K0) dimension. PASEF MS/MS frames were performed on ion-mobility separated precursors, excluding singly charged ions which are fully segregated in the mobility dimension, with a threshold and target intensity of 1750 and 14,500 counts, respectively. In both DDA- and DIA-PASEF modes, the collision energy was ramped linearly from 59 eV at 1/k0 = 1.6 to 20 eV at 1/k0 = 0.6.

Raw mass spectral data were processed using Spectronaut Version 18.0 (Biognosys, Switzerland) with the directDIA workflow. Initially, a database search was conducted using the Pulsar search engine against Ensembl *O. kisutch* (118,040 entries) or *S. salar* (146,172 entries) database. For Pulsar search and spectral library generation, the following parameters were applied: Trypsin/P as the proteolytic enzyme, specific cleavage mode, peptide length range of 7–52 residues, oxidation of methionine and N-terminal acetylation as variable modifications, carbamidomethylation of cysteine as a fixed modification, and a 1% false discovery rate (FDR) at the PSM, peptide, and protein group levels. The generated spectral library was then utilized by Spectronaut for DIA analysis, with quantification performed at the MS2 level based on extracted ion chromatogram (XIC) areas. An automatic local cross-run normalization strategy was applied, and protein quantification was carried out using the MaxLFQ algorithm.

In Atlantic salmon samples, 8172 proteins were consistently identified with two or more unique peptides in at least two replicates per time point throughout the infestation time course, while in coho salmon samples, 7719 proteins met the same criteria. For each species, raw intensities corresponding to protein abundances were transformed, median-normalized, and analyzed by two-tailed *t*-test per timepoint to calculate differentially expressed proteins (DEPs) at 12 hpi vs. uninfected controls and at 36 hpi vs. uninfected controls. Significantly differentially expressed proteins (*p* < 0.05) for the 36 hpi vs. control (Atlantic, *n* = 889; coho salmon = 690 proteins) or both 12 hpi and 36 hpi vs. uninfected control comparisons (Atlantic salmon, *n* = 296; coho salmon = 149 proteins) were subjected to expression profile clustering in BioLayout*Express*^*3D*^ (Theocharidis et al. 2009) (Pearson correlation threshold minimum = 0.7, *R*^2^ = 0.95) to identify host proteins exhibiting a consistent increase or decrease over infestation time course in their relative expression levels from challenge onset. Isolated clusters representing biologically relevant alterations throughout infestation time course were then mapped from either *S. salar* or *O. kisutch* Ensembl Protein to Ensembl Gene namespaces in g:Convert (Kolberg et al. 2023), and then to murine (*Mus musculus*) gene orthologues. For all inputted *S. salar* and *M. musculus* inputted Ensembl Protein namespaces, an equivalent Ensembl Gene namespace was identified; if multiple murine orthologues were predicted based on single fish Ensembl Gene search, then top orthologue was selected.

Datasets constituting host (*S. salar* or *O. kisutch*) proteins exhibiting statistically significant increase or decrease in expression over infestation time course, with accompanying murine orthologues, were then subjected to pathway analysis using Ingenuity Pathway Analysis (Qiagen) software. For all pathway analyses performed, *p*-values of canonical pathway scores and subsequent rankings were derived from Fisher’s exact test calculating overlap between molecules in each respective input dataset vs. the number of molecules comprising canonical pathway as defined by Ingenuity Systems Database. Predicted activation *z*-scores were calculated by weighing the predicted expression change of target molecules as defined by Ingenuity Knowledge database against the actual expression change of target molecules reported in input dataset. An activation *z*-score > 2 (indicated by orange coloration in graphical representation) or < − 2 (indicated by blue coloration in graphical representation) is considered statistically significant.

### Spatial transcriptomics at the attachment site

In total, four samples, two coho salmon fins, and two Atlantic salmon fins with *L. salmonis* attached, were processed for spatial transcriptomics. The Visium protocol (Visium Spatial Protocols Tissue Preparation Guide, CG000240 RevB) was slightly modified to address challenges encountered during sample sectioning, as described by a previous protocol developed for fish skin and fins (Sveen et al. 2023). In short, fin with, lice were transferred to a metal cylinder and embedding medium Tissue-Tek (Sakura Finetek, USA) optimal cutting temperature (O.C.T.) compound, was added to the cylinder before freezing the entire cylinder on dry ice (Fig. 1). The samples were then shipped to Nofima AS on dry ice, as previously described. The embedded tissue was removed from the cylinder by slightly warming the cylinder in the hand and removing the embedded sample from the cylinder by pressing the solid O.C.T media with a metal object. The tissue, still embedded and frozen in O.C.T., was mounted on a cryostat sample holder and sectioned at 10 µm. As the fin samples were embedded in O.C.T., which is white and non-transparent solid when frozen, it was not possible to detect the lice during sectioning. Hence, two scientists collaborated during the sectioning process to obtain tissue sections with fish tissue and lice attached. One scientist sectioned the O.C.T. embedded samples while the other examined each slide for the presence of tissue and lice. During assessment of the samples, serial sections were created through the sample material, and the frozen slides were briefly fixed in formalin for 10 s, rinsed with water, and immersed in hematoxylin for 3 s, followed by morphology assessment under a microscope. Once a section containing salmon tissue and what was believed to be *L. salmonis* was identified, adjacent frozen tissue sections were transferred to the Visium expression slide (10 × Genomics) and stored at − 80 °C until hematoxylin and eosin staining. In total, four different samples were added to the slide, two samples from coho salmon fin with lice, and two samples with Atlantic salmon with lice. Tissue staining and library preparation were performed according to the Visium Spatial Gene Expression User Guide (10 × Genomics).

Libraries were sequenced on NovaSeq 6000 SP flow cell (Illumina, USA) at the Norwegian Sequencing Center as 50 bp paired end reads. Sequencing was done using the following cycles: read 1, 28 cycles, i7 Index; 10 cycles, i5 Index; 10 cycles; and read 2, 90 cycles (Visium Spatial Gene Expression User Guide; 10 × Genomics). Reads were aligned to the Atlantic salmon genome (version Ssal_v3.1, INSDC Assembly GCA_905237065.2), coho salmon Genome build Okis_V2, accession number GCA_002021735.2, and *L. salmonis* Genome build Uvic_Lsal_1.0, accession number GCA_016086655.3 using the software Space Ranger (version 1.3.1; 10 × Genomics, USA). Sequence data have been submitted to NCBI’s Sequence Read Archive (SRA) as BioProject PRJNA1149653. High-resolution JPG images (supplementary file 2) from each of the associated tissue sections were aligned to the reads by default settings. Optimal number of tissue clusters and cluster membership of spots was defined using graph-based clustering (modified python implementation of the augmented implicitly restarted Lanczos bidiagonalization algorithm (IRLBA) (Baglama and Reichel 2005)) in Space Ranger. Transcripts defining tissue clusters were defined as those showing upregulated transcripts (relative to the other clusters), adjusted *p*-value (Benjamini–Hochberg procedure) < 0.1, and mean barcoded unique molecular identifier (UMI) count > 1. Expression levels of genes were visualized using 10 × Genomics Loup Browser v8.0.0. Fine tuning of clustering was done using k-means of four with cluster 4 representing manual annotation of the attachment site in 10 × Genomics Loup Browser v8.0.0.

## Results and discussion

Pacific salmon species exhibit varying levels of resistance to *L. salmonis* compared to the highly susceptible Atlantic salmon. Thus, this study aimed to characterize the skin mucosa of four different salmonid species with varying susceptibility to *L. salmonis*. Our toolbox comprised a variety of molecular techniques, including AI-driven histological analysis of fish skin, manual histological assessment of the attachment site, proteomics, and spatial transcriptomics. Overall, our results show that mucous cell density might be important for the initial attachment success of the salmon lice, while the influx of neutrophils towards the copepodid attachment site, accompanied by neutrophil degranulation, is the likely driver of copepodid rejection in coho salmon. The results and discussion leading to these conclusions are presented below.

### Lice counts

The goal of the challenge trials was to achieve sufficient lice infestation levels to collect tissue samples for histological analysis from all four salmonid species. After the first two lice challenge exposures, no copepodids were found on any pink salmon or chum salmon and only a limited number of copepodids found on Atlantic salmon (mean = 8.0 ± 6.4 SD) and coho salmon (mean = 1.0 ± 1.0 SD). Hence, a third challenge attempt was performed with fish being anesthetized, which resulted in lice infestation in all four species. At the third challenge, during the 12–48 hpi time interval, 2–30% of the registered lice on Atlantic salmon were chalimus stage, suggesting that the fish still had lice remaining from the initial challenge trials (Table 4). In the same time interval, only one coho salmon and one chum salmon had 1 chalimus left on the body, and one chalimus was found on one chum salmon fin. Furthermore, in Atlantic salmon, lice were predominantly found on the gills at all time points, with the highest counts at 168 hpi (6.89 lice per fish). Fins and body also showed attachment, particularly at 168 hpi (3.17 and 3.06 lice per fish, respectively). Chum salmon exhibited low lice counts overall, with the highest attachment on fins and gills at 12 hpi (0.56 and 1.72 lice per fish, respectively), but no lice were detected by 168 hpi. Coho salmon showed high lice attachment on fins, peaking at 48 hpi (3.28 lice per fish), but minimal attachment on the body and gills. By 168 hpi, coho salmon had nearly cleared the infestation, with only 0.67 lice per fish on fins. Pink salmon had the lowest lice counts overall, with minimal attachment on fins and gills at early time points (e.g., 0.89 lice per fish on gills at 12 hpi) and no lice detected by 168 hpi.

**Table 4:**
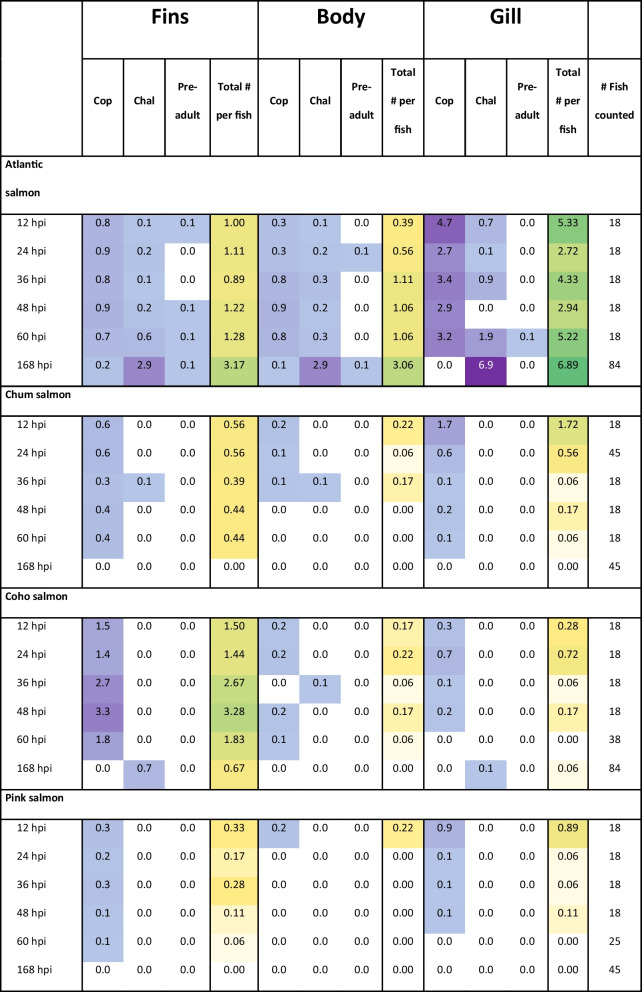
Number of lice per fish species and time point for the third challenge trial. The lice numbers are presented separately for copepodid (cop), chalimus (chal) and pre-adult lice stage for the three different body positions fins, main body and gill. The numbers represent average lice count per fish, and positive lice counts are marked in shades of purple, with darker shades indicating higher counts. The total number of lice per fish (total # per fish, copepodid + chalimus + pre-adult) is also indicated, positive numbers are marked in yellow to green shades with dark green indicating higher total counts of lice. The total number of fish counted is also indicated in the table

The initial attempts to infest unsedated chum salmon and pink salmon failed; as a result, three consecutive challenge trials were conducted using the same batch of fish. This led to a carry-over of salmon lice from previous trials, particularly on Atlantic salmon. The use of anesthesia in the third challenge trial resulted in a higher proportion of lice settlement on the gills. The lice settlement on the gills was most pronounced in Atlantic salmon, where lice counts were higher compared to the fins or body throughout the experiment. Although it appears that sedation during lice infestation promotes lice settlement on the fins and the gills in all the salmonid species under investigation, sedation does not seem to impact the overall response dynamics of the host against the lice. The Pacific species rejects the lice from fins and gills, shortly after infection, while Atlantic salmon does not. Due to the carry-over of lice from the first and second challenge trials in this study, the experimental design cannot be used to draw detailed conclusions regarding lice numbers on the four salmonid species. Nevertheless, consistent with multiple previous studies (Fast et al. 2002a; Johnson & Albright 1992b; Jones et al. 2007; Sutherland et al. 2014), our results demonstrate that the Pacific species investigated can reject most copepodid lice before they reach the chalimus stage, while Atlantic salmon do not exhibit this rejection capability, allowing lice counts to persist.

### Quantitative assessment of mucous cells in the scaly skin from the body flanks reveals differences among the four salmonid species

**Fig. 3:**
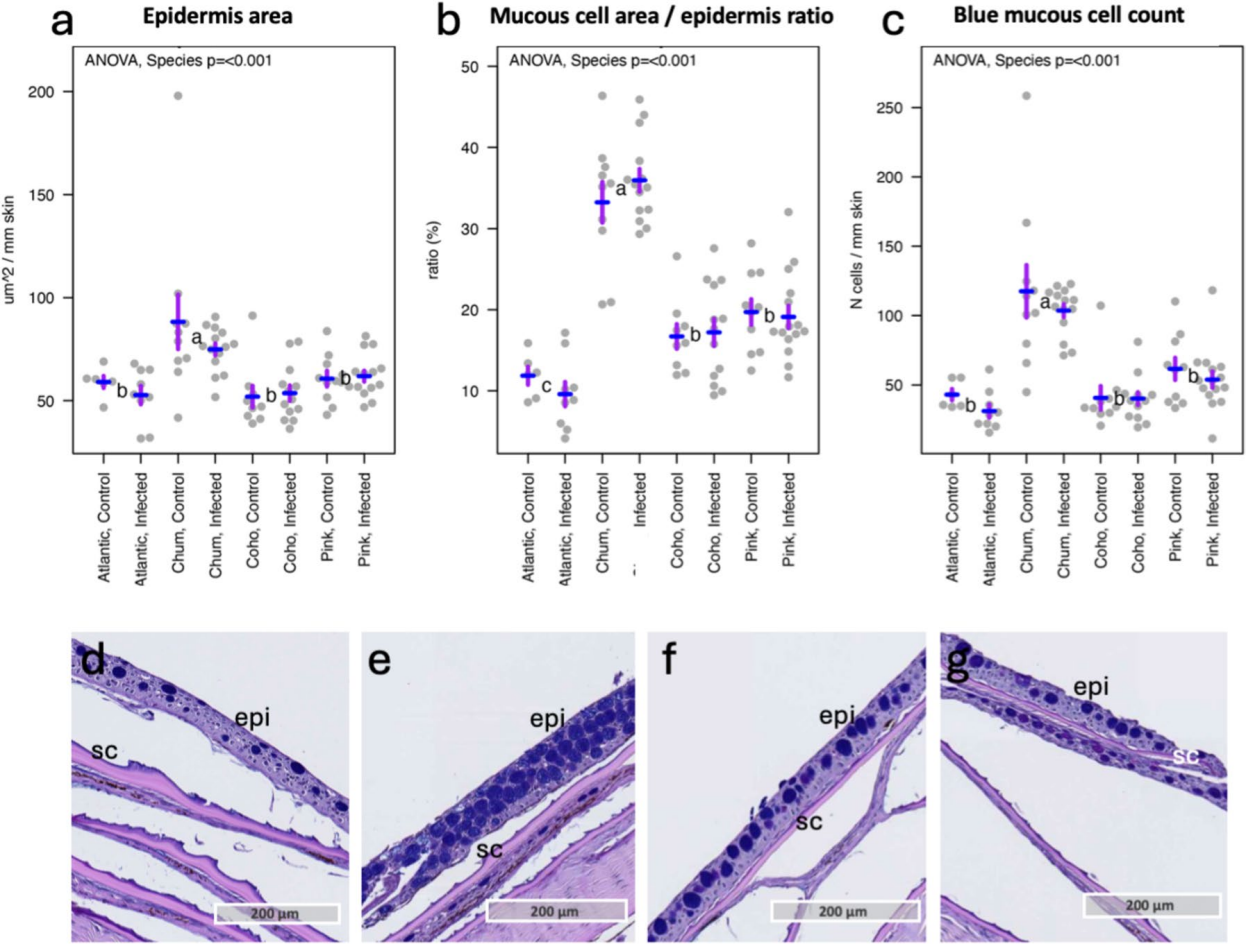
Quantitative assessment of epidermal area and mucous cells in the skin of four salmonid species. **a** Epidermal area. **b** Ratio of mucous cell area to epidermal area. **c** Number of blue-stained mucous cells per mm of skin. ANOVA *p*-values for species are indicated at the top of each plot. Significant differences between the species were determined using Tukey’s post hoc test at *p* < 0.05; groups not sharing a letter (positioned in between their respective control and infested values) were significantly different. Each of the species were tested for differences between control and lice infested samples, but no significant differences were found between these groups (*t*-test, *p*-value > 0.05). Representative Alcian Blue and Periodic Acid-Schiff-stained tissue sections of skin from the body flank of each species: **d** Atlantic salmon, **e** chum salmon, **f** coho salmon, and **g** pink salmon. Abbreviations: epi epithelium, sc scale

Since salmon lice settlement was only achieved for sedated chum salmon and pink salmon, this raised the question whether differences in skin mucosa could potentially explain the variations in lice settlement. Thus, our analysis concentrated on characterizing the scaly skin of the body flank across all four salmonid species, with a focus on mucous cell population, mucous cell area, and mucous cell number in the skin. Overall, the results showed no significant differences in epithelial area or mucous cell count between infested and control samples within each species (Fig. 3). However, there were differences between species. Chum salmon had the largest area of epithelial tissue per mm of skin, with 30–40% of the epithelial area consisting of mucous cells, corresponding to more than 100 mucous cells per mm of skin. Additionally, mucous cells accounted for 15–20% of the epithelial surface in pink and coho salmon, while Atlantic salmon showed the lowest proportion, with only 10% coverage. Based on our findings, we suggest that mucous cells, potentially in combination with behavioral mechanisms (disrupted by sedation), may offer protection against lice settlement in chum and pink salmon. In Atlantic salmon, mucous cell number and density in the epithelium are influenced by feeding, with some evidence for reduced lice numbers with more mucous cells (Kaur et al. 2024; Sveen et al. 2021a). Furthermore, factors beyond mucous cell count, such as variations in the presence of immunological defense molecules (Esteban 2012) or organic compounds (Difford et al. 2022) in the fish mucus, could contribute to *L. salmonis* resistance and warrant further investigation.

### Assessment of the attachment site of *L. salmonis*

Given that *L. salmonis* successfully attached to the fins of both Atlantic salmon and coho salmon, but only coho salmon displayed copepodid rejection, the investigation focused on a more detailed analysis of the attachment sites in these two species. First, a qualitative overview of the obtained sections was conducted (Table 5). Out of sixteen slides from the attachment site, only five coho salmon slides contained lice tissue. Among these, the entire louse was captured in sagittal section across two slides. In contrast, ten Atlantic salmon slides contained lice tissue, and seven of these captured a sagittal section of the entire louse. Based on the size of the lice and the recorded lice stage at sampling, three copepodids and four chalimus were identified in Atlantic salmon, while all lice in coho salmon were copepodids. It is possible that lice, particularly copepodids, fell off during processing, as they are not as firmly anchored to the tissue as the chalimus stage, which attaches securely via a frontal filament (Gonzalez-Alanis et al. 2001). Although lice tissue was not captured in all sections, the slides are referred to as attachment sites, as the sections were trimmed close to the lice positions before processing of the collected fin and body samples.

**Table 5:** Overview of the examined tissue sections collected from the lice attachment sites in coho salmon and Atlantic salmon. The table indicates whether lice tissue was present on the examined slides, whether the entire louse was captured, the plane of sectioning, the size of the lice, and the most likely lice stage based on morphology. Additionally, the number of inflammatory cells and inflammatory cells with irregular morphology at the attachment site (2 × 2 mm frame) is provided. Slide IDs are included, and the zone of attachment can be viewed in supplementary file 2

Species	hpi	Tissue type	Lice tissue on slide	Whole lice captured	Plane of sectioning lice	Size of lice tissue on slide	Lice stage based on tissue morphology	# inflammatory cells	# inflammatory cells with irregular morphology	Slide-ID
Coho salmon	12	Fin D	Yes	No	Horizontal	Na	Copepodid	> 10	0	33
Coho salmon	12	Fin D	No	No	Na	Na	Na	> 10	0	35
Coho salmon	12	Fin pelvic	No	No	Na	Na	Na	> 50	0	36
Coho salmon	24	Fin PC	Yes	No	Na	Na	Na	> 10	0	66
Coho salmon	36	Fin C	Yes	Yes	Saggital	0.38 mm	Copeopodid	> 10	0	100
Coho salmon	36	Fin C	No	No	Na	Na	Na	> 500	> 500	101
Coho salmon	36	Fin PC	Yes	Yes	Saggital	0.68 mm	Copeopodid	> 10	0	108
Coho salmon	36	Fin C	No	No	Na	Na	Na	> 150	> 150	135
Coho salmon	48	Fin C	No	No	Na	Na	Na	> 500	> 500	136
Coho salmon	48	Fin PC	No	No	Na	Na	Na	> 10	0	137
Coho salmon	48	Fin PC	No	No	Na	Na	Na	> 10	0	139
Coho salmon	48	Fin PC	Yes	No	Horizontal	Na	Na	> 200	> 200	141
Coho salmon	48	Fin D	No	No	Na	Na	Na	> 50	0	142
Coho salmon	48	Fin C	No	No	Na	Na	Na	> 15	0	143
Coho salmon	48	Fin AD	No	No	Na	Na	Na	> 50	> 50	175
Atlantic salmon	12	Caudal fin	Yes	Yes	Sagittal	0.55 mm	Copepodid	> 10	0	1
Atlantic salmon	12	Scaly skin	No	No	Na	Na	Na	> 10	0	7
Atlantic salmon	12	Scaly skin	Yes	Yes	Sagittal	11 mm	Chalimus	> 10	0	8
Atlantic salmon	24	Scaly skin	No	No	Na	Na	Na	> 10	0	39
Atlantic salmon	24	Scaly skin	Yes	Yes	Sagittal	12 mm	Chalimus	> 50	0	41
Atlantic salmon	24	Scaly skin	No	No	Na	Na	Na	> 10	0	45
Atlantic salmon	36	Scaly skin	Yes	Yes	Sagittal	12 mm	Chalimus	> 100	0	74
Atlantic salmon	36	Scaly skin	Yes	Yes	Sagittal	0.78 mm	Copepodid	> 50	0	77
Atlantic salmon	36	Scaly skin	No	No	Na	Na	Na	> 10	0	80
Atlantic salmon	36	Fin anal	Yes	No	Horizontal	Na	Na	> 10	0	80
Atlantic salmon	48	Pelvic fin	Yes	No	Sagittal	Na	Na	> 10	0	114
Atlantic salmon	48	Fin caudal	No	No	Na	Na	Na	> 10	0	116
Atlantic salmon	60	Pelvic fin	Yes	Yes	Sagittal	17 mm	Chalimus	> 10	0	145
Atlantic salmon	60	Operculum	No	No	Na	Na	Na	> 10	0	148
Atlantic salmon	168	Fin caudal	Yes	Yes	Sagittal	1 mm	Copepodid	> 10	0	160
Atlantic salmon	168	Scaly skin	No	No	Na	Na	Na	> 10	0	160
Atlantic salmon	168	Fin	Yes	No	Sagittal	Na	Na	> 10	0	163

### Morphological and inflammatory responses at the *L. salmonis* attachment site in coho salmon

Next, we estimated the number of inflammatory cells at the attachment site within a 2 × 2 mm area on the sections (Table 5). Across four samples analyzed between 12 and 24 hpi in coho salmon, cell counts ranged from 10 to 50. At later time points (36–48 hpi), four samples exhibited inflammatory cell counts exceeding 150 at the attachment site, with most cells displaying irregular morphology.

**Fig. 4:**
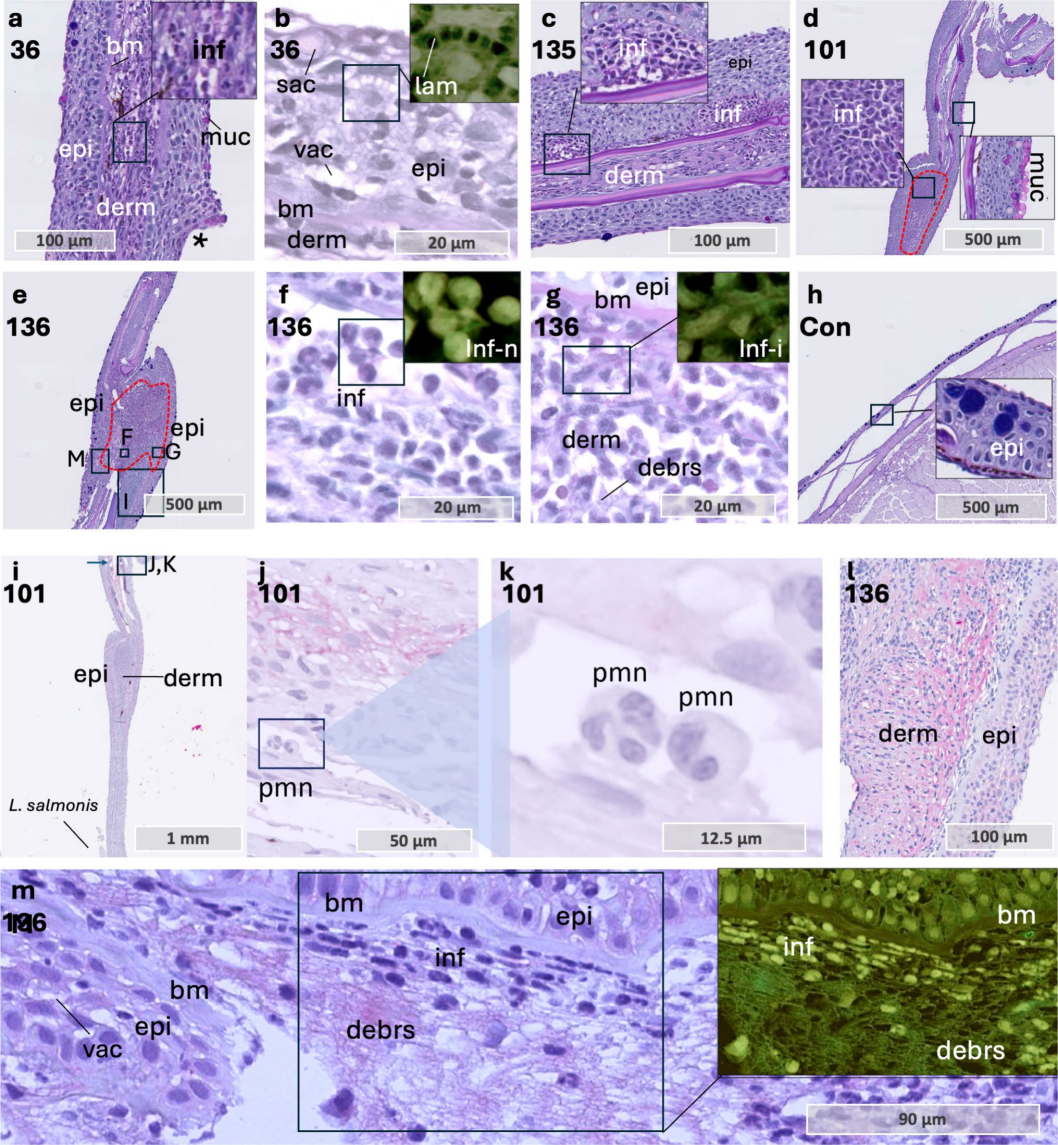
Tissue sections through the attachment site in coho salmon. **a** Pelvic fin at 12 hpi showing inflammatory cells in the dermis. The marked area highlights these cells, with an enlarged view in the upper right. The *L. salmonis* attachment site is marked with a star. Sample 36. **b** Epithelial tissue showing vacuolization due to lamellipodia extension. The marked area is magnified in the upper right (inverted colors), illustrating this process in keratinocytes. Sample 36. **c** Caudal fin at 48 hpi with two dermal compartments with inflammatory cells. One of the compartments is shown in a magnified view in the upper right corner. Sample 135. **d** Pelvic fin at 36 hpi showing a high density (> 500) of inflammatory cells in the dermis (red marking). Magnified views highlight two regions: one with epidermal pink-staining mucous cells, and the other with inflammatory cells in the dermis. Sample 101. **e** Pelvic fin at 36 hpi showing a high density (> 500) of inflammatory cells in the dermis (red marking). Sample 136. **f** Magnified view of sample **e**, showing inflammatory cells with a round, neutrophil-like shape. The marked area is magnified in the upper right (inverted colors) to highlight the circular-shaped nuclei (light green). **g** Magnified area of sample **e**, showing details of irregularly shaped inflammatory cells close to the epithelial border. The marked area is magnified in the upper right (inverted colors) to highlight the irregular shaped nuclei of these inflammatory cells. **h** Control sample from coho salmon, magnified area shows normal epithelium with densely packed keratinocytes. **i** Full scan of sample 101. Note that *L. salmonis* is in the distal part of the photo, while diffuse positive red staining for TNF-α is present in the dermal tissue, far from the louse attachment site (arrow). **j** and **k** Details of inflammatory cells with neutrophil morphology in dermal compartment. The magnified area is marked in picture. **l** Positive red staining in the dermal compartment. This section is a parallel section of the same sample as viewed in **e**, with the magnified area marked by a rectangle in **e**. **m** Diffuse positive red staining in the dermal compartment. Co-localization of debris and irregular shaped inflammatory cells. Magnified square in the upper right corner (inverted colors). This section is a parallel section of the same sample as viewed in **e**, with the magnified area marked by a rectangle in **e**. Abbreviations: bm basal membrane, con control, debrs debris, derm dermis, epi epithelium, inf inflammatory cells, n normal shape, i irregular shape, lam lamellipodia, muc mucous cell, sac sacciform cell, vac vacuolization. Alcian Blue and PAS-stained tissue sections (**a**–**h**) and immunohistochemistry with anti TNF-α (**i**–**m**). Color inversion was applied on selected photos to enhance contrast and highlight structural differences, with cell nuclei shown in light green

Furthermore, inflammatory cells were present in the dermal compartment at 12 hpi (Fig. 4 a), coinciding with epithelial vacuolization caused by lamellipodia formation between adjacent cells (Fig. 4 b). Epithelial vacuolization is a hallmark of the immediate wound healing response in salmon skin following mechanical damage (Sveen et al. 2019, 2018). Therefore, its presence at 12 hpi suggests an early activation of epithelial cells in response to infestation. Furthermore, dermal aggregates of inflammatory cells were observed in multiple coho salmon samples at 36–48 hpi (Fig. 4 c–g). Some of the inflammatory cells in these aggregates had a normal morphology with a rounded cell body, while others exhibited irregular and fragmented shapes, indicative of degranulation or cellular stress (Donovan et al. 2021).

To confirm ongoing inflammatory responses in coho salmon, immunohistochemistry for TNF-α was performed on two samples (Fig. 4 i–n). Diffuse red staining, indicative of TNF-α presence, was primarily observed in the dermal compartment of both samples but was absent in dermal blood vessels. Additionally, inflammatory cells detected within dermal blood vessels suggested neutrophil recruitment to the dermal compartment via the bloodstream. These intravascular cells exhibited neutrophil-like characteristics, including a rounded cell body with a multi-lobed nucleus (Donovan et al. 2021; Roberts & Hallett 2019). In contrast, irregularly shaped inflammatory cells were present within the dermal compartment, accompanied by amorphous debris and diffuse TNF-α staining.

**Fig. 5:**
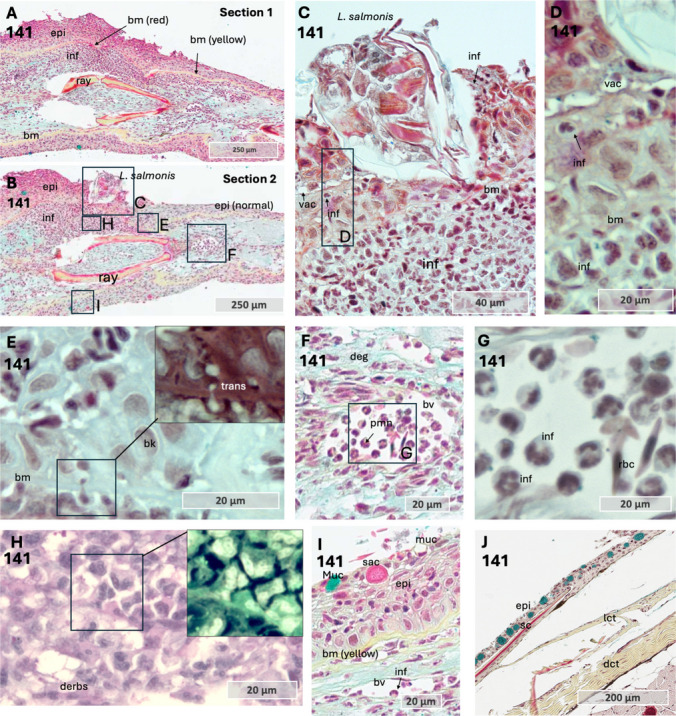
Serial sections through the attachment site in coho salmon at 36 h post-infestation (hpi), sample 141. **a** Tissue section immediately anterior to the head of *L. salmonis*. **b** Same sample as in **a**, showing a section directly through the head of *L. salmonis*, which is embedded in the epithelial tissue of coho salmon fin. In **a** and **b**, note the color and structural change of basal membrane, thin and red under the attachment site, and then yellow staining at increasing distance from the lice. **c** Magnified view of the *L. salmonis* head, with inflammatory cells in both the dermal and epithelial compartment, along with vacuolization and hyalinization of the epithelium. This image is from the same section as **b**, mirrored due to imaging with the Zeiss AXIO Observer at × 60 magnification. **d** Details of the epithelial tissue close to the head of the lice, showing inflammatory cells in the epithelial tissue and vacuolization of the epithelium. **e** Intersection between epithelial and dermal tissues, showing one inflammatory cell translocating from the dermal to the epidermal compartment through the basement membrane (magnified, and inverted color). Area of magnification is marked in **b**. **f** Blood vessel containing inflammatory cells with typical neutrophil characteristics. **g** Magnified view of the rectangular area in **f**. with inflammatory cells and red blood cells. **h** Inflammatory cells with irregular morphology were present in the dermal compartment beneath the attachment of the lice. Magnified view of irregular shaped inflammatory cell in upper right corner, inverted colors. The magnified area marked in section **b**. **i** Details of epithelial tissue on opposite side of attachment, with mucous cell staining blue and translucent, and vesicle of a sacciform cell with red/pink staining. **j** Control sample of Atlantic salmon skin. All photos (except **h**, Alcian Blue and PAS) were stained with MOVAT pentachrome, which distinguishes collagen (yellow), glycans (blue), fibrin (bright red), and muscle (red). Abbreviations: bv blood vessel, bm basement membrane, dct dense connective tissue, epi epithelium, ray fin ray, inf inflammatory cells, lct loose connective tissue, sc scale, vac vacuolization. Color inversion was applied on selected photos to enhance contrast and highlight structural differences, with cell nuclei shown in light green

**Fig. 6:**
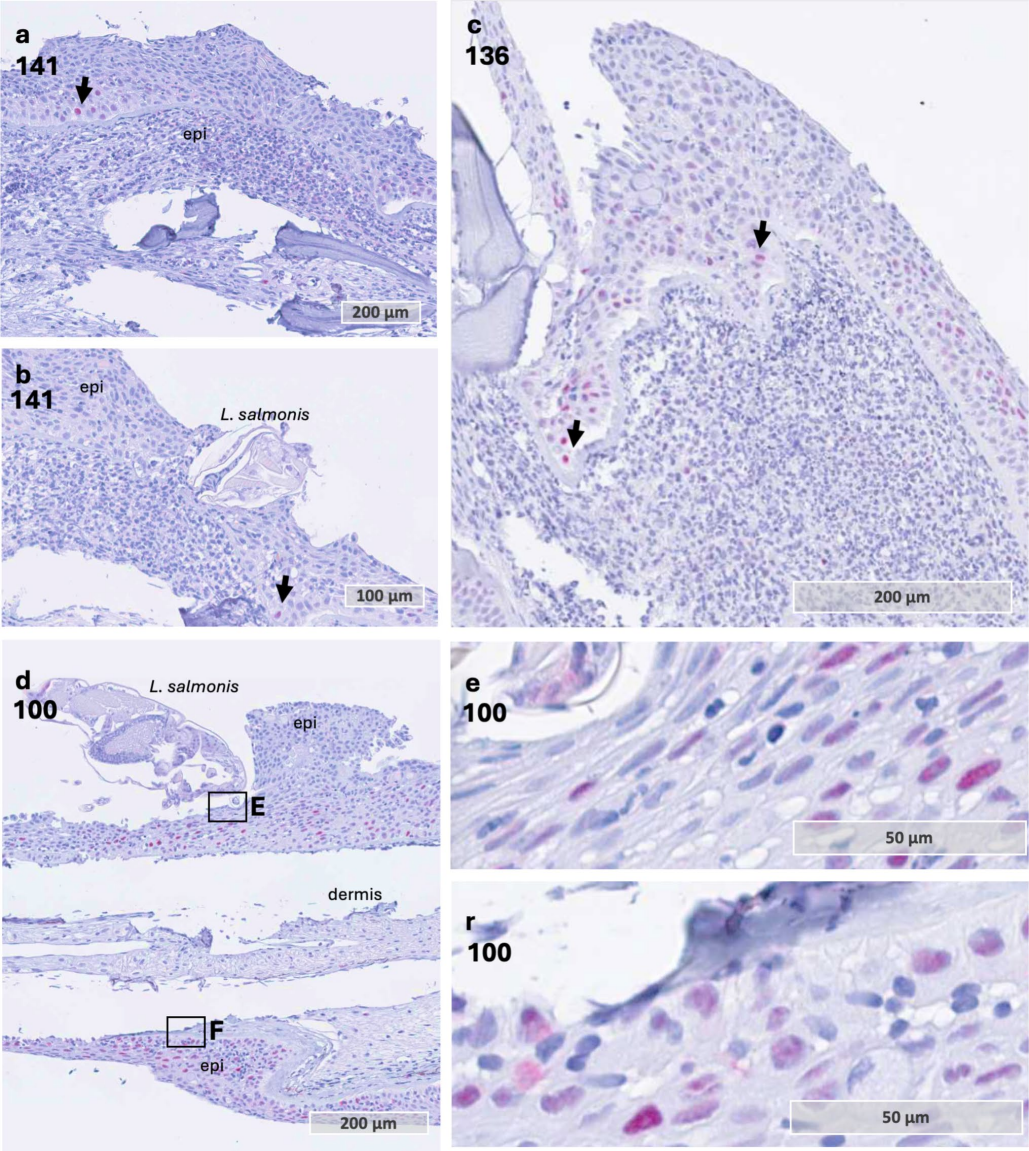
Immunohistochemistry with anti-proliferating nuclear antigen (PCNA). **a** and **b** Serial sections through the lice attachment site of sample 141. Despite thickening of epithelia at the attachment site, only a few cells stained positive for PCNA (arrow) in the epithelium. **c** Several epithelial cells stained positive for PCNA combined with a thickening of the epithelium. Sample 136. **d** Sample with marked host epithelial hyperplasia towards the anterior position of the lice. Sample 100. **e** Positive staining of epithelial cells in the epithelium under the head of the lice. Sample 100. **f** Positive staining of epithelial cells in the epithelium on the opposite side to the attachment site. Sample 100. The magnified areas of **e** and **f** are marked by a rectangle in **d**. Abbreviations: epi epithelium. PCNA + positive staining marked by red color

One particularly interesting sample captured the head *of L. salmonis* embedded in the coho salmon epithelium. Across multiple parallel sections, alterations were observed in both epithelial and dermal tissues, including the basement membrane (Fig. 5 a and b). The epithelial tissue exhibited thickening (hyperplasia) both in front (Fig. 5 a) of and at the host-parasite contact zone (Fig. 5 b). Additionally, the basement membrane appeared thinned with a red stain at the contact zone, whereas it retained a normal appearance, thicker with yellow staining, at increasing distances from the contact point. The red staining of the basement membrane may indicate fibrinoid necrosis (Movat 1955), a form of tissue necrosis characterized by fibrin-like protein deposition, often associated with immune complex-mediated diseases and inflammatory responses (Bajema & Bruijn 2000; Movat 1955). Furthermore, inflammatory cells were present beneath the louse head in the dermal compartment, and in the epithelium near *L. salmonis* (Fig. 5 c, d). Additionally, inflammatory cells were observed migrating from the dermal to the epidermal side through the basement membrane (Fig. 5 e). Furthermore, inflammatory cells within blood vessels exhibited a rounded morphology with neutrophil-like characteristics (Fig. 5 f, g). However, closer to the host-parasite interface, these cells became irregularly shaped (Fig. 5 h), accompanied by amorphous debris. On the opposite side of the attachment site, the basement membrane maintained its normal morphology with red staining, and only a few inflammatory cells were present (Fig. 5 i). Furthermore, few cells stained positive with PCNA on sample 141 (Fig. 6 a and b). This may indicate that the increase in epithelial cells at the host-parasite interaction zone may have resulted from earlier cell division or migration of cells from surrounding tissue. Additionally, positive PCNA staining was observed in two other sample, 100 and 136 (Fig. 6 c and d). Moreover, the staining was not confined to the lice-host interface but appeared to be a general response in the epithelium, with positive cells also observed on the opposite side of the attachment (Fig. 6 d–f). Combined, these observations may suggest that epithelial cell proliferation may be initiated to provide a structural scaffold that facilitates the migration of inflammatory cells, allowing them to come into closer contact with the lice. However, this hypothesis is based on a very limited sample size and therefore warrants further investigation.

### Morphological and inflammatory responses at the *l. salmonis* attachment site in Atlantic salmon

In Atlantic salmon, it was not possible to systematically track the progression of the host response to *L. salmonis* infection, as many samples contained lice from previous challenge trials (Table 5). Nevertheless, several findings are noteworthy in comparison to the host responses observed in coho salmon. First, it was evident that inflammatory cells are recruited to the attachment site in Atlantic salmon; however, in contrast to coho salmon, the number of inflammatory cells was lower, and no inflammatory cells with irregular morphology were observed in Atlantic salmon (Table 5).

**Fig. 7:**
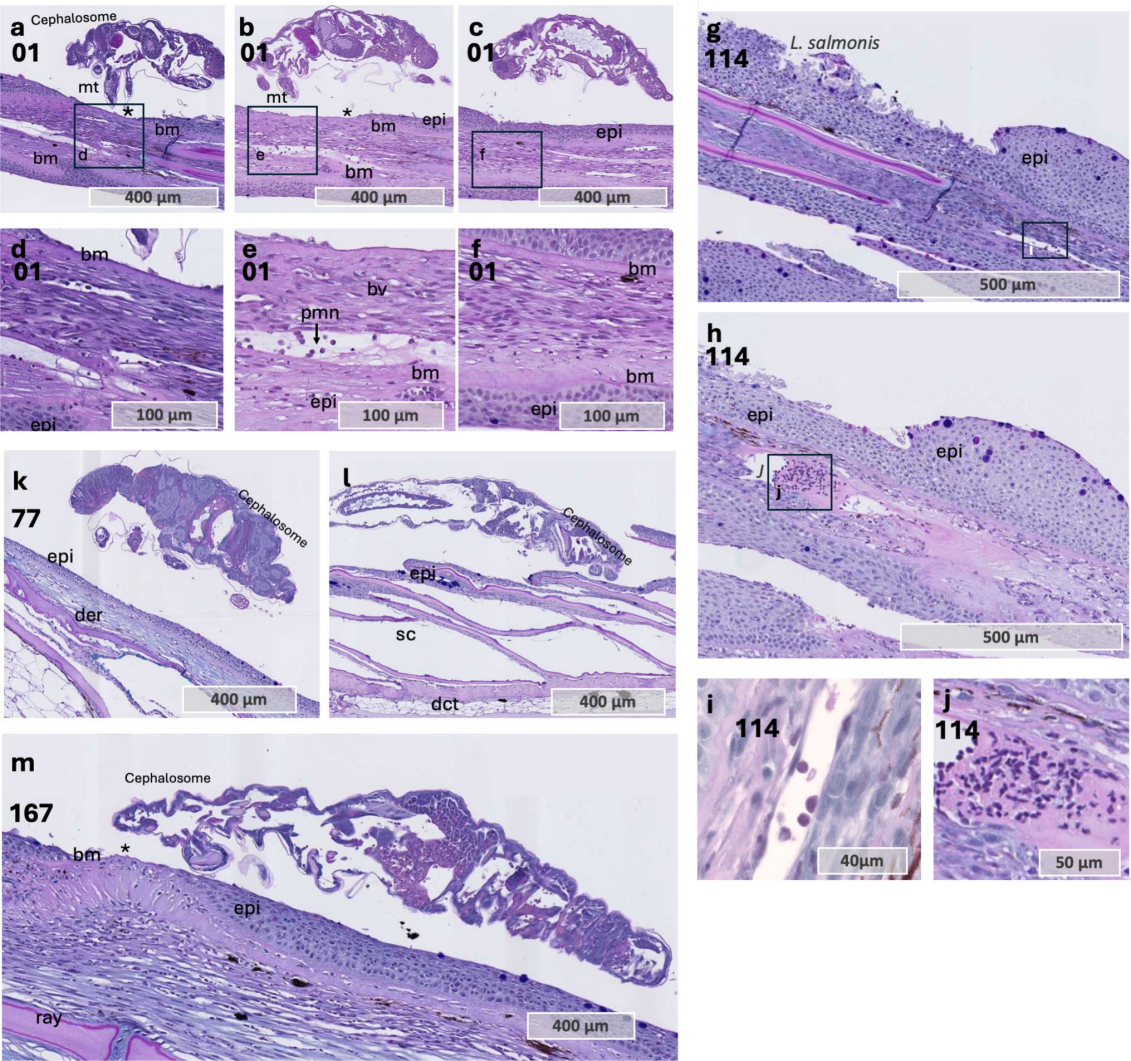
*L. salmonis* attachment site in Atlantic salmon fin and skin. **a**–**c** Sequential sections through the feeding site of a copepodid in Atlantic salmon. Sample 1. **d**–**f** Close-up views of the feeding site, as indicated by the rectangles in panels **a**–**c**. Tissue sections in panel **f** represent areas outside the direct contact with the louse’s mouth tube. **g**–**h** Sequential sections through the feeding site in Atlantic salmon, lice stage unknown. Note epithelial thickening and hyperplasia towards what is likely the position of the head of the louse. Sample 114. **i** Magnified area of the dermal blood vessel with inflammatory cells, area of magnification marked in **g**. Sample 114. **h** Magnified area of the dermal blood vessel with blood cells and amorphous exudate beneath the attachment site (Sample 114). **k**–**l** Three different samples showing the lice attachment site, typically with limited influx of inflammatory cells cells and lack of epithelial hyperplasia. Abbreviations: bm basement membrane, bv blood vessel, * epithelial loss, epi epithelium, mt mouth tube, muc mucous cell, and inf inflammatory cells. Tissue sections were stained with Alcian Blue and Periodic Acid-Schiff

The morphological response at the lice attachment site exhibited considerable variation among samples. In one sample, inflammatory cells were detected within dermal blood vessels beneath the attachment site at 12 hpi near a copepodid attachment site (Fig. 7 a–f). One individual displayed pronounced epithelial hyperplasia (Fig. 7 g–k), whereas minimal changes in skin morphology were noted in other samples (Fig. 7 k–m).

**Fig. 8:**
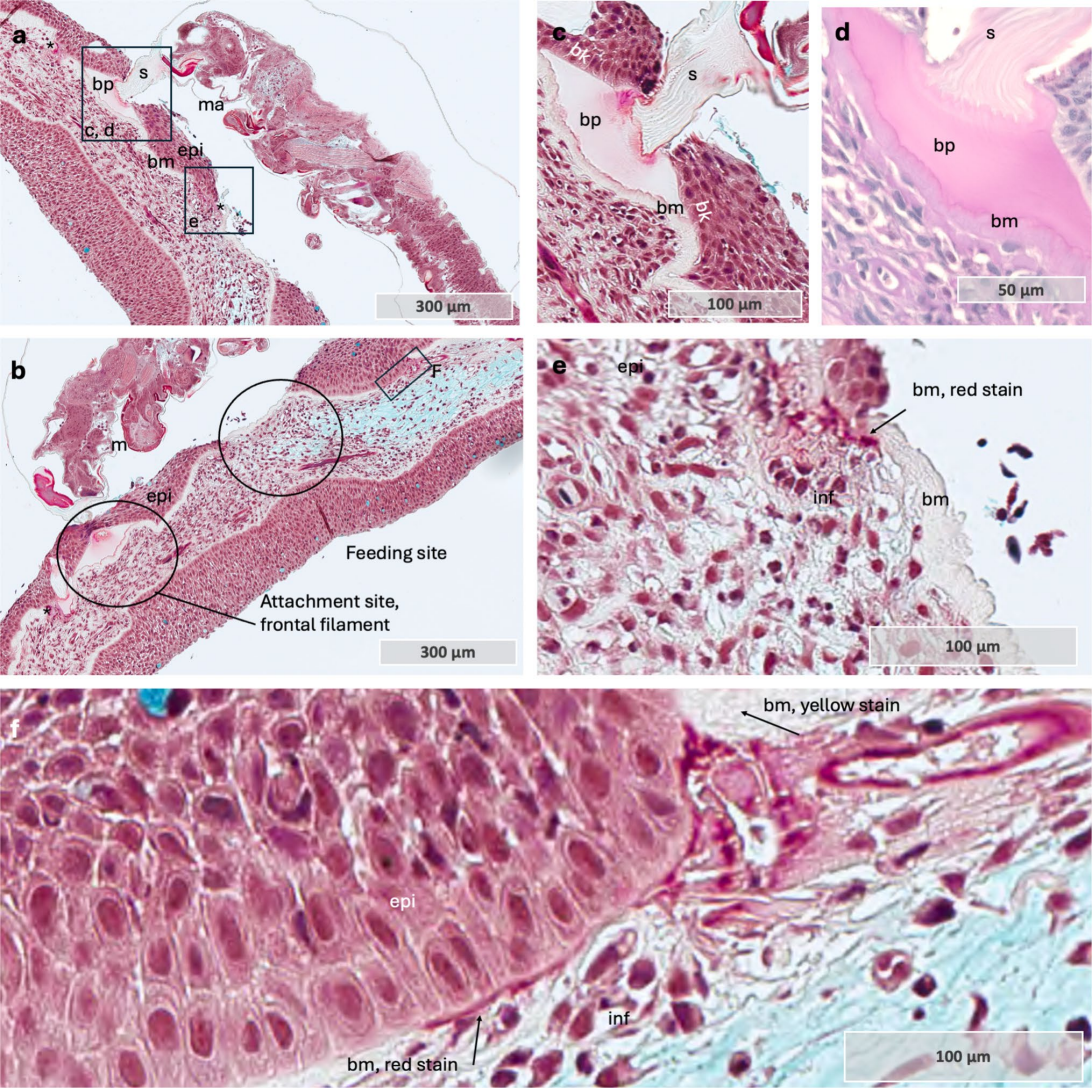
Chalimus attachment site and host responses in sample 41 **a** and **b**. Sequential sections through the attachment site of *L. salmonis*, showing the frontal filament anchored to the host basement membrane and tissue erosion at the feeding site. **c** and **d** Magnified views of the frontal filament from sections **a** and **b**. The basal plate of the frontal filament stains similarly to the host basement membrane with MOVAT and Alcian Blue/PAS staining. Note the intact host basement membrane beneath the basal plate and the normal morphology of basal keratinocytes lining its surface. **e** Area at the feeding site showing alterations in the basement membrane and some inflammatory cells. **f** A region farther from the lice, displaying the most pronounced basement membrane changes and localized aggregates of inflammatory cells. **a**, **b**, **c**, **e**, and **f** stained with MOVAT pentachrome, while **d** stained with Alcian Blue and PAS. Abbreviations: bm basement membrane, bv blood vessel, epi epithelium, bk basal keratinocytes, ma approximate mouth tube area, muc mucous cell, and inf inflammatory cells

In one sample, a sagittal section was captured through the frontal filament of the lice (Fig. 8 a and b). The basal plate at the end of the frontal filament stem stained yellow with MOVAT and pink to purple with Alcian Blue and PAS staining, similar to the staining observed for the host basal membrane (Fig. 8 c and d). Consistent with previous findings (Bron 1993), the basal plate was attached to the basement membrane of the epithelium, and the epithelial cells showed minimal morphological changes, indicating some tolerance to the basal plate material. Hence, it is possible that the basal plate contains components that mimic host proteins to evade the immune system. Furthermore, inflammatory cells were present in the sample, but predominantly at the feeding site (Fig. 8 e and f) and also at some distance from the feeding site and attachment site, approximately 1 mm in distance (Fig. 8g). At these two sites, thinning and red staining of the basement membrane were observed, similar to what was described for sample 141 in coho salmon (Fig. 8). Hence, in Atlantic salmon, inflammatory cells are responding to the lice attachment site, but at the exact host-parasite interphase (basal plate/basal membrane), as previously described (Bron 1993; Gonzalez-Alanis et al. 2001), there is no cellular inflammatory response. Since the lice are firmly attached to the host only at the basal plate zone, it is possible that the immune response by coho salmon may actively damage their basement membrane and epithelial tissue to prevent or weaken the attachment of the frontal basal plate to the basal lamina, thereby enhancing resistance to salmon lice. Although this mechanism was not verified in the present study, it could potentially explain the differences in lice counts and resistance mechanisms observed between the two species.

### Secretory cells of the epithelium at the attachment site

**Fig. 9:**
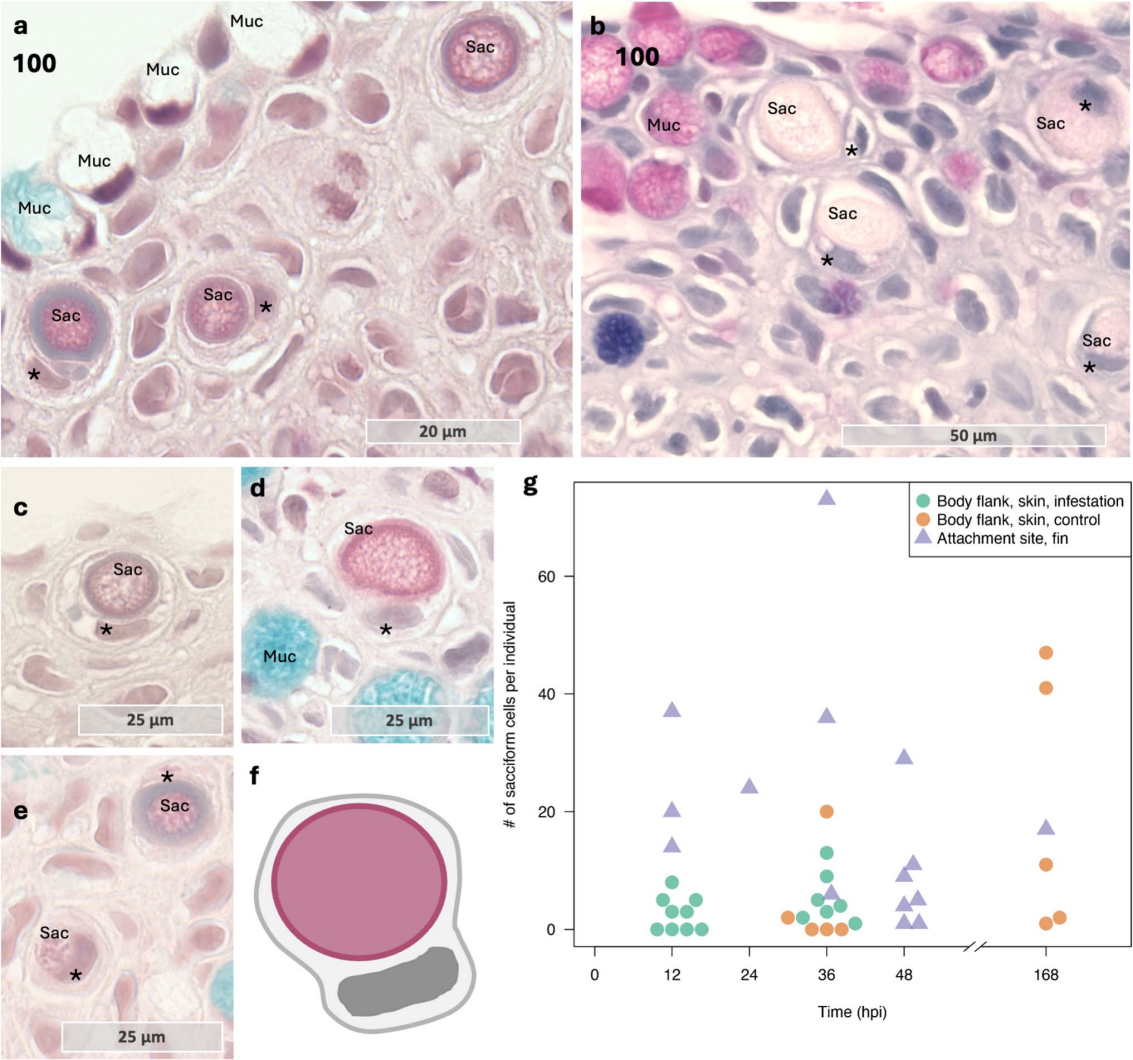
Sacciform cells in the coho salmon epithelium contain sacs (vacuoles) that appear colorless with Alcian Blue and PAS staining and show red coloration with MOVAT Pentachrome stain. **a** Sacciform cells with red staining sacs in the epithelium of coho salmon, together with blue and white staining mucous cells (MOVAT pentachrome). **b** Sacciform cells in the epithelium of coho salmon, the sacs (or vacuoles) are translucent with Alcian Blue and PAS. **c**–**e** Sacciform cells in the epithelium. As the sacciform cells mature, the internal sacs seem to become larger in appearance but maintain a somewhat grainy content. Some sacs have an outer membrane-like enclosure, which is dark red staining in some samples, while other cells have a more grayish outer rim. **f** Schematic presentation of a sacciform cell, showing how the sac is enclosed in a cell body with distally located nuclei. **g** The number of sacciform cells was counted in all samples from coho salmon, body flank control, body flank infested, and attachment site at different time points post-lice infestation. The numbers are raw counts (not normalized to the length of the sample). The nuclei of the sacciform cells are marked with a star. Abbreviations: sac sacciform cell, muc mucous cell

As previous research has noted, there are notable differences in the secretory cells present in the skin of different salmonid species, such as the presence of sacciform cells in coho salmon but not in Atlantic salmon (Johnson & Albright 1992a; Mittal et al. 1981). In this study, sacciform cells were observed in all infested coho salmon samples but were not consistently present in control samples (Fig. 9). While statistical analysis was challenging due to the limited number of samples at each time point, their consistent presence in infested samples implies a potential role in the host response to *L. salmonis* infestation. To the best of our knowledge, the sacciform cell type is not very well described in coho salmon. But by appearance, it is similar to the sacciform cells of arctic char and brown trout. In these species, the sacciform cells are characterized by a single circular vesicle, surrounded by a cell body and a distal located nuclei (Pickering & Fletcher 1987). Given that the coho salmon epithelium respond quickly to infection, deeper characterization of the sacciform cells warrants further investigation.

**Fig. 10:**
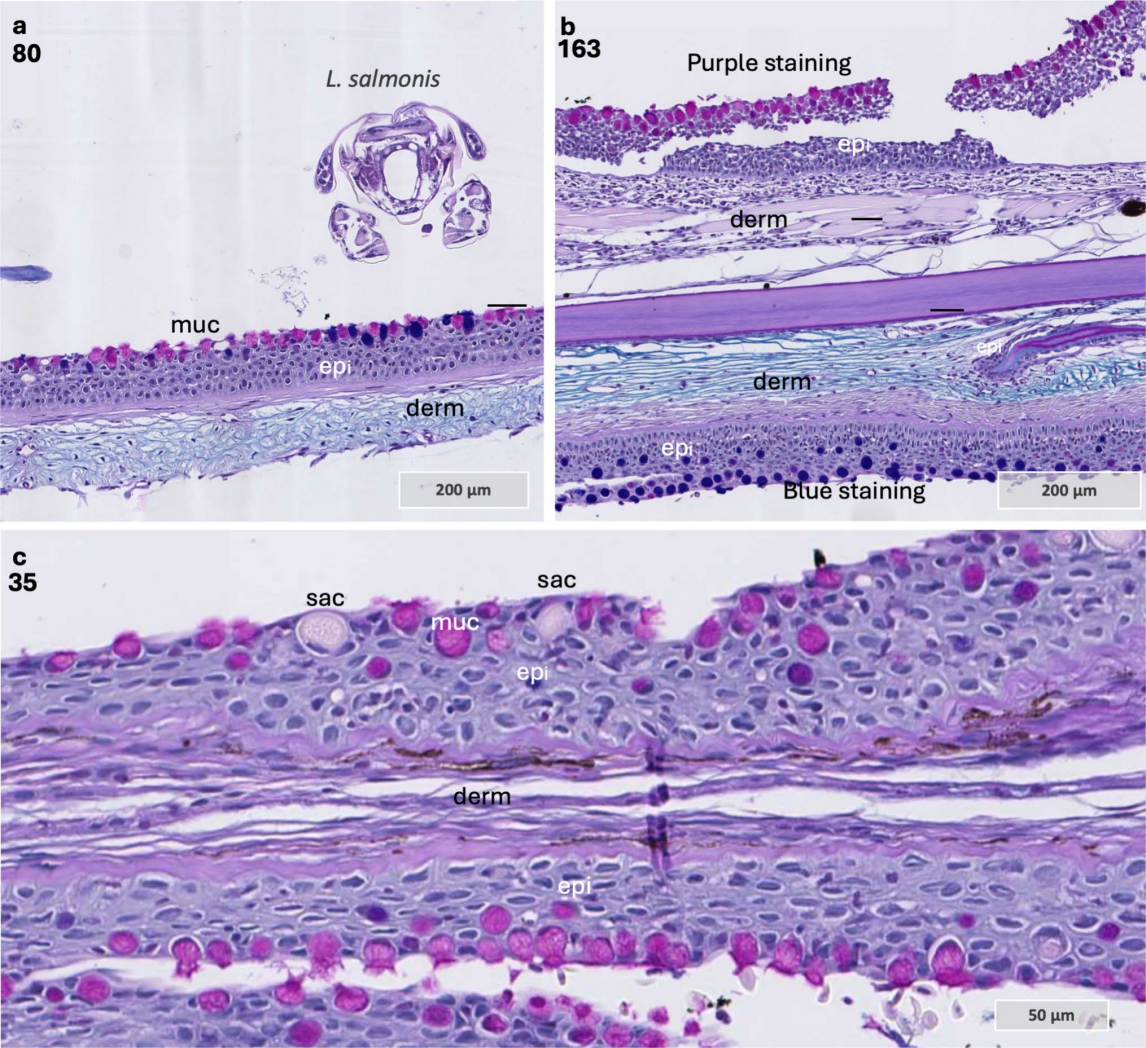
A shift from blue to pink staining mucous cells at the attachment site in Atlantic salmon and coho salmon, with Alcian Blue and PAS staining. **a** A mixture of purple and blue staining mucous cells at the lice attachment site in Atlantic salmon. Sample 80, Atlantic salmon. **b** In this sample, a clear separation of blue and purple staining mucous cells is present on opposite sides of the collected tissue. Sample 163, Atlantic salmon. **c** A coho salmon sample with pink staining mucous cells at the attachment site. Sample 35, coho salmon. Abbreviations: epi epithelium, derm dermis, sac sacciform cell, muc mucous cell

Furthermore, when it comes to mucous cell populations, most samples showed a decrease in mucous cell abundance at the feeding site, consistent with previous studies (Tully & Nolan 2002); however, some exceptions were noted. Two Atlantic salmon samples exhibited an increase in purple-staining mucous cells at the louse attachment site, with similar observations in coho salmon (Fig. 10). In healthy Atlantic salmon skin, purple mucous cells are sparsely distributed (Sveen et al. 2021b), but lice infestation appears to increase their prevalence at the attachment site (Sveen et al. 2021a). The variation in mucous cell color is significant for mucus function, as it indicates a glycosylation shift in mucin proteins (Zaretsky & Wreschner 2013). The transition from blue-staining to purple-staining mucous cells, as observed with Alcian blue and PAS staining, suggests alterations in the glycans on the mucin backbone. These changes could affect mucus properties such as pH, solubility, and its ability to interact with or bind to pathogens, including bacteria (Padra et al. 2014, 2017, 2019). Additionally, several pathogens, including bacteria and parasites, employ a strategy known as “glycan gimmickry,” where they produce glycans that mimic those of the host. This mimicry can facilitate interactions with host lectins, potentially modulating or evading immune responses (de Jong et al. 2022). Therefore, the dynamics of the mucous cell population and mucin glycan patterns could provide insights into mechanisms of host defense evasion and inform the development of novel intervention strategies.

### Neutrophil degranulation at the attachment site in coho salmon

**Fig. 11:**
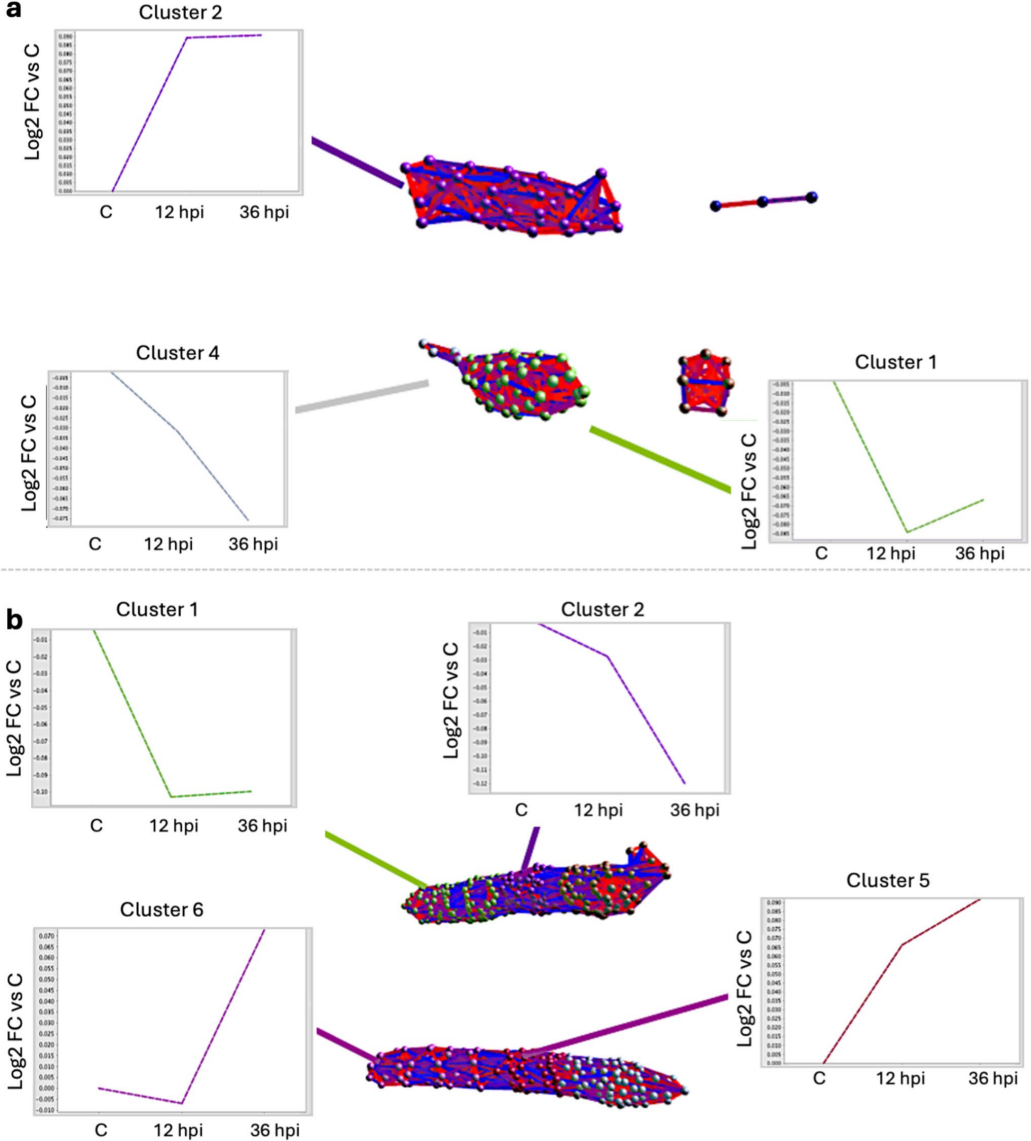
Unbiased clustering of expression profiles reveals time-dependent protein alterations during lice infestation in coho salmon and Atlantic salmon. **a** Expression profile clustering of significantly altered (*p* > 0.05) coho salmon proteins at either 36 hpi/C or both 12 hpi/C and 36 hpi/C. Each sphere (“node”) represents a single protein, and the spatial proximity between nodes (“edges”) indicates similarity in expression profiles. **b** Similar to A, but for Atlantic salmon proteins. For both **a** and **b**, lines highlight examples of relevant cluster profiles over the infectivity time course (C—> 12 hpi—> 36 hpi). Pearson coefficient minimum = 0.70, *R*^2^ = 0.95. The figure was generated in BioLayoutExpress^3D^

**Fig. 12:**
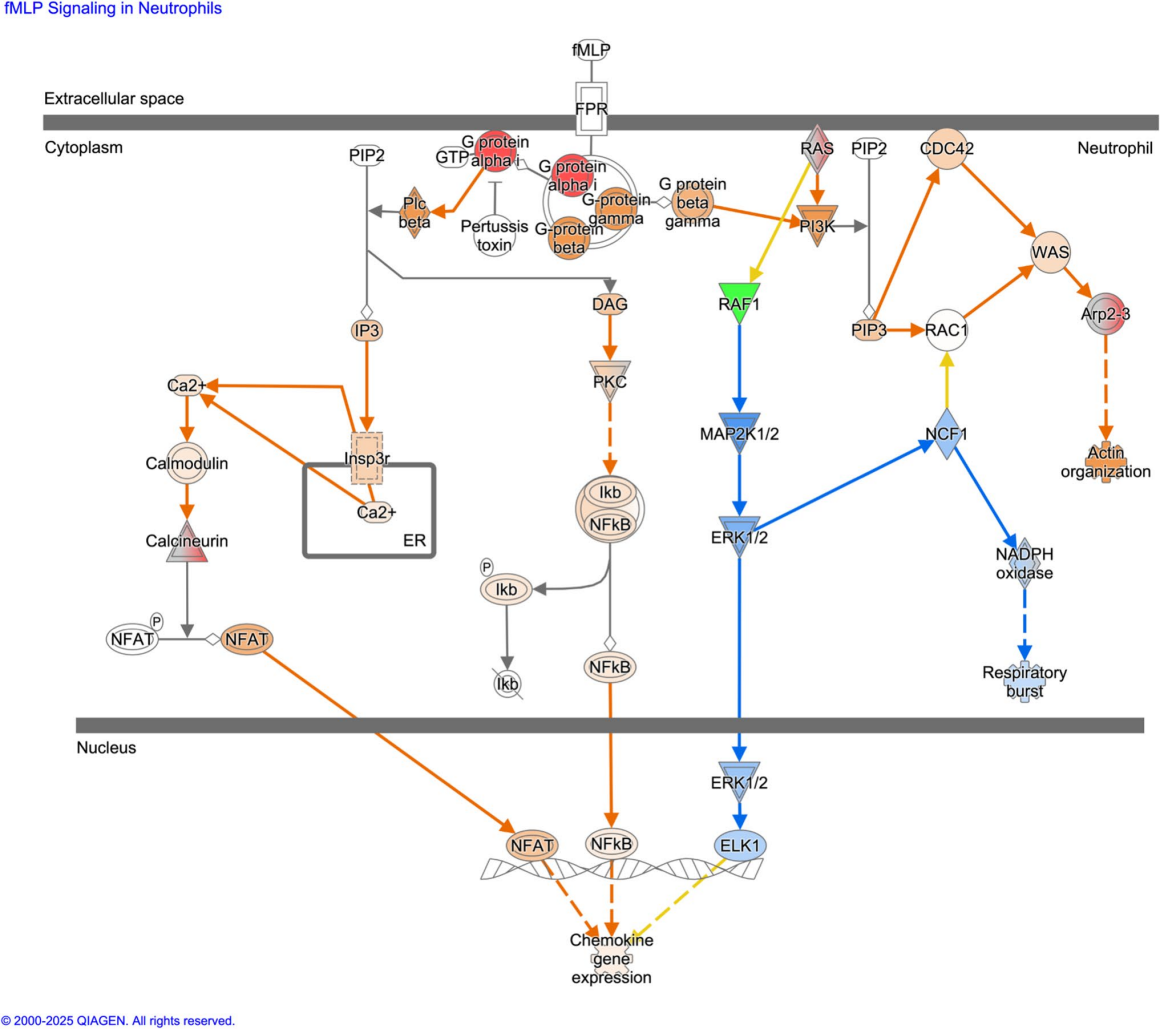
A schematic of fMLP signaling in neutrophils at 36 hpi, a key upregulated pathway in coho salmon during louse infestation. Pathway components were color-coded based on the magnitude of change, with red indicating upregulation from control to 12 hpi and 12 hpi to 36 hpi in coho salmon samples, while green denotes downregulation across the same time points. Additionally, blue and orange indicate *z*-scores, with blue representing inhibition (*z*-score < 2) and orange indicating activation (*z*-score > 2). Gray signifies cases where no activity prediction can be made, while white represents a *z*-score close to zero. The figure was generated using QIAGEN Ingenuity Pathway Analysis

The primary objective of this section was to investigate the proteomic signatures at salmon lice-host interface in coho salmon, building on the histological evidence of degranulation and inflammatory responses observed at the attachment site. This analysis aimed to uncover the molecular mechanisms underlying the structural adaptations and immunomodulatory responses associated with sea lice resistance in coho salmon. Coho salmon skin proteomic analysis identified in total 7719 quantifiable proteins across all timepoints analyzed, with 690 significantly altered at 36 hpi compared to uninfected control samples and 149 showing significant changes across the entire infestation time course. Unbiased expression profile clustering analysis revealed biologically relevant trends, with clusters 1 and 4 containing proteins decreasing in expression over time (Fig. 11). Highlighting proteins associated with the post-translational modification process of neddylation, predicted to be progressively inhibited during infestation (Table 6). Conversely, proteins in the cluster increasing in expression over time (cluster 2) indicated predictive activation of fMLP signaling in neutrophils, linked to an upregulation in chemokine transcription, suggesting a dynamic interplay between suppressed protein modification pathways and enhanced neutrophil-mediated immune signaling during the infestation (Fig. 12). The top upregulated pathways in coho salmon, such as neutrophil degranulation, fMLP signaling in neutrophils, and actin cytoskeleton signaling, indicate a robust acute inflammatory response. Neutrophil degranulation, with the highest -log(*p*-value) of 9.16 (*p* < 0.000000001), suggests that neutrophils play a critical role in the early defense against salmon lice. The release of antimicrobial proteins and enzymes from neutrophils likely contributes to the rejection of copepodids, as evidenced by the influx of neutrophils towards the attachment site by histology. Furthermore, the observed debris at the site of the irregular shaped inflammatory cells, as observed on histology, may represent remnants of tissue degradation due to the inflammatory response (Gierlikowska et al. 2021), or, alternatively, could indicate neutrophil extracellular traps released during immune activation (Palić et al. 2007). Following degranulation, immune cells often undergo apoptosis to limit excessive tissue damage and inflammation (Soehnlein et al. 2008). This apoptotic process may explain why granulocytes were difficult to detect in our previous snRNAseq analysis on lice infested skin (Salisbury et al. 2024). Additionally, pathways like ephrin receptor signaling and integrin signaling, which are involved in cell adhesion and migration, further support the active recruitment and movement of immune cells to the site of infestation. The upregulation of signaling by Rho family GTPases and RAC signaling pathways also points to the dynamic reorganization of the actin cytoskeleton, perhaps facilitating epithelial cell migration.

**Table 6:** Differentially regulated canonical protein pathways in coho salmon during *L. salmonis* infestation. Summary of the top upregulated and downregulated canonical protein pathways in Atlantic salmon during the infestation time course (12 hpi and 36 hpi), ranked by -log(*p*-value). Pathways are categorized by regulation status, with upregulated pathways listed first, followed by downregulated pathways. The ratio column represents the proportion of molecules in the clusters relative to the total molecules in each pathway

Ingenuity canonical pathways	-log (*p*-value)	Ratio (molecules in cluster: molecules in pathway)	Regulation	Cluster
Neutrophil degranulation	9.16	0.0336	Up	2
fMLP signaling in neutrophils	9.04	0.0725	Up	2
Actin cytoskeleton signaling	8.73	0.0484	Up	2
Ephrin receptor signaling	8.51	0.0537	Up	2
Integrin signaling	8.33	0.0516	Up	2
Signaling by rho family GTPases	8.33	0.0446	Up	2
(Hirsch & Cohn 1960) signaling in neurons	7.75	0.0647	Up	2
RAC signaling	7.73	0.0643	Up	2
Epithelial adherens junction signaling	7.2	0.0559	Up	2
RHOGDI signaling	6.99	0.0442	Up	2
KEAP1-NFE2L2 pathway	5.15	0.0549	Down	1 and 4
Neddylation	5.11	0.0285	Down	1 and 4
Mitotic metaphase and anaphase	4.18	0.0255	Down	1 and 4
PTEN regulation	4.1	0.0333	Down	1 and 4
Deubiquitination	3.92	0.0228	Down	1 and 4
Regulation of mitotic cell cycle	3.89	0.0455	Down	1 and 4
Degradation of beta-catenin by the destruction complex	3.81	0.0435	Down	1 and 4
ABC-family proteins mediated transport	3.65	0.0396	Down	1 and 4
Mitotic prophase	3.62	0.0388	Down	1 and 4
Keratinization	3.38	0.0234	Down	1 and 4

In response to *L. salmonis* infestation, Atlantic salmon exhibit upregulation of pathways such as integrin signaling and epithelial adherens junction signaling (Table 7), which are crucial for cell adhesion and migration (Lee & Streuli 2014; Peterson et al. 2024). The simultaneous upregulation of mitochondrial dysfunction and neurodegenerative signaling pathways indicates changes on a cellular level (Kathiresan et al. 2024). Reelin is a large extracellular matrix glycoprotein that plays a crucial role in regulating cell adhesion, migration, and positioning (Santana & Marzolo 2017). However, the exact reason for increased presence of Reelin in the skin during *L. salmonis* infestation is unclear. This upregulation may help reinforce the skin’s structural integrity and facilitate the recruitment of immune cells to the site of infestation, or the lice might modulate reelin signaling to create a more favorable environment for their attachment and feeding (Ranaivoson et al. 2016). The downregulation of MHC class I antigen processing in Atlantic salmon during *L. salmonis* infestation suggests that the lice may exert immunomodulatory effects to evade the host’s immune response (Braden et al. 2020; Tully & Nolan 2002; Umasuthan et al. 2020). It is plausible that *L. salmonis* not only exerts immunosuppressive effects but also modulates adhesion pathways to facilitate its attachment and feeding. Future studies are encouraged to explore these pathways, as they may be essential for developing targeted interventions to improve the resistance of Atlantic salmon to salmon lice.

**Table 7:** Differentially regulated canonical protein pathways in Atlantic salmon during *L. salmonis* infestation. Summary of the top upregulated and downregulated canonical protein pathways in Atlantic salmon during the infestation time course (12 hpi and 36 hpi), ranked by -log(*p*-value). Pathways are categorized by regulation status, with upregulated pathways listed first, followed by downregulated pathways. The ratio column represents the proportion of molecules in the clusters relative to the total molecules in each pathway

Ingenuity canonical pathways	-log(*p*-value)	Ratio (molecules in clusters: molecules in pathway)	Regulation	Clusters
Integrin signaling	5.92	0.0425	Up	1 and 2
Mitochondrial dysfunction	5.9	0.032	Up	1 and 2
Huntington’s disease signaling	5.78	0.0352	Up	1 and 2
Clathrin-mediated endocytosis signaling	5	0.0385	Up	1 and 2
Epithelial adherens junction signaling	4.83	0.0443	Up	1 and 2
Sertoli cell-germ cell junction signaling pathway (enhanced)	4.6	0.0339	Up	1 and 2
BAG2 signaling pathway	4.17	0.0588	Up	1 and 2
Reelin signaling in neurons	4.17	0.0435	Up	1 and 2
nNOS signaling in neurons	4.05	0.0851	Up	1 and 2
C-type lectin receptors (CLRs)	4.05	0.0414	Up	1 and 2
COPI-mediated anterograde transport	12.4	0.167	Down	5 and 6
Protein sorting signaling pathway	11.2	0.112	Down	5 and 6
Intra-Golgi and retrograde Golgi-to-ER traffic	11.1	0.103	Down	5 and 6
Mitotic metaphase and anaphase	9.04	0.0851	Down	5 and 6
Neddylation	7.92	0.0772	Down	5 and 6
Mitotic G2-G2/M phases	7.8	0.0854	Down	5 and 6
RHO GTPase cycle	7.33	0.0556	Down	5 and 6
PRPP biosynthesis I	7.24	1	Down	5 and 6
Class I MHC mediated antigen processing and presentation	6.92	0.0587	Down	5 and 6
COPII-mediated vesicle transport	6.78	0.139	Down	5 and 6

### Spatial transcriptional responses in the fin at the lice attachment site

**Fig. 13:**
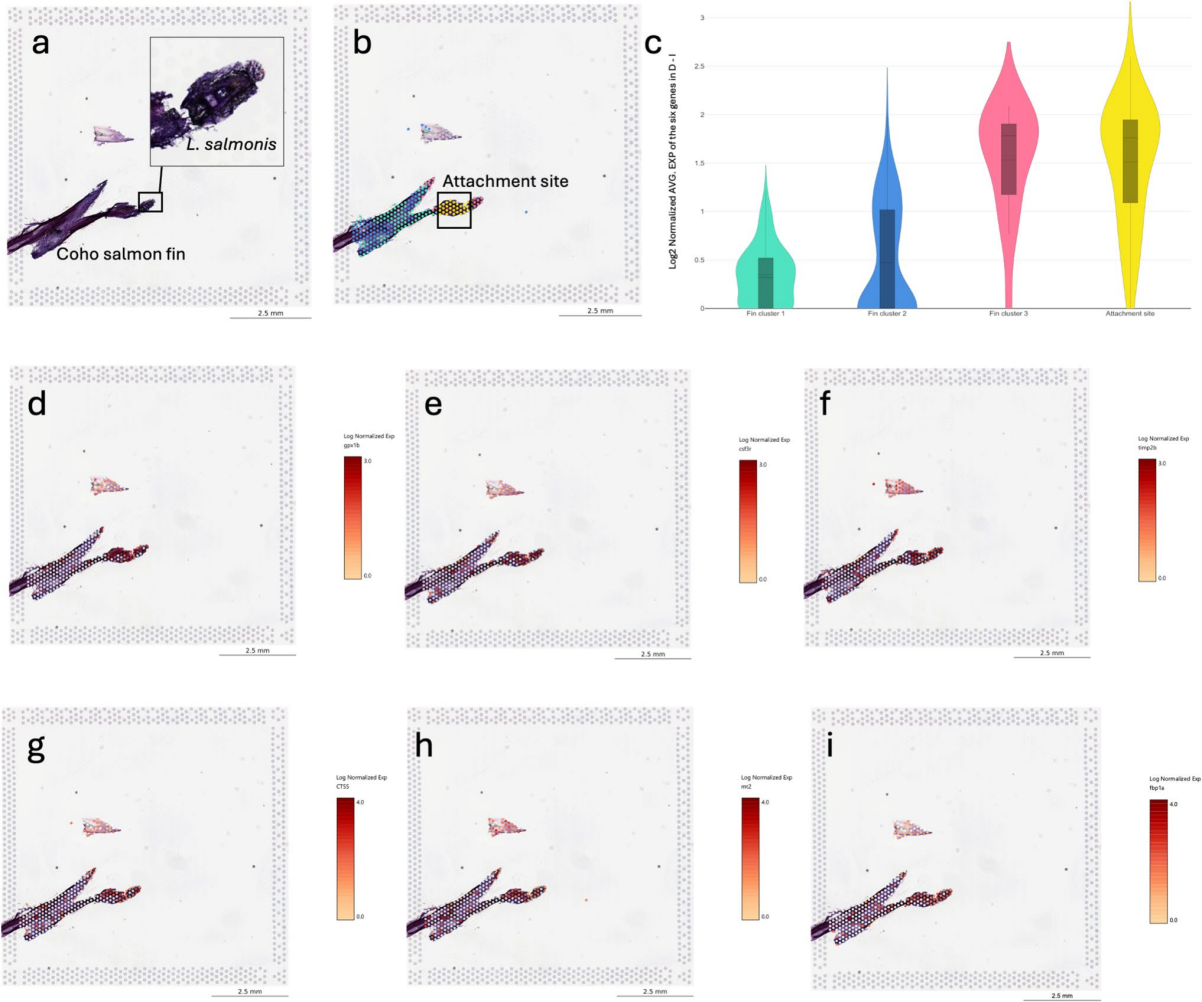
Spatial transcriptomics at the *L. salmonis* attachment site in coho salmon at 12 post-infestation. **a** Spatial transcriptomic expression slide with a coho salmon fin and salmon lice. The area containing lice is marked by a square and a magnified view shown in the upper right corner. **b** Four clusters were assigned to the slide, using K-mean function in loupe browser, each of the cluster is represented by a different color. **c** Log-normalized expression of the six genes listed in Table 8 across the four clusters displayed in **b**. **d** Spatial expression of *gpx1b*. **e** Spatial expression of *csf3r*. **f** Spatial expression of *timp2b*. **g** Spatial expression of *ctss*. **h** Spatial expression of *mt2*. **i** Spatial expression of *fbp1a*. The gene names and scale of expression are provided in the plot, gene IDs in Table 1. The figures were generated in LoupeBrowser

Lastly, to characterize the host responses to *L. salmonis*, we employed spatial transcriptomics on Atlantic salmon and coho salmon fins with *L. salmonis* attached during the initial hours after infestation. Due to the technical challenges of pinpointing the exact attachment point between the host tissue and the small *L. salmonis* copepodid (less than 1 mm in size at the copepodid stage), we were only able to detect tissue from both *L. salmonis* and salmon on one slide. Additionally, since differentially expressed genes (DEGs) are determined within each tissue slide rather than across multiple samples, we focused our analysis on the specimen containing both louse and host tissue. The tissue sample (consisting of both *L. salmonis* and coho salmon tissue) covered 250 barcoded spots. For coho salmon, a total of 18,218 genes were detected, with a median of 1252 genes per spot (supplementary file 1). Manual curation for DEGs at the attachment site, using k-means clustering (*k* = 4), identified six genes with high specificity to the attachment site. These genes showed high specificity at the host-parasite interface (Table 8 and Fig. 13). Granulocyte colony-stimulating factor receptor (*csf3r*), cathepsins (*ctss*), and metalloproteinase inhibitor 2-like (*timp2*) are involved in immune responses, proteolysis, and the regulation of protease activity. *Csf3r* (ENSOKIG00005018820) has been found to be highly expressed in both neutrophils and macrophages (Salisbury et al. 2024). A different paralogue of *ctss* than that detected here (i.e., ENSOKIG00005022279) is also a known marker of macrophages in coho salmon (Salisbury et al. 2024). The upregulation of these genes at the site of louse attachment detected here therefore further support the infiltration of neutrophils at the point of attachment. Additionally, glutathione peroxidase 1b (*gpx1b*) and metallothionein (*mt2*) suggest active antioxidant defenses at the attachment site, providing protection against reactive oxygen species. Overall, the data suggest a localized response directed towards the attachment site.

**Table 8:** Differentially expressed genes at *L. salmonis* attachment site in coho salmon. *p*-value for the comparison of four clusters as presented in Fig. 12 and supplementary file 1

			Fin1		Fin2		Fin3		Attachment site	
Feature ID	Gene Name	Symbol	Log2 FC	*p*-Value	Log2 FC	*p*-Value	Log2 FC	*p*-Value	Log2 FC	*p*-Value
ENSOKIG00005014626	*glutathione peroxidase 1b*	gpx1b	− 4.31	0.00	− 1.99	1.00	1.27	1.00	3.66	0.00
ENSOKIG00005021726	*cathepsin S*	CTSS	− 3.20	0.00	− 1.19	1.00	1.47	0.80	2.85	0.00
ENSOKIG00005018820	*colony stimulating factor 3 receptor*	csf3r	− 2.33	0.00	− 0.75	1.00	1.03	1.00	2.45	0.00
ENSOKIG00005011185	*fructose-1,6-bisphosphatase 1a*	fbp1a	− 2.47	0.00	− 0.71	1.00	1.25	1.00	2.41	0.00
ENSOKIG00005030083	*metallothionein 2*	mt2	− 3.64	0.00	0.18	1.00	2.26	0.06	2.00	0.00
ENSOKIG00005035017	*tissue inhibitor of metalloproteinase 2b*	timp2b	− 2.15	0.00	− 0.52	1.00	1.88	0.17	1.73	0.00

## Concluding remarks

The histological, proteomic, and spatial transcriptomic analyses presented here highlight that a rapid localized immune response occurs in coho salmon following *L. salmonis* attachment. A key feature of this response is the early influx and activation of inflammatory cells, particularly neutrophils, at the infestation site. Histological examination confirms recruitment of inflammatory cells with neutrophil characteristics, while proteomic analysis reveals increased expression of proteins involved in neutrophil degranulation and fMLP signaling, suggesting a robust immune defense. This dynamic interplay between immune cell activation and immune cell recruitment may contribute to the loss or death of the parasite within the first hours and days post-infestation. Further investigation is required to determine whether the immune response in coho salmon directly weakens the anchorage point of *L. salmonis* in the epithelium, leading to its detachment, or whether the immune cells weaken or kill the lice and contribute to its loss from the skin surface.

In contrast, proteomics and histological analysis shows that Atlantic salmon exhibit a suppressed local immune response at the attachment site. Immune cells are recruited to the site of infection under the body of the lice; however, the anchoring point of the parasite to the host, the frontal filament and basal plate, remain unrecognized by the host. Additionally, the findings suggest that differences in mucous cell density and the presence of sacciform cells, absent in Atlantic salmon, may influence the initial success of lice attachment across salmonid species.

Although based on a relatively small sample size, these findings provide compelling evidence for an acute inflammatory response in coho salmon leading to copepodid rejection. However, due to residual lice from the first and second challenge trials in Atlantic salmon, further research is needed to fully capture the complete timeline of infestation in this species. Future studies should focus on detailing the molecular and cellular interactions between the parasite and host during the critical first hours to days post-infestation, with the aim of identifying targeted therapeutics or breeding strategies to enhance salmon lice resistance.

## Supplementary Information

Below is the link to the electronic supplementary material.

Supplementary file1 (XLSX 6579 KB)

Supplementary file2 (PDF 74818 KB)

## Data Availability

Sequence data have been submitted to NCBI&apos;s Sequence Read Archive (SRA) as BioProject PRJNA1149653.

## References

[CR1] Bajema IM, Bruijn JA (2000) What stuff is this! A historical perspective on fibrinoid necrosis. J Pathol 191(3):235–238 10878543 10.1002/(SICI)1096-9896(0000)9999:9999<N/A::AID-PATH610>3.0.CO;2-I

[CR2] Baglama JB, Reichel L (2005) Augmented implicitly restarted Lanczos bidiagonalization algorithms. SIAM J Sci Comput 27(1):19–4

[CR3] Braden LM, Koop BF, Jones SRM (2015) Signatures of resistance to *Lepeophtheirus salmonis* include a TH2-type response at the louse-salmon interface. Dev Comp Immunol 48(1):178–191 25453579 10.1016/j.dci.2014.09.015

[CR4] Braden LM, Michaud D, Groman D, Byrne P, Hori TS, Fast MD (2023) Rejection of Lepeophtheirus salmonis driven in part by chitin sensing is not impacted by seawater acclimitization in Coho salmon (*Oncorhynchus kisutch*). Sci Rep 13(1):9685 37322246 10.1038/s41598-023-36632-0 PMC10272145

[CR5] Braden LM, Monaghan SJ, Fast MD (2020) Salmon immunological defence and interplay with the modulatory capabilities of its ectoparasite *Lepeophtheirus salmonis*. Parasite Immunol 42(8):e12731 32403169 10.1111/pim.12731

[CR6] Bron, J. 1993. A study of the biology and behaviour of the copepodid larva of the salmon louse Lepeophtheirus salmonis (Kroyer, 1837)(Copepoda; Caligidae).

[CR7] Bui S, Halttunen E, Mohn AM, Vågseth T, Oppedal F (2017) Salmon lice evasion, susceptibility, retention, and development differ amongst host salmonid species. ICES J Mar Sci 75(3):1071–1079

[CR8] de Jong H, Wösten MM, Wennekes T (2022) Sweet impersonators: molecular mimicry of host glycans by bacteria. Glycobiology 32(1):11–22 34939094 10.1093/glycob/cwab104 PMC8881735

[CR9] Difford GF, Haugen JE, Aslam ML, Johansen LH, Breiland MW, Hillestad B, Baranski M, Boison S, Moghadam H, Jacq C (2022) Variation in volatile organic compounds in Atlantic salmon mucus is associated with resistance to salmon lice infection. Sci Rep 12(1):4839 35318390 10.1038/s41598-022-08872-z PMC8940922

[CR10] Donovan TA, Moore FM, Bertram CA, Luong R, Bolfa P, Klopfleisch R, Tvedten H, Salas EN, Whitley DB, Aubreville M (2021) Mitotic figures—normal, atypical, and imposters: a guide to identification. Vet Pathol 58(2):243–257 33371818 10.1177/0300985820980049

[CR11] Esteban M (2012) An overview of the immunological defenses in fish skin. ISRN Immunology 2012:29

[CR12] Fast M, Sims DE, Burka JF, Mustafa A, Ross N (2002a) Skin morphology and humoral non-specific defence parameters of mucus and plasma in rainbow trout, coho and Atlantic salmon. Comp Biochem Physiol a: Mol Integr Physiol 132(3):645–657 12044774 10.1016/s1095-6433(02)00109-5

[CR13] Fast MD (2014) Fish immune responses to parasitic copepod (namely sea lice) infection. Dev Comp Immunol 43(2):300–312 24001580 10.1016/j.dci.2013.08.019

[CR14] Fast MD, Muise DM, Easy RE, Ross NW, Johnson SC (2006) The effects of *Lepeophtheirus salmonis* infections on the stress response and immunological status of Atlantic salmon (*Salmo salar*). Fish Shellfish Immunol 21(3):228–241 16483797 10.1016/j.fsi.2005.11.010

[CR15] Fast MD, Ross NW, Mustafa A, Sims DE, Johnson SC, Conboy GA, Speare DJ, Johnson G, Burka JF (2002b) Susceptibility of rainbow trout (*Oncorhynchus mykiss)*, Atlantic salmon (*Salmo salar)* and coho salmon (*Oncorhynchus kisutch*) to experimental infection with sea lice (*Lepeophtheirus salmonis*). Dis Aquat Org 52(1):57–68 10.3354/dao052057 12517006

[CR16] Gierlikowska B, Stachura A, Gierlikowski W, Demkow U (2021) Phagocytosis, degranulation and extracellular traps release by neutrophils—the current knowledge, pharmacological modulation and future prospects. Front Pharmacol 12:666732 34017259 10.3389/fphar.2021.666732 PMC8129565

[CR17] Gonzalez-Alanis P, Wright GM, Johnson SC, Burka JF (2001) Frontal filament morphogenesis in the salmon louse *Lepeophtheirus salmonis*. J Parasitol 87(3):561–574 11426719 10.1645/0022-3395(2001)087[0561:FFMITS]2.0.CO;2

[CR18] Hamre LA, Eichner C, Caipang CMA, Dalvin ST, Bron JE, Nilsen F, Boxshall G, Skern-Mauritzen R (2013) The salmon louse *Lepeophtheirus salmonis* (Copepoda: *Caligidae*) life cycle has only two chalimus stages. PLoS ONE 8(9):e73539 24069203 10.1371/journal.pone.0073539 PMC3772071

[CR19] Hirsch JG, Cohn ZA (1960) Degranulation of polymorphonuclear leucocytes following phagocytosis of microorganisms. J Exp Med 112(6):1005 13714579 10.1084/jem.112.6.1005 PMC2137318

[CR20] Johnson S, Albright L (1992a) Comparative susceptibility and histopathology of the response of naive Atlantic, chinook and coho salmon to experimental infection with *Lepeophtheirus salmonis*. Dis Aquat Org 14:179–193

[CR21] Johnson S, Albright L (1992b) Effects of cortisol implants on the susceptibility and the histopathology of the responses of naive coho salmon *Oncorhynchus kisutc*h to experimental infection with Lepeophtheirus salmonis. Caligidae, Copepoda

[CR22] Johnson SC, Albright LJ (1991) Development, growth, and survival of *Lepeophtheirus Salmoni*s (Copepoda: Caligidae) under laboratory conditions. J Mar Biol Assoc UK 71(2):425–436

[CR23] Jones SR, Fast MD, Johnson SC, Groman DB (2007) Differential rejection of salmon lice by pink and chum salmon: disease consequences and expression of proinflammatory genes. Dis Aquat Organ 75(3):229–238 17629118 10.3354/dao075229

[CR24] Kabata Z (1974) Mouth and mode of feeding of Caligidae (Copepoda), parasites of fishes, as determined by light and scanning electron microscopy. Journal of the Fisheries Board of Canada 31(10):1583–1588

[CR25] Kathiresan, D.S., Balasubramani, R., Marudhachalam, K., Jaiswal, P., Ramesh, N., Sureshbabu, S.G., Puthamohan, V.M., Vijayan, M. 2024. Role of mitochondrial dysfunctions in neurodegenerative disorders: advances in mitochondrial biology. *Molecular Neurobiology*, 1–29. 10.1007/s12035-024-04469-x 39269547

[CR26] Kaur K, Okubamichael MA, Eide SH, Pittman K (2024) Impact of krill meal on enhancing skin mucosal health and reducing sea lice in Atlantic salmon. Journal of Marine Science and Engineering 12(9):1486

[CR27] Kolberg L, Raudvere U, Kuzmin I, Adler P, Vilo J, Peterson H (2023) g: profiler—interoperable web service for functional enrichment analysis and gene identifier mapping (2023 update). Nucleic Acids Res 51(W1):W207–W212 37144459 10.1093/nar/gkad347 PMC10320099

[CR28] Krasnov A, Skugor S, Todorcevic M, Glover KA, Nilsen F (2012) Gene expression in Atlantic salmon skin in response to infection with the parasitic copepod *Lepeophtheirus salmonis*, cortisol implant, and their combination. BMC Genomics 13(1):130 22480234 10.1186/1471-2164-13-130 PMC3338085

[CR29] Lee JL, Streuli CH (2014) Integrins and epithelial cell polarity. J Cell Sci 127(15):3217–3225 24994933 10.1242/jcs.146142 PMC4117227

[CR30] Midtbø HMD, Eichner C, Hamre LA, Dondrup M, Flesland L, Tysseland KH, Kongshaug H, Borchel A, Skoge RH, Nilsen F (2024) Salmon louse labial gland enzymes: implications for host settlement and immune modulation. Front Genet 14:1303898 38299097 10.3389/fgene.2023.1303898 PMC10828956

[CR31] Mittal AK, Whitear M, Bullock AM (1981) Sacciform cells in the skin of teleost fish. Z Mikrosk Anat Forsch 95(4):559–585 6171112

[CR32] Movat HZ (1955) Demonstration of all connective tissue elements in a single section; pentachrome stains. AMA Arch Pathol 60(3):289–295 13248341

[CR33] Nagasawa K (2001) Annual changes in the population size of the salmon louse Lepeophtheirus salmonis (Copepoda: Caligidae) on high-seas Pacific salmon (Oncorhynchus spp.), and relationship to host abundance. Hydrobiologia 453(1):411–416

[CR34] Øvergård A-C, Eichner C, Nuñez-Ortiz N, Kongshaug H, Borchel A, Dalvin S (2023) Transcriptomic and targeted immune transcript analyses confirm localized skin immune responses in Atlantic salmon towards the salmon louse. Fish Shellfish Immunol 138:108835 37236552 10.1016/j.fsi.2023.108835

[CR35] Øvergård A-C, Midtbø HM, Hamre LA, Dondrup M, Bjerga GE, Larsen Ø, Chettri JK, Buchmann K, Nilsen F, Grotmol S (2022) Small, charged proteins in salmon louse (*Lepeophtheirus salmonis*) secretions modulate Atlantic salmon (*Salmo salar*) immune responses and coagulation. Sci Rep 12(1):7995 35568726 10.1038/s41598-022-11773-w PMC9107468

[CR36] Padra JT, Sundh H, Jin C, Karlsson NG, Sundell K, Lindén SK (2014) Aeromonas salmonicida binds differentially to mucins isolated from skin and intestinal regions of Atlantic salmon in an N-acetylneuraminic acid-dependent manner. Infect Immun 82(12):5235–5245 25287918 10.1128/IAI.01931-14 PMC4249282

[CR37] Padra, J.T., Sundh, H., Sundell, K., Venkatakrishnan, V., Jin, C., Samuelsson, T., Karlsson, N.G., Lindén, S.K. 2017. Aeromonas salmonicida growth in response to atlantic salmon mucins differs between epithelial sites, is governed by sialylated and N-acetylhexosamine-containing O-glycans, and is affected by Ca2+. *Infection and immunity*, **85**(8). 10.1128/IAI.00189-17 PMC5520437 28533470

[CR38] Padra M, Adamczyk B, Flahou B, Erhardsson M, Chahal G, Smet A, Jin C, Thorell A, Ducatelle R, Haesebrouck F (2019) Helicobacter suis infection alters glycosylation and decreases the pathogen growth inhibiting effect and binding avidity of gastric mucins. Mucosal Immunol 12(3):784–794 30846831 10.1038/s41385-019-0154-4

[CR39] Palić D, Ostojić J, Andreasen CB, Roth JA (2007) Fish cast NETs: neutrophil extracellular traps are released from fish neutrophils. Dev Comp Immunol 31(8):805–816 17222907 10.1016/j.dci.2006.11.010

[CR40] Peterson, R.J., Reed, R.C., Zamecnik, C.R., Sallam, M.A., Finbloom, J.A., Martinez, F.J., Levy, J.M., Moonwiriyakit, A., Desai, T.A., Koval, M. 2024. Apical integrins as a switchable target to regulate the epithelial barrier. *Journal of Cell Science*, **137**(24). 10.1242/jcs.263580 PMC11795292 39552289

[CR41] Pickering AD, Fletcher JM (1987) Sacciform cells in the epidermis of the brown trout, Salmo trutta, and the Arctic char. Salvetinus Alpinus Cell and Tissue Research 247(2):259–265 3815480 10.1007/BF00218307

[CR42] Pike, A., Mackenzie, K., Rowand, A. 1993. *Ultrastructure of the frontal filament in chalimus larvae of Caligus elongatus and Lepeophtheirus salmonis from Atlantic salmon, Salmo salar*. Ellis Horwood, Chichester, UK.

[CR43] Pike AW, Wadsworth SL (1999) Sealice on salmonids: their biology and control. Adv Parasitol 44:233–337 10563397 10.1016/s0065-308x(08)60233-x

[CR44] Poley JD, Braden LM, Messmer AM, Igboeli OO, Whyte SK, Macdonald A, Rodriguez J, Gameiro M, Rufener L, Bouvier J (2018) High level efficacy of lufenuron against sea lice (*Lepeophtheirus salmonis*) linked to rapid impact on moulting processes. Int J Parasitol Drugs Drug Resist 8(2):174–188 29627513 10.1016/j.ijpddr.2018.02.007 PMC6039351

[CR45] Ranaivoson FM, Von Daake S, Comoletti D (2016) Structural insights into Reelin function: present and future. Front Cell Neurosci 10:137 27303268 10.3389/fncel.2016.00137 PMC4882317

[CR46] Roberts, R.E., Hallett, M.B. 2019. Neutrophil cell shape change: mechanism and signalling during cell spreading and phagocytosis. *Int J Mol Sci*, **20**(6). 10.3390/ijms20061383 PMC6471475 30893856

[CR47] Salisbury SJ, Daniels RR, Monaghan SJ, Bron JE, Villamayor PR, Gervais O, Fast MD, Sveen L, Houston RD, Robinson N, Robledo D (2024) Keratinocytes drive the epithelial hyperplasia key to sea lice resistance in coho salmon. BMC Biol 22(1):160 39075472 10.1186/s12915-024-01952-8 PMC11287951

[CR48] Santana J, Marzolo MP (2017) The functions of Reelin in membrane trafficking and cytoskeletal dynamics: implications for neuronal migration, polarization and differentiation. Biochem J 474(18):3137–3165 28887403 10.1042/BCJ20160628

[CR49] Skugor S, Glover KA, Nilsen F, Krasnov A (2008) Local and systemic gene expression responses of Atlantic salmon (*Salmo salar* L.) to infection with the salmon louse (*Lepeophtheirus salmonis*). BMC Genomics 9(1):498 18945374 10.1186/1471-2164-9-498 PMC2582245

[CR50] Soehnlein O, Zernecke A, Eriksson EE, Rothfuchs AG, Pham CT, Herwald H, Bidzhekov K, Rottenberg ME, Weber C, Lindbom L (2008) Neutrophil secretion products pave the way for inflammatory monocytes. Blood 112(4):1461–1471 18490516 10.1182/blood-2008-02-139634 PMC3400540

[CR51] Sommerset, I., Nielsn, J.W., Modal, T., Oliveira, V.H.S.D., Svendsen, J., Haukaas, A., Brun, E. 2023. In Norwegain “Fiskehelseraporten 2023”. Norwegian Veterinary Institute

[CR52] Sutherland BJG, Koczka KW, Yasuike M, Jantzen SG, Yazawa R, Koop BF, Jones SRM (2014) Comparative transcriptomics of Atlantic Salmo salar, chum Oncorhynchus keta and pink salmon O. gorbuscha during infections with salmon lice Lepeophtheirus salmonis. BMC Genomics 15(1):200 24628956 10.1186/1471-2164-15-200 PMC4004277

[CR53] Suvarna, K.S., Layton, C., Bancroft, J.D. 2018. *Bancroft’s theory and practice of histological techniques*. Elsevier health sciences.

[CR54] Sveen L, Krasnov A, Timmerhaus G, Bogevik AS (2021) Responses to mineral supplementation and salmon lice (*Lepeophtheirus salmonis*) infestation in skin layers of Atlantic salmon (*Salmo salar* L.). Genes 12(4):602 33921813 10.3390/genes12040602 PMC8073069

[CR55] Sveen, L., Robinson, N., Krasnov, A., Daniels, R.R., Vaadal, M., Karlsen, C., Ytteborg, E., Robledo, D., Salisbury, S., Dagnachew, B., Lazado, C.C., Tengs, T. 2023. Transcriptomic landscape of Atlantic salmon (Salmo salar L.) skin. *G3 Genes|Genomes|Genetics*, **13**(11). 10.1093/g3journal/jkad215 PMC10627282 37724757

[CR56] Sveen L, Timmerhaus G, Johansen L-H, Ytteborg E (2021b) Deep neural network analysis - a paradigm shift for histological examination of health and welfare of farmed fish. Aquaculture 532:736024

[CR57] Sveen L, Timmerhaus G, Krasnov A, Takle H, Handeland S, Ytteborg E (2019) Wound healing in post-smolt Atlantic salmon (*Salmo salar* L.). Scientific reports 9(1):3565 30837496 10.1038/s41598-019-39080-x PMC6400935

[CR58] Sveen L, Timmerhaus G, Krasnov A, Takle H, Stefansson SO, Handeland SO, Ytteborg E (2018) High fish density delays wound healing in Atlantic salmon (*Salmo salar*). Sci Rep 8(1):1–13 30443022 10.1038/s41598-018-35002-5 PMC6237775

[CR59] Tadiso TM, Krasnov A, Skugor S, Afanasyev S, Hordvik I, Nilsen F (2011) Gene expression analyses of immune responses in Atlantic salmon during early stages of infection by salmon louse (*Lepeophtheirus salmonis*) revealed bi-phasic responses coinciding with the copepod-chalimus transition. BMC Genomics 12:141 21385383 10.1186/1471-2164-12-141 PMC3062619

[CR60] Theocharidis A, Van Dongen S, Enright AJ, Freeman TC (2009) Network visualization and analysis of gene expression data using BioLayout Express 3D. Nat Protoc 4(10):1535–1550 19798086 10.1038/nprot.2009.177

[CR61] Tully O, Nolan D (2002) A review of the population biology and host–parasite interactions of the sea louse Lepeophtheirus salmonis (Copepoda: Caligidae). Parasitology 124(7):165–182 10.1017/s0031182002001889 12396223

[CR62] Umasuthan, N., Xue, X., Caballero-Solares, A., Kumar, S., Westcott, J.D., Chen, Z., Fast, M.D., Skugor, S., Nowak, B.F., Taylor, R.G., Rise, M.L. 2020. Transcriptomic profiling in fins of Atlantic salmon parasitized with sea lice: evidence for an early imbalance between chalimus-induced immunomodulation and the host’s defense response. *Int J Mol Sci*, **21**(7). 10.3390/ijms21072417 PMC7177938 32244468

[CR63] Zaretsky, J.Z., Wreschner, D.H. 2013. Mucins – potential regulators of cell functions. Gel-Forming and Soluble Mucins. *Bentham Science Publishers Ltd*, **1**, 639.

