# Supplementary material for: Local inflammation at the salmon louse (Lepeophtheirus salmonis) attachment site contributes to copepodid rejection in coho salmon (Oncorhynchus kisutch)
Source: Cell Tissue Res. 2025 Jun 4;401(2):181–211. doi: 10.1007/s00441-025-03976-0 (PMC12325551; doi:10.1007/s00441-025-03976-0)

This file contains histological images of coho salmon and Atlantic salmon from the *Lepeophtheirus salmonis* attachment site. Additionally, it includes an image of a coho fin and salmon lice on a 10X Genomics expression slide for alignment with spatial transcriptomic sequencing data.

Histological images of coho salmon and Atlantic salmon

Each picture is labeled with species, staining method and sample ID.

Coho salmon, AB / PAS

Coho salmon, IHC

Atlantic salmon, AB / PAS

Color codes for species and staining

Sample ID

Coho salmon_1_33
Fin_Dorsal_12_hpi

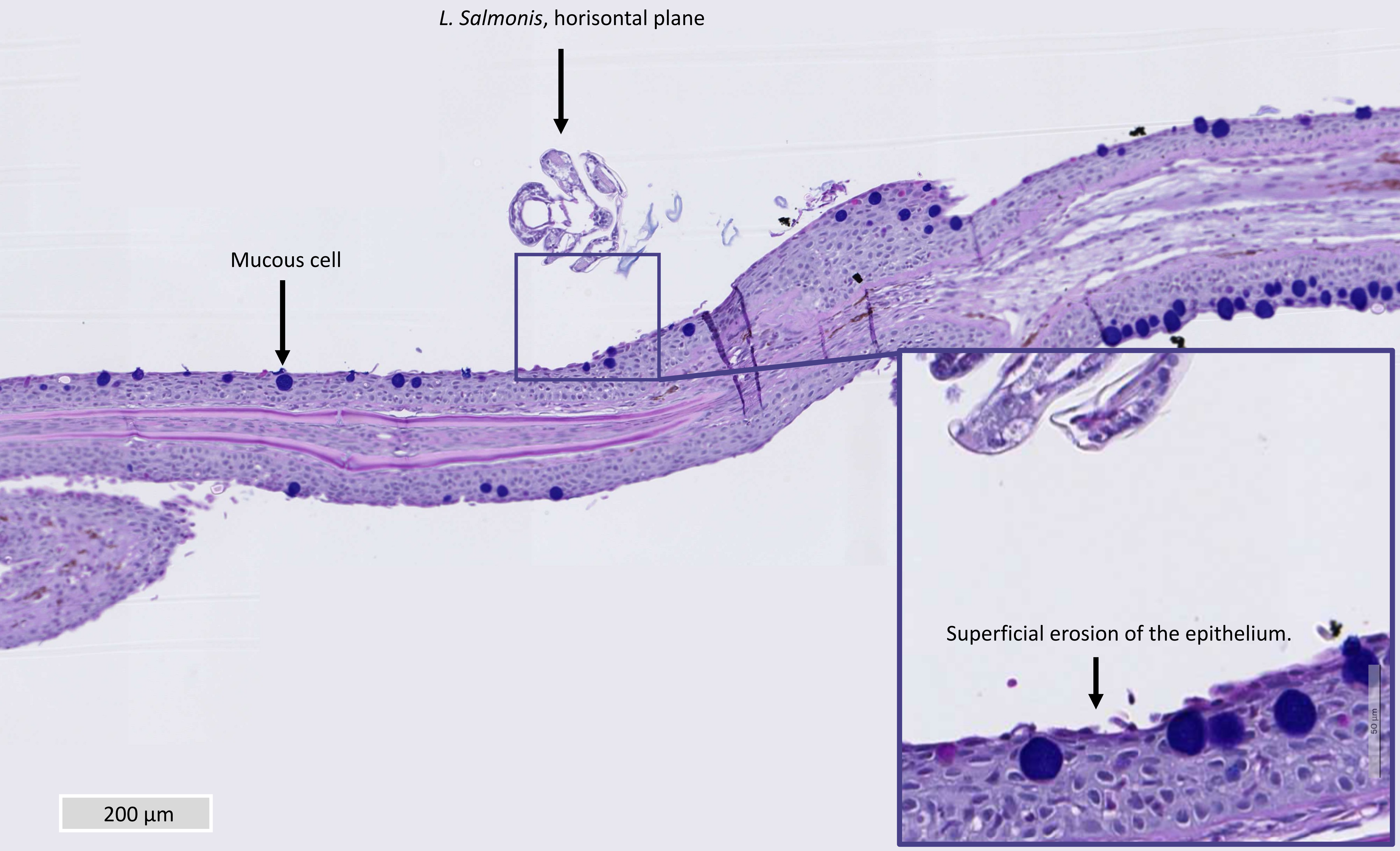

Coho salmon_1_33
Fin_Dorsal_12_hpi

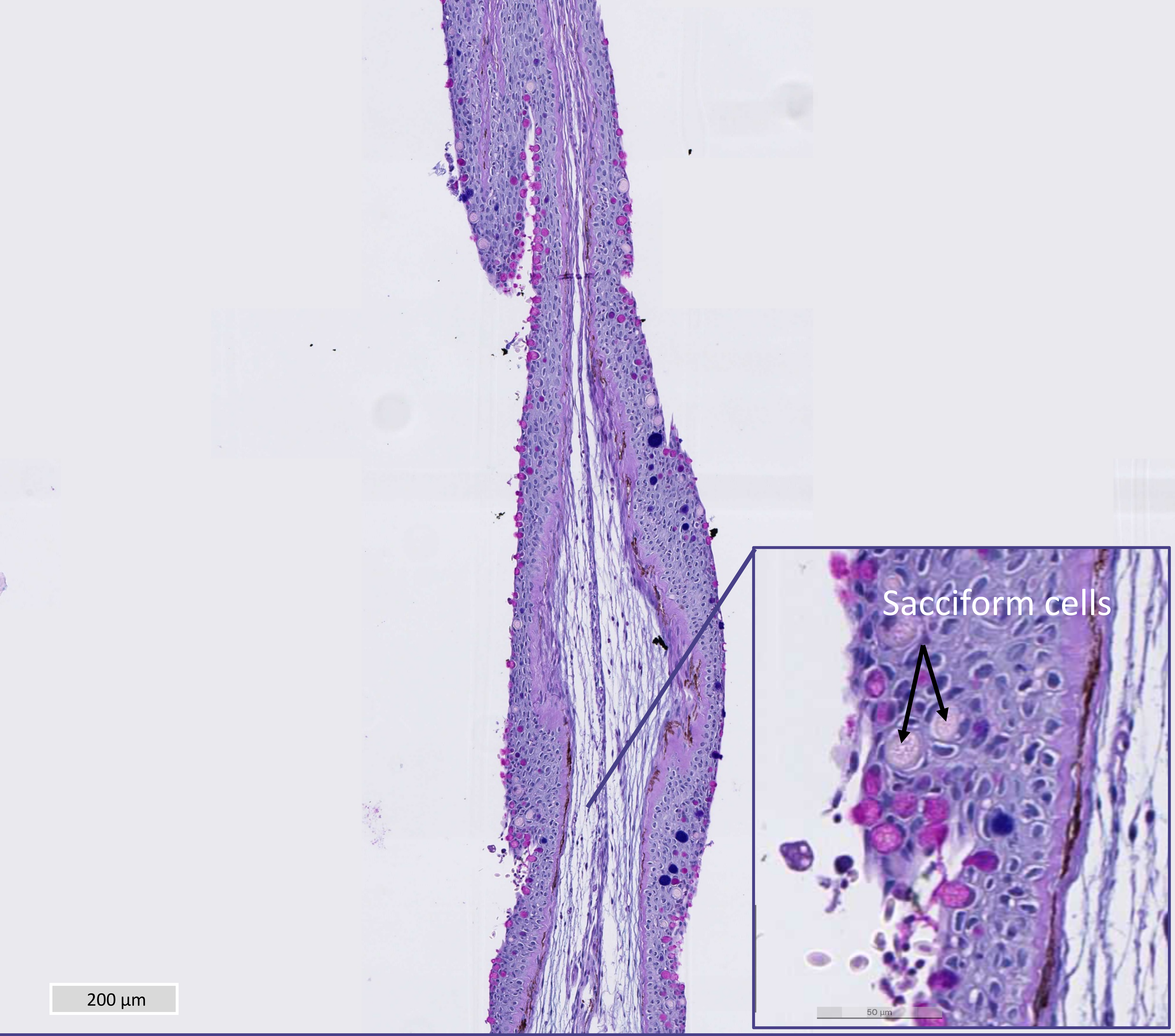

Coho salmon_2_35
Fin_Dorsal_12_hpi

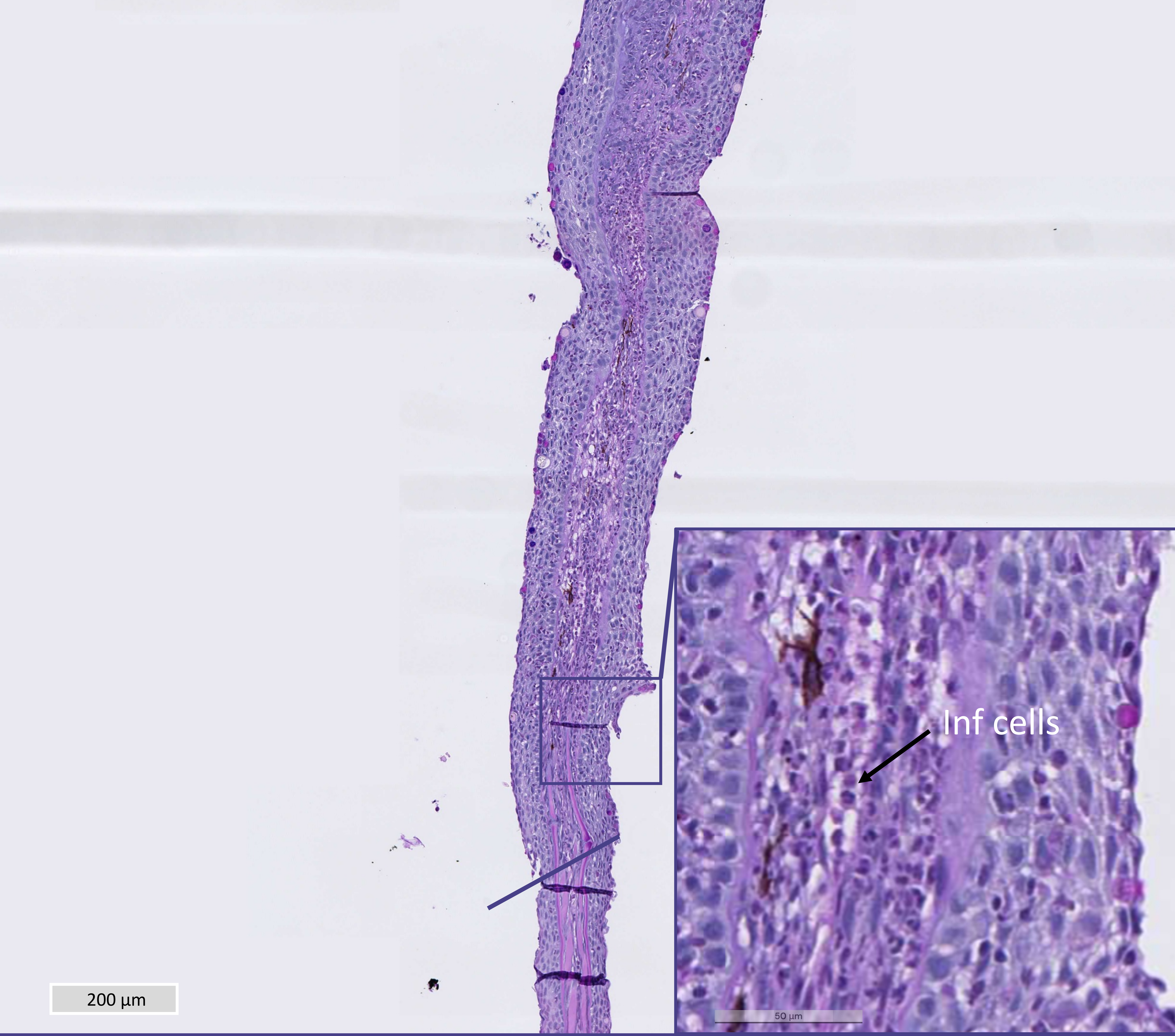

Coho salmon_3_36
Fin_PC_12_hpi

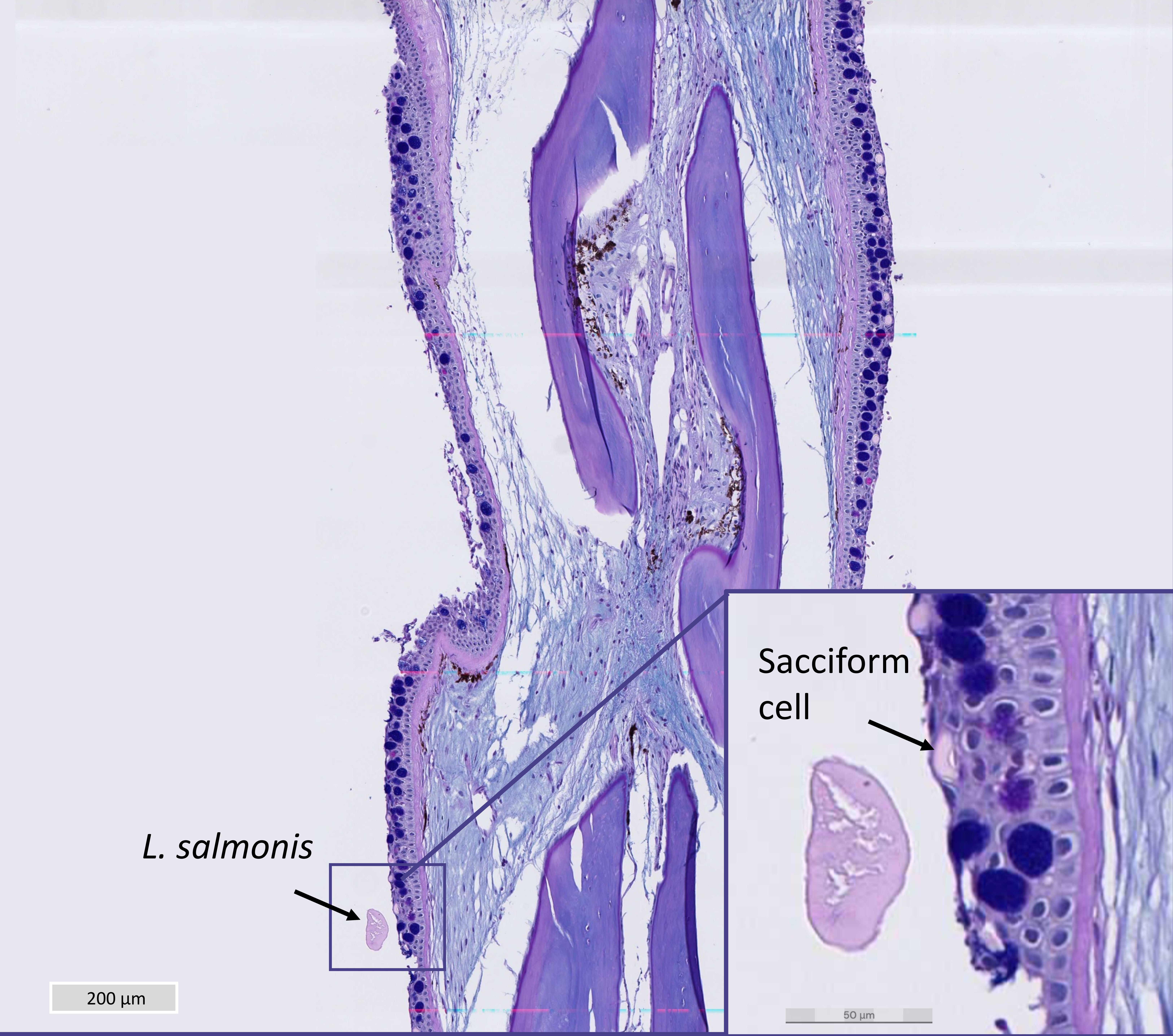

Coho salmon_4_66
Fin_Caudal_24_hpi

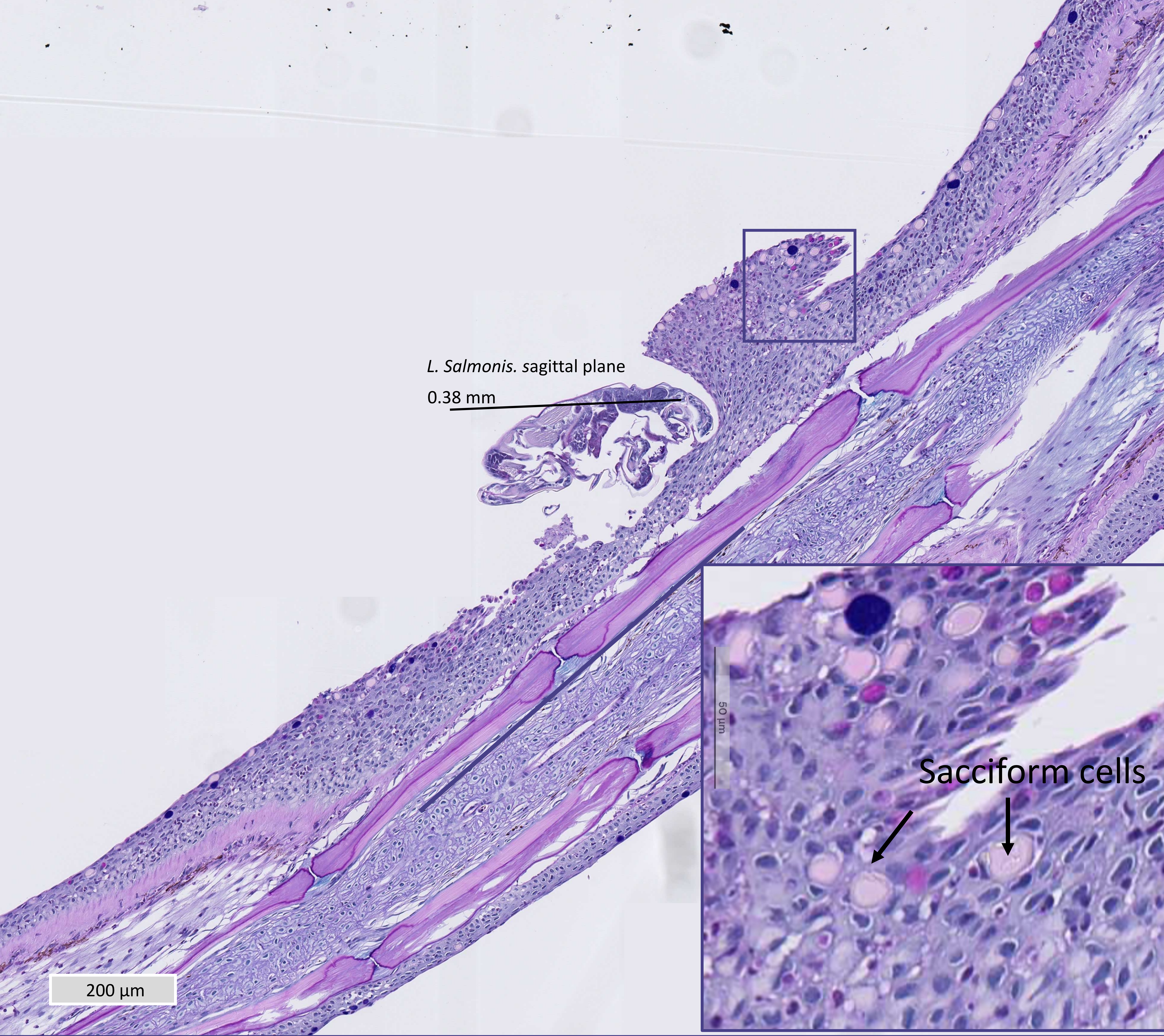

Coho salmon_5_100

Fin Caudal 36 hpi

Section 1_3

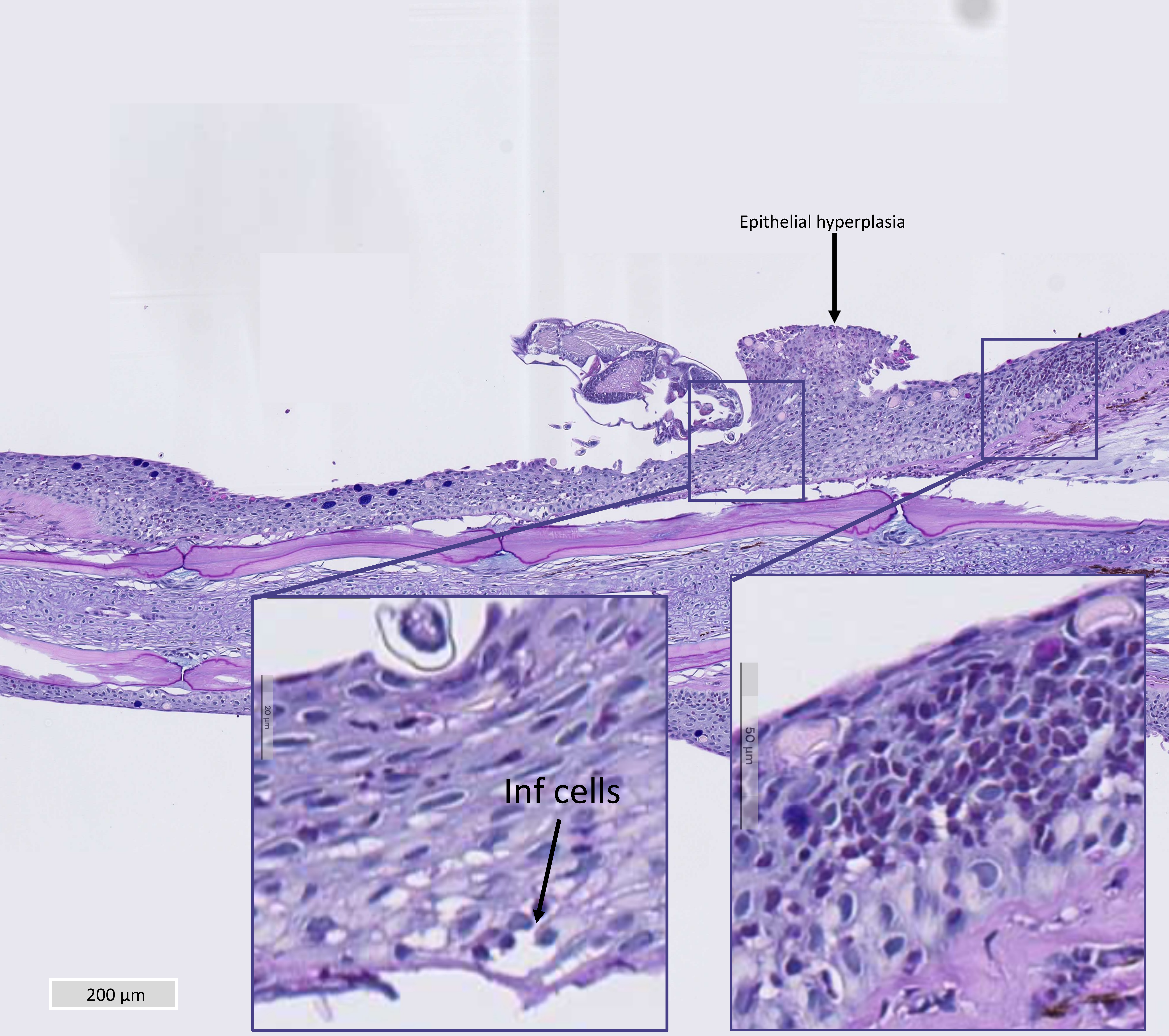

Coho salmon_5_100
Fin Caudal 36 hpi

Section 2_3

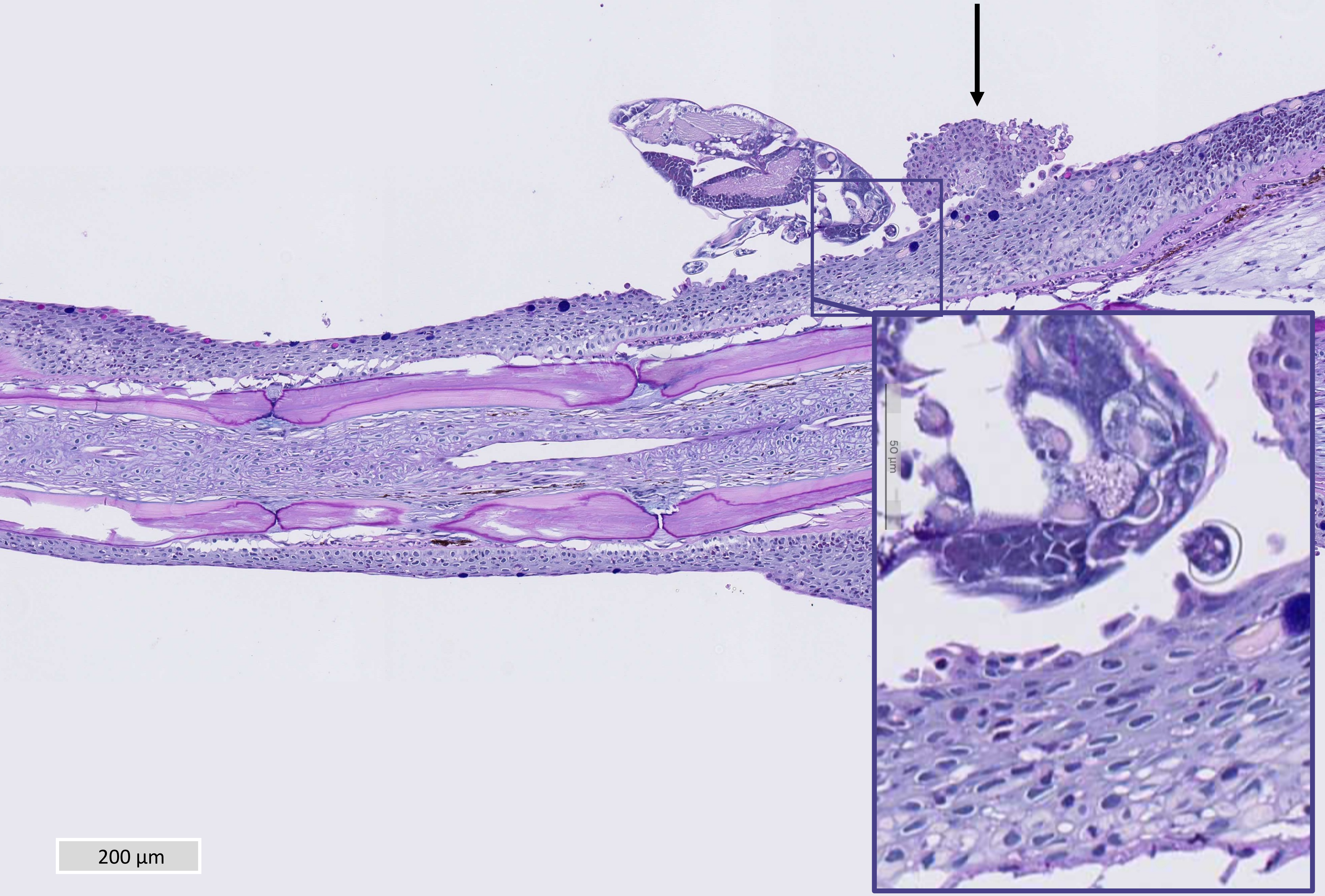

Coho_5_100
Fin_Caudal_36_hpi

Section 3_3

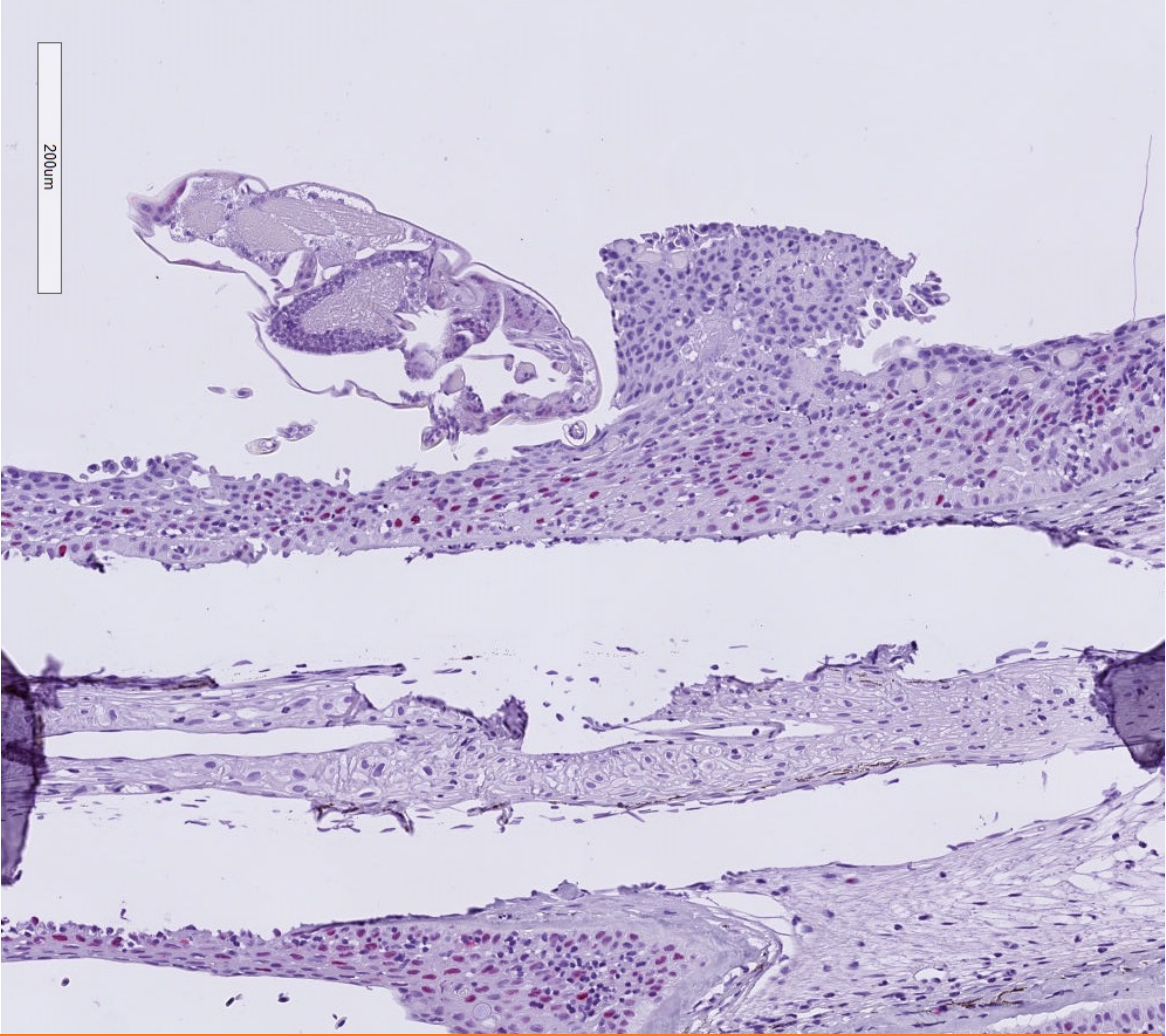

Coho_5_100
Fin_Caudal_36_hpi

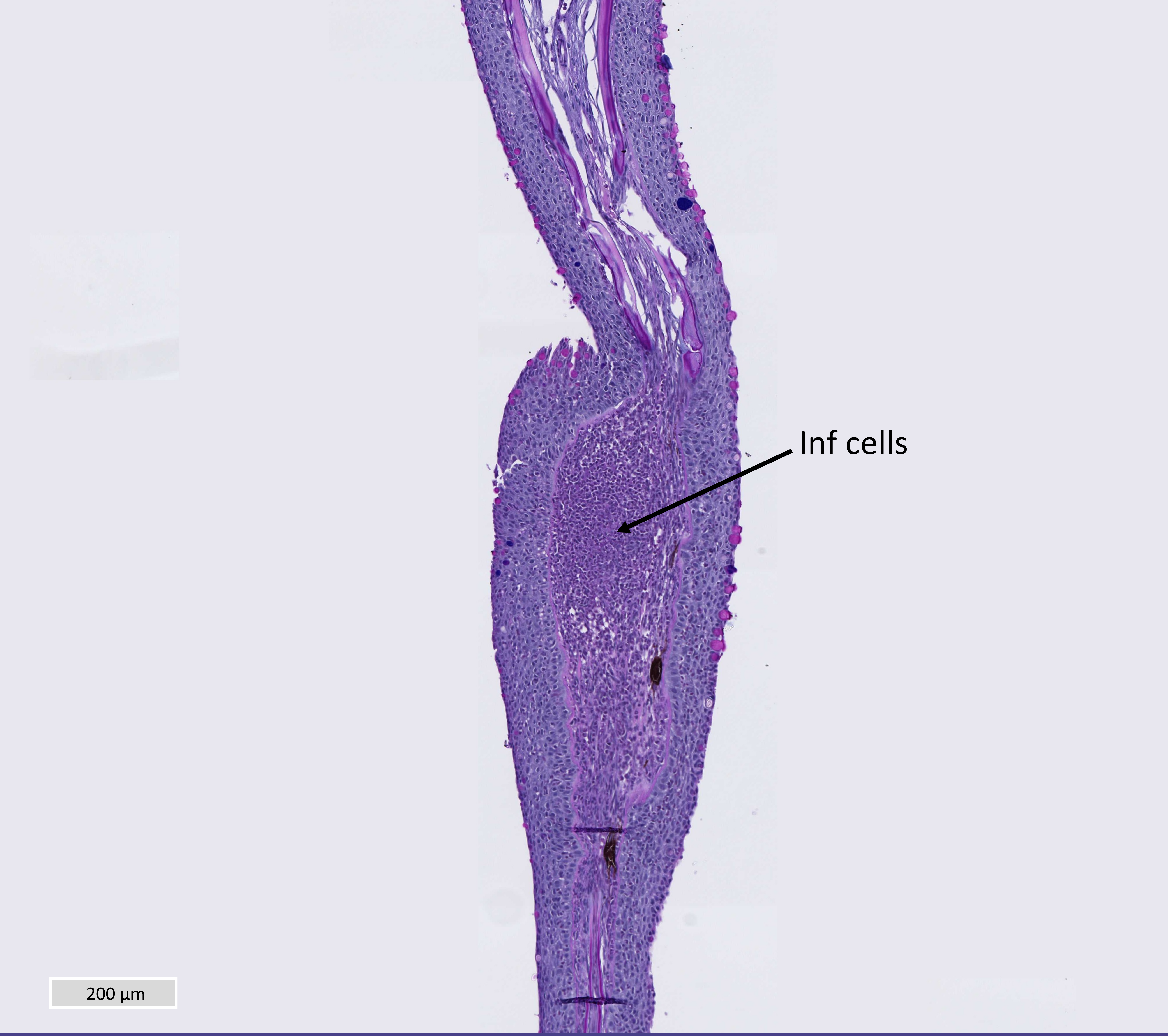

Coho salmon_6_101
Fin_PC_36_hpi

Section 1_2

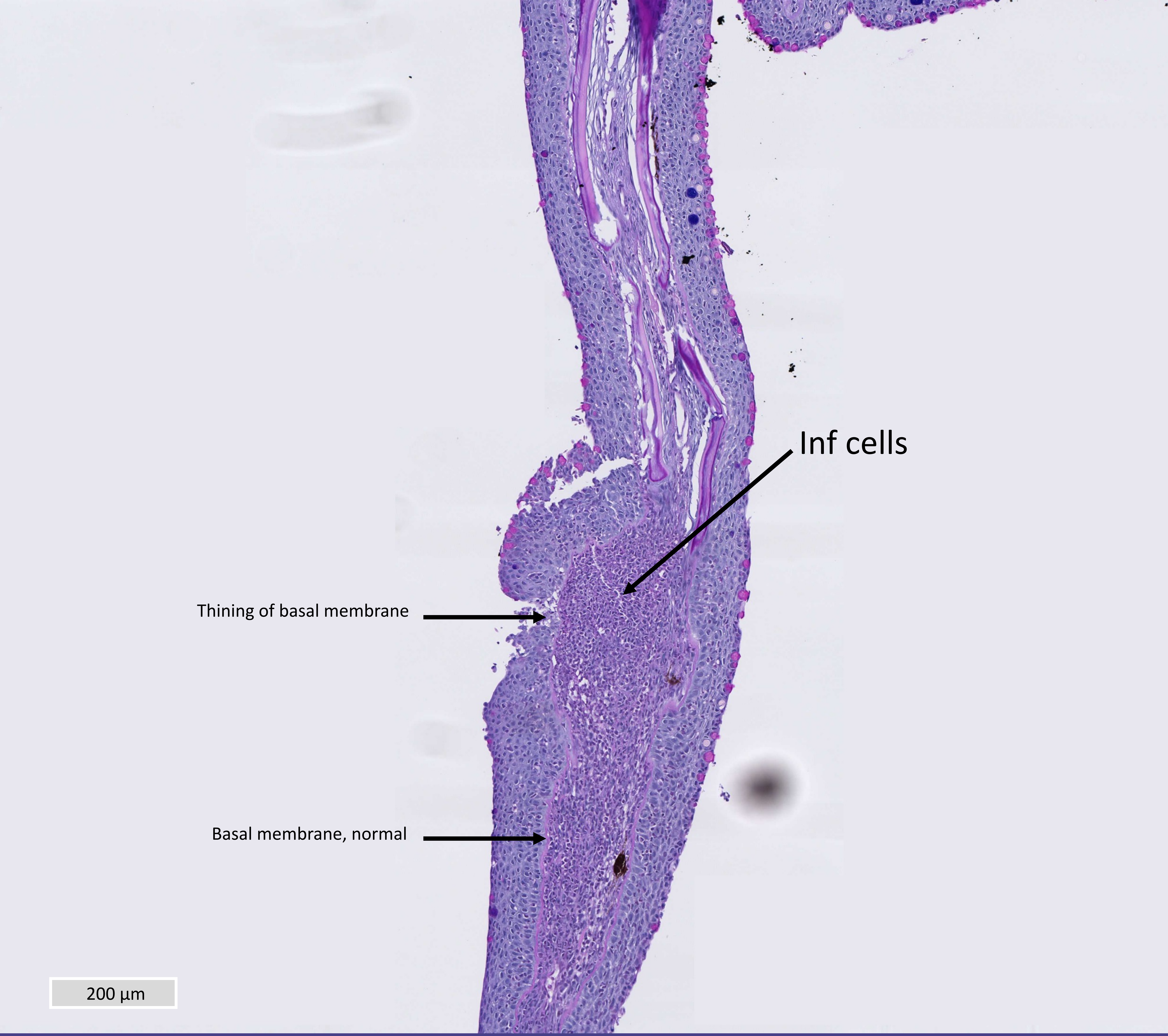

Coho salmon_6_101
Fin_PC_36_hpi

Section 1_2

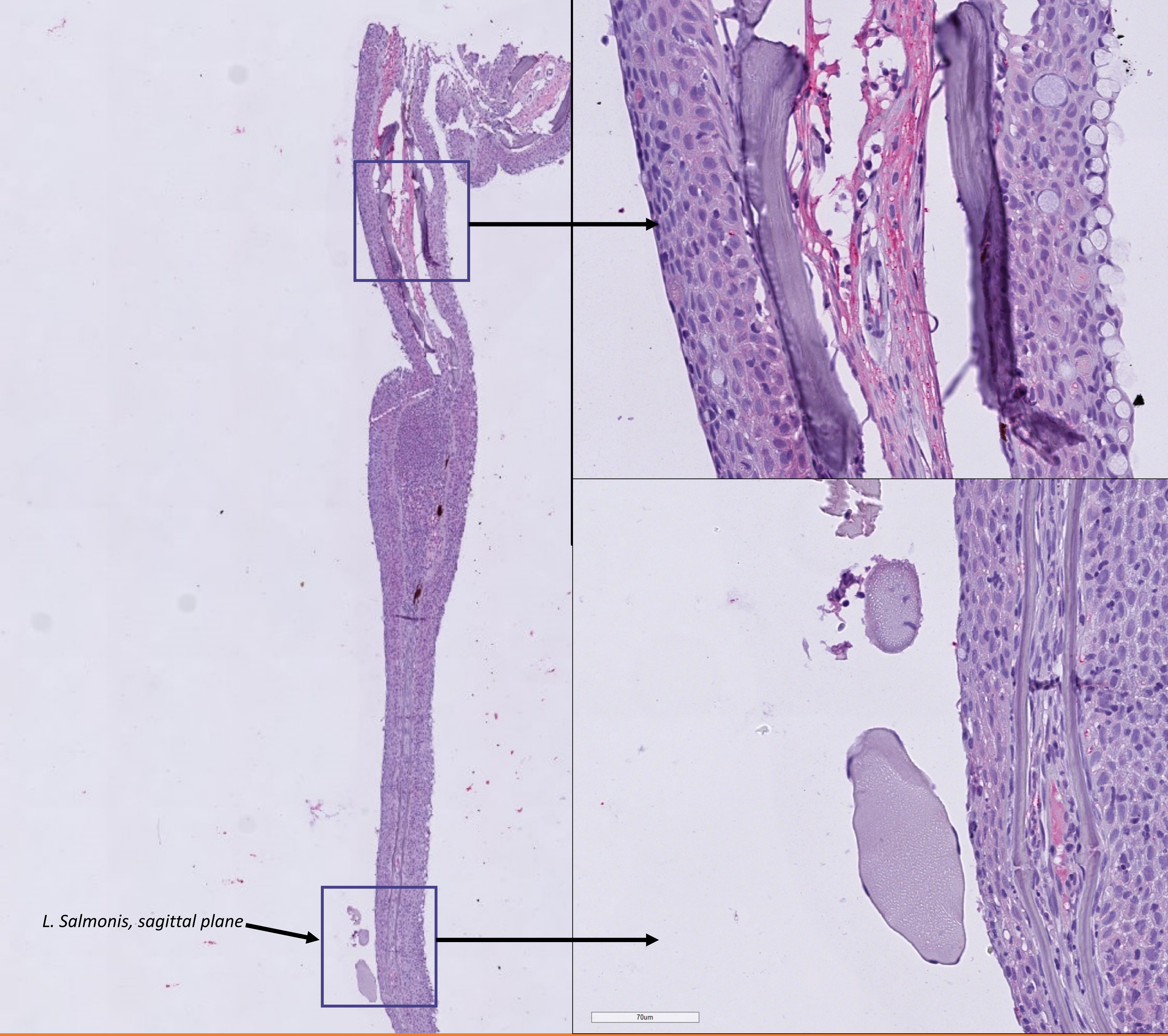

Coho salmon_6_101
Fin_PC_36_hpi

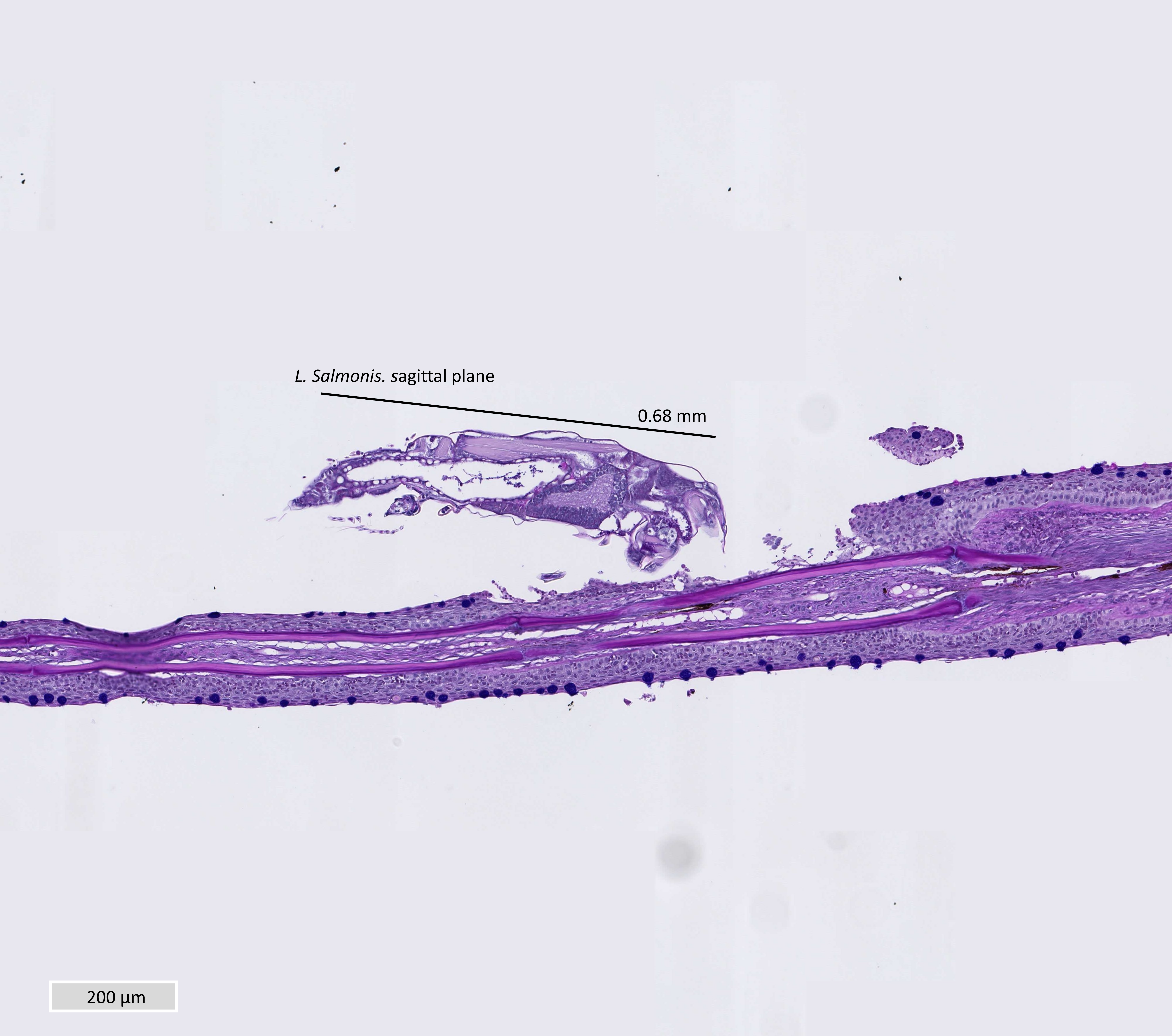

Coho salmon_7_108
Fin_Caudal_36_hpi

Section 1_2

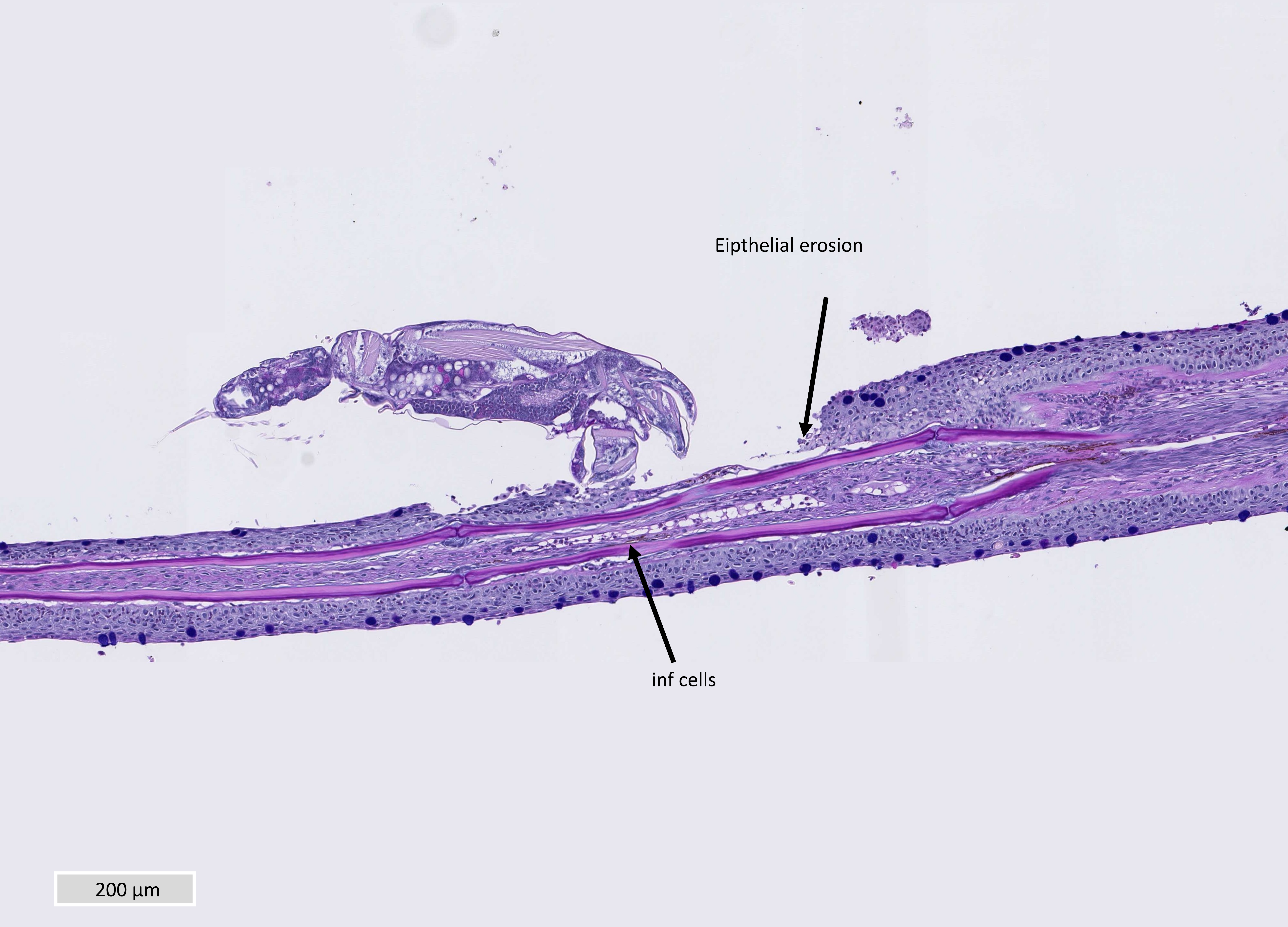

Coho salmon_7_108
Fin_Caudal_36_hpi

Section 2_2

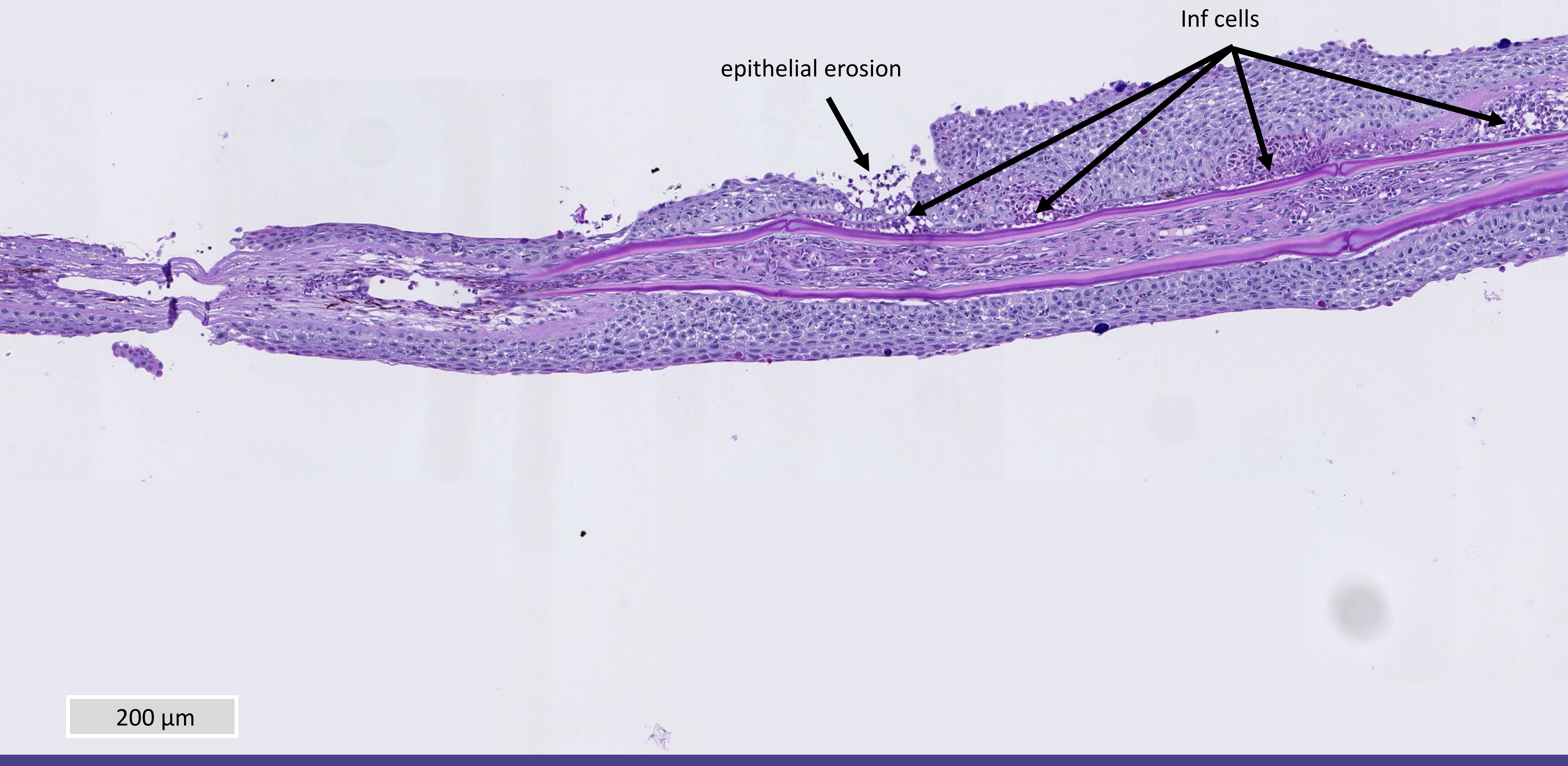

Coho salmon_8_135
Fin_Caudal_48_hpi

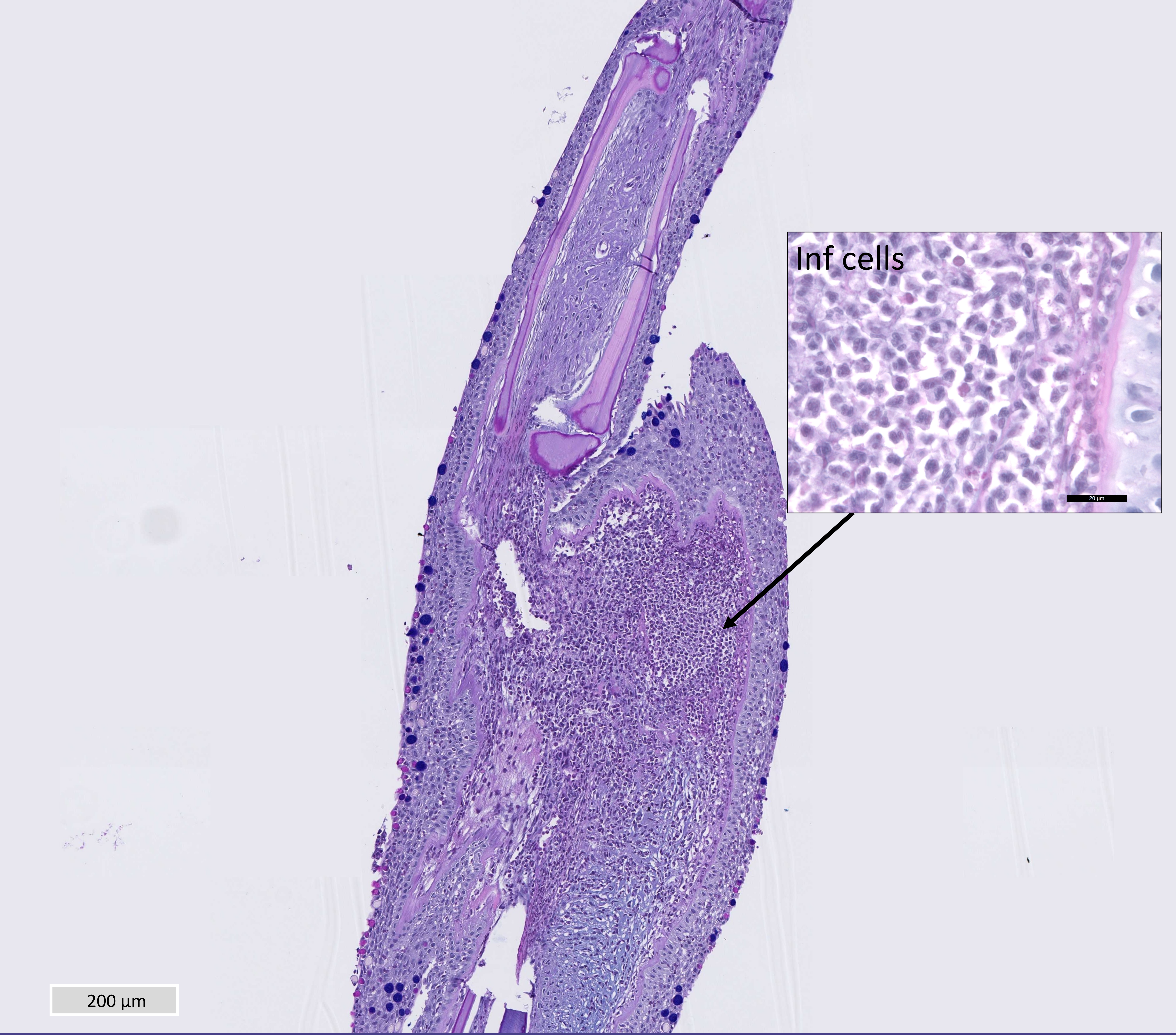

Coho salmon_9_136
Fin_PC_48_hpi

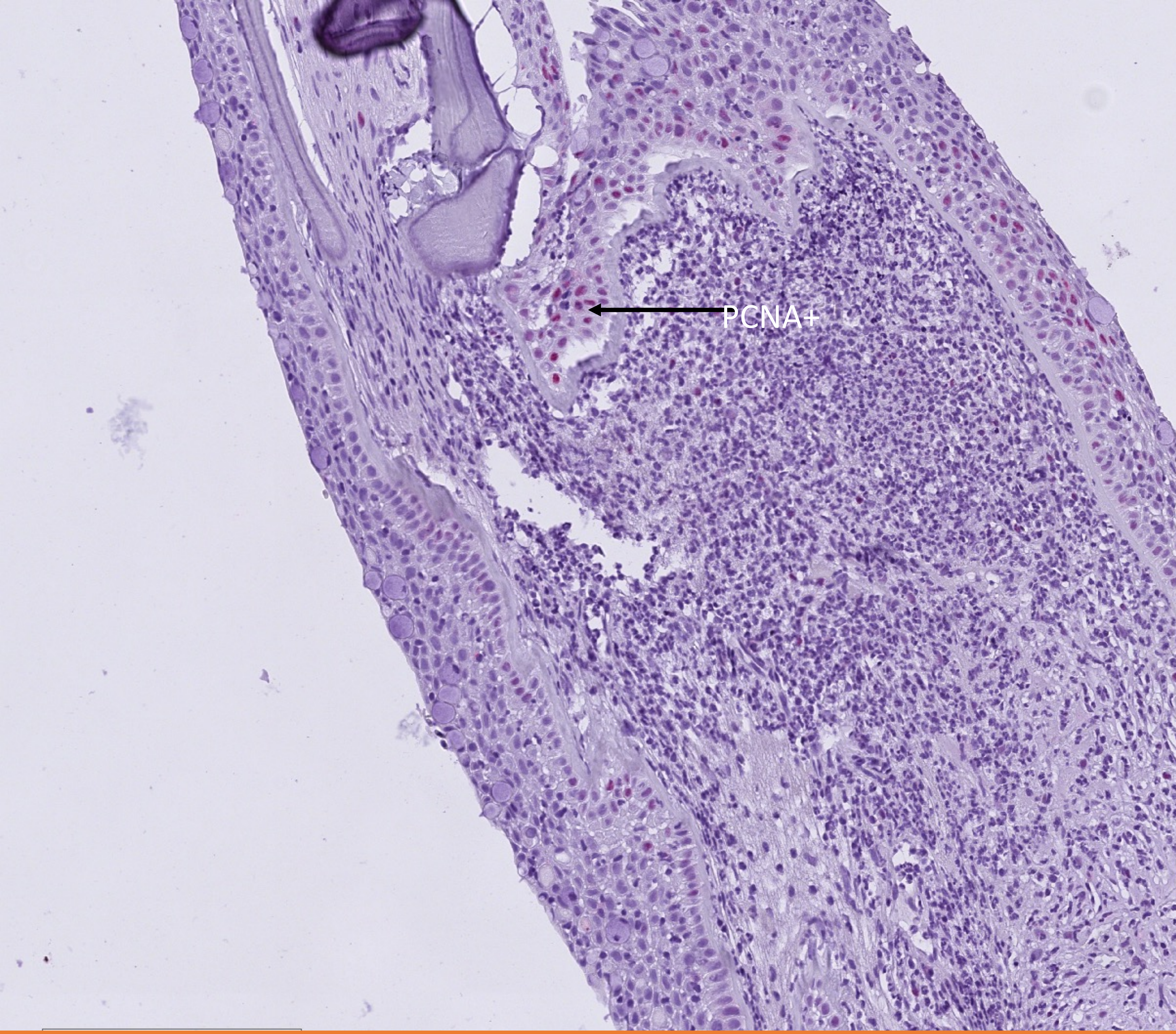

Coho salmon_9_136, IHC PCNA
Fin_PC_48_hpi

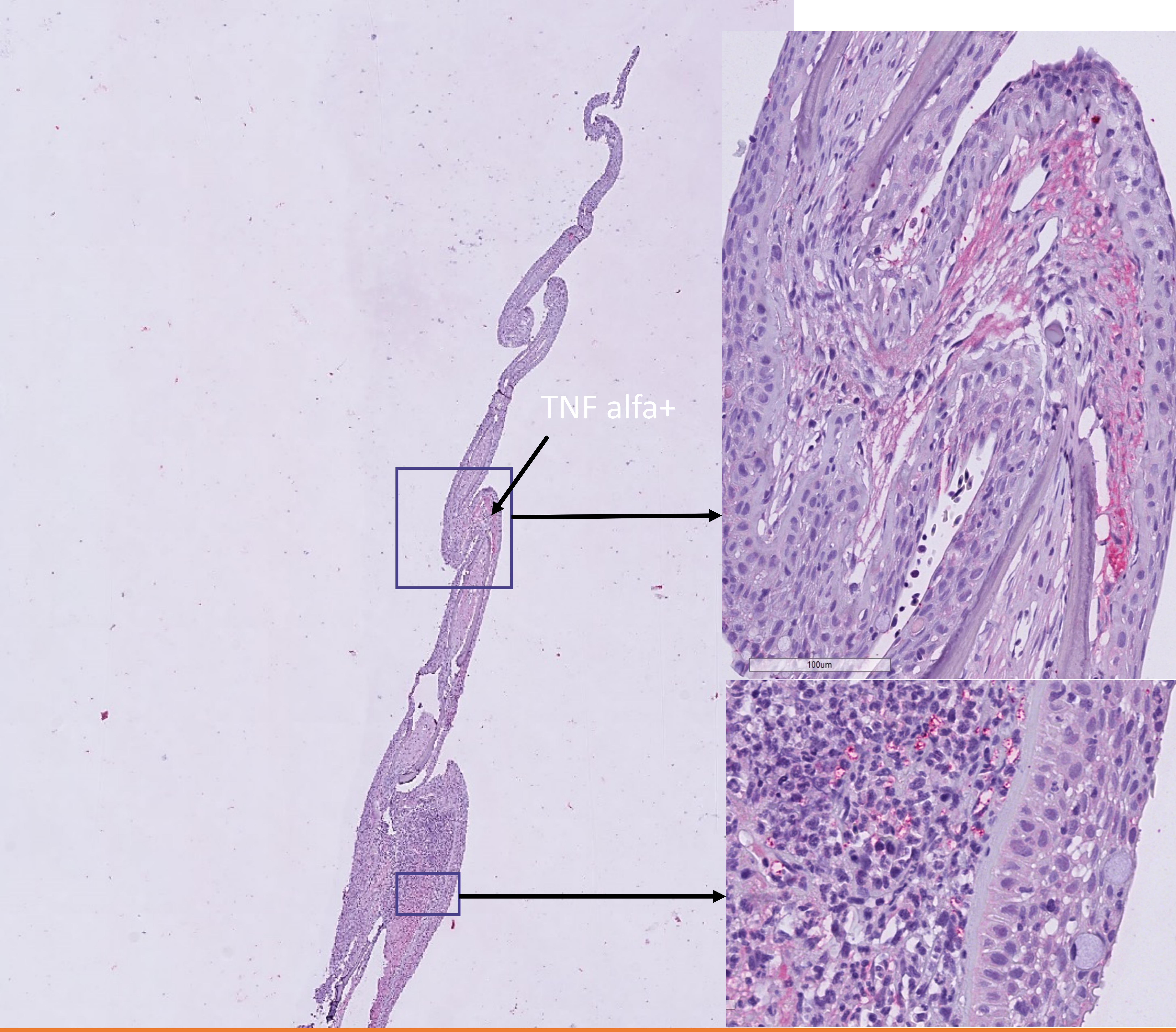

Coho salmon_9_136. IHC TNF alfa
Fin_PC_48_hpi

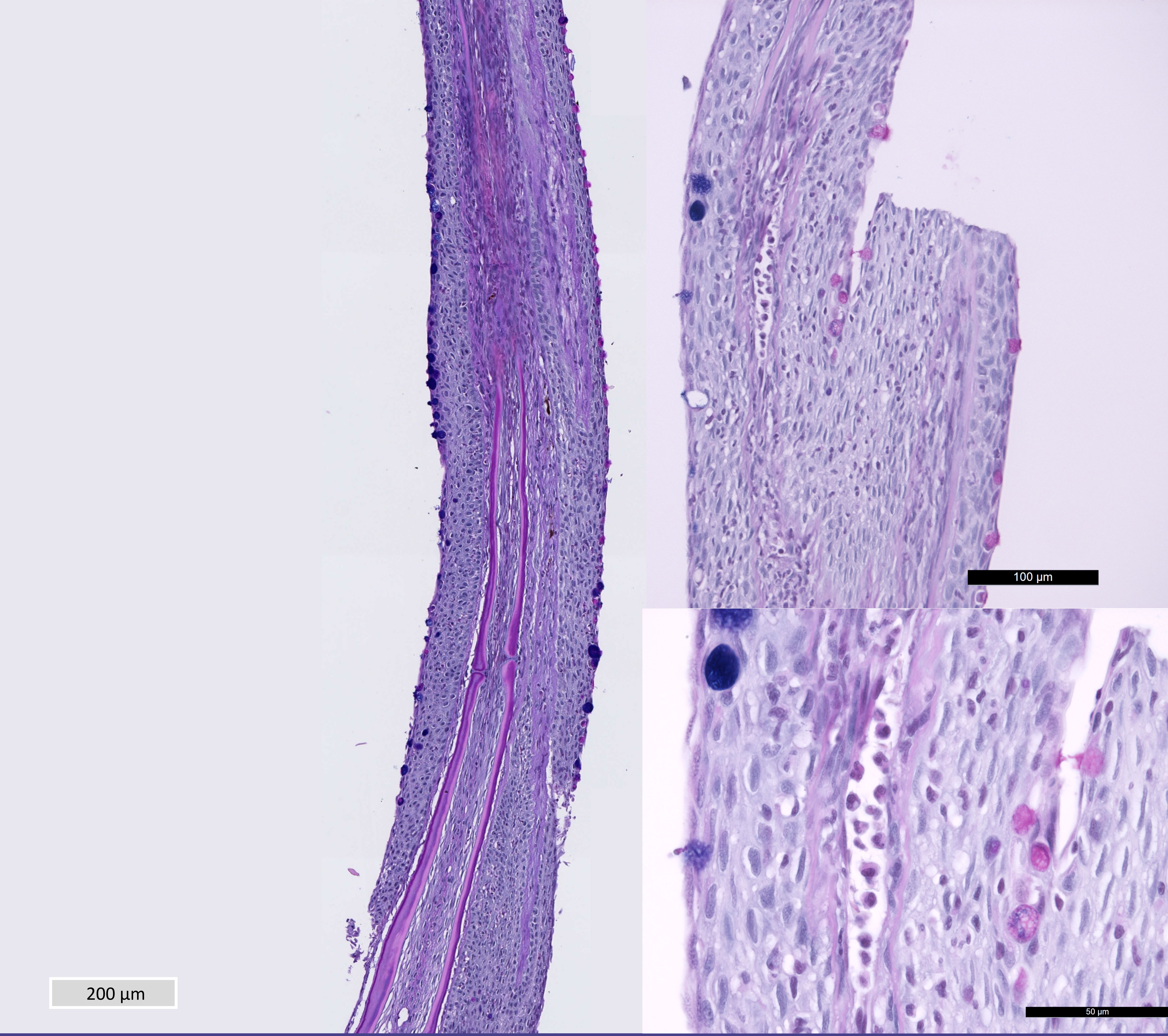

Coho salmon_10_137
Fin_48_hpi

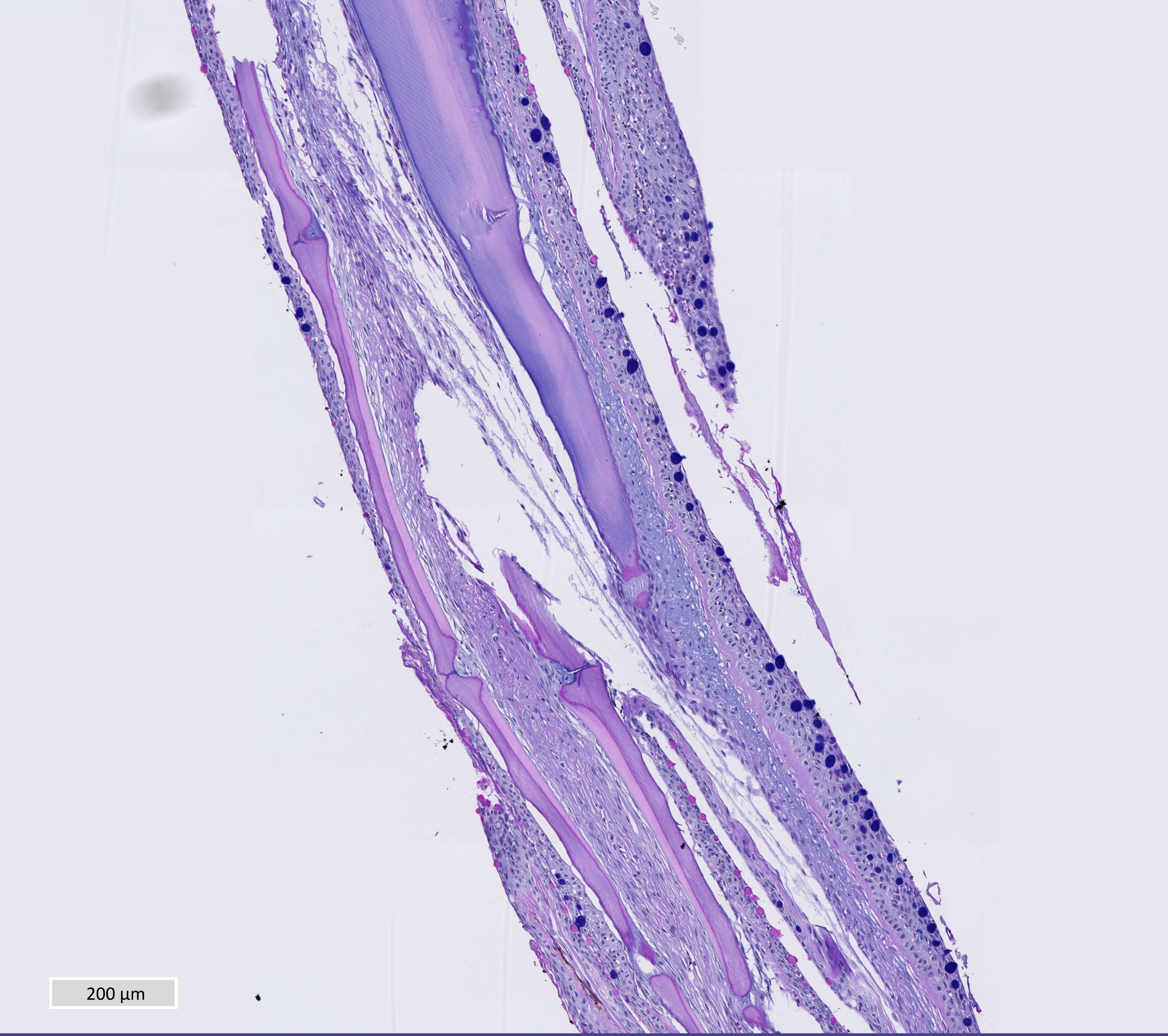

Coho salmon_11_139
Fin_48_hpi

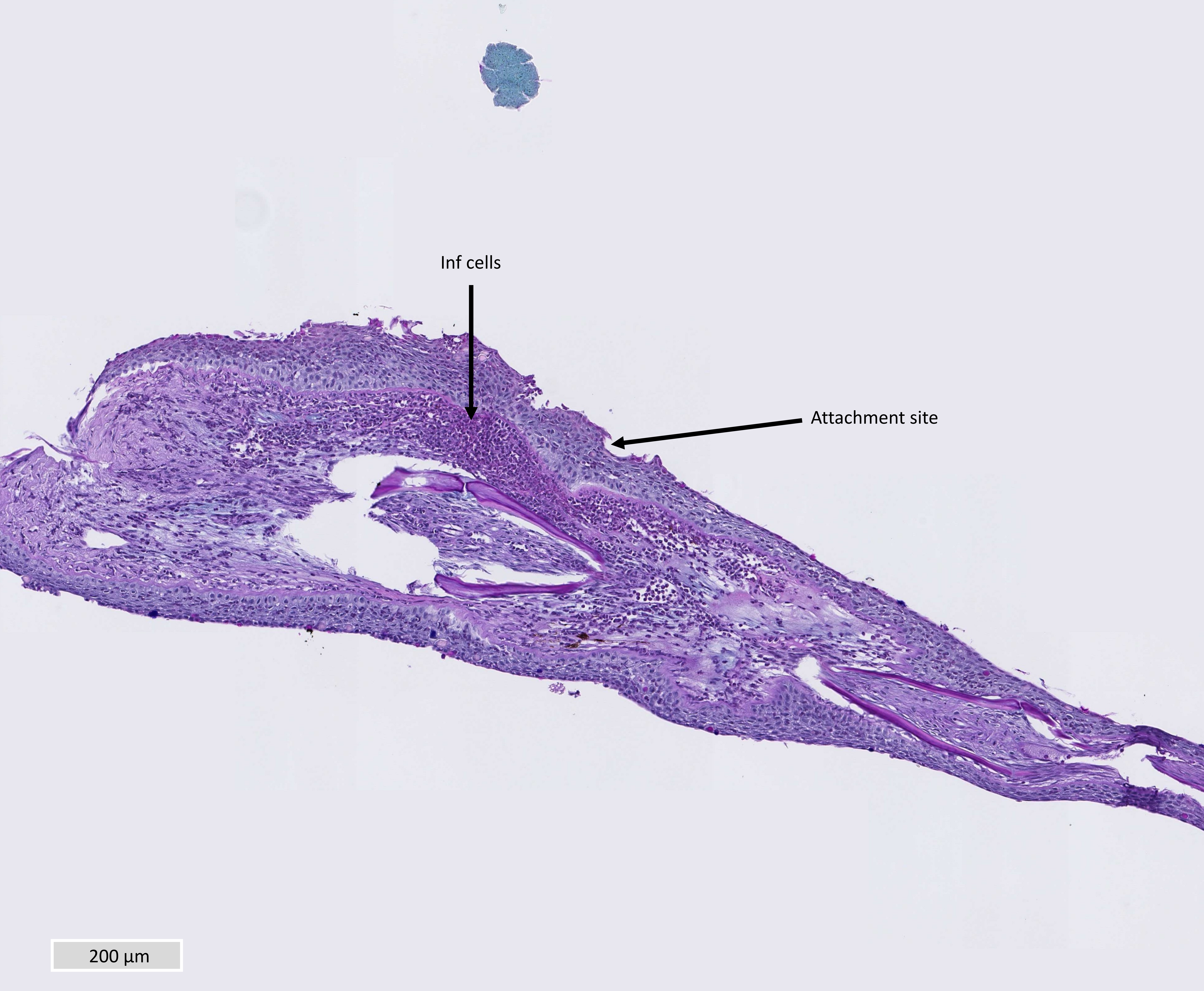

Coho salmon_12_141
Fin_Dorsal_48_hpi

Section 1_3

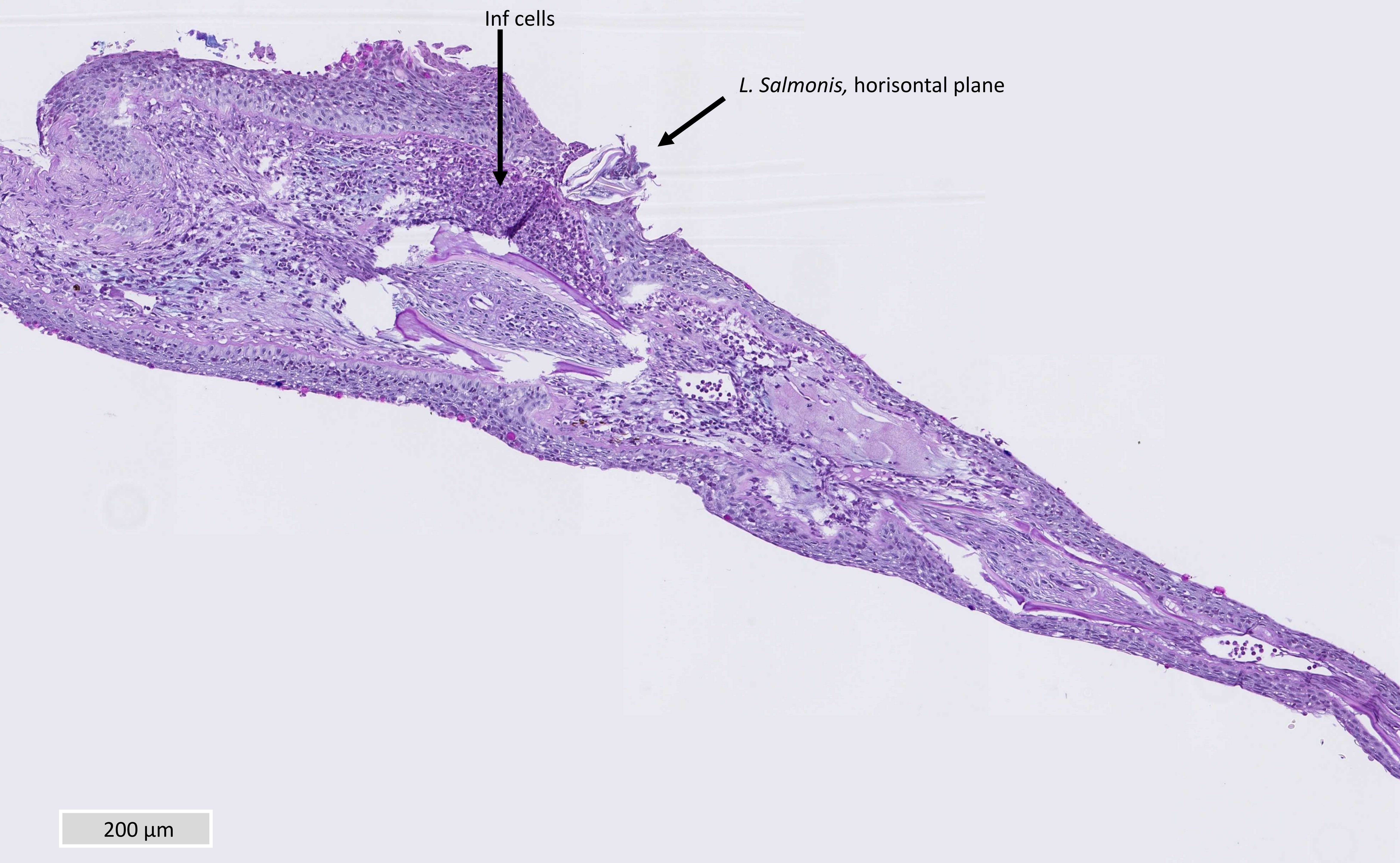

Coho salmon_12_141
Fin_Dorsal_48_hpi

Section 2_3

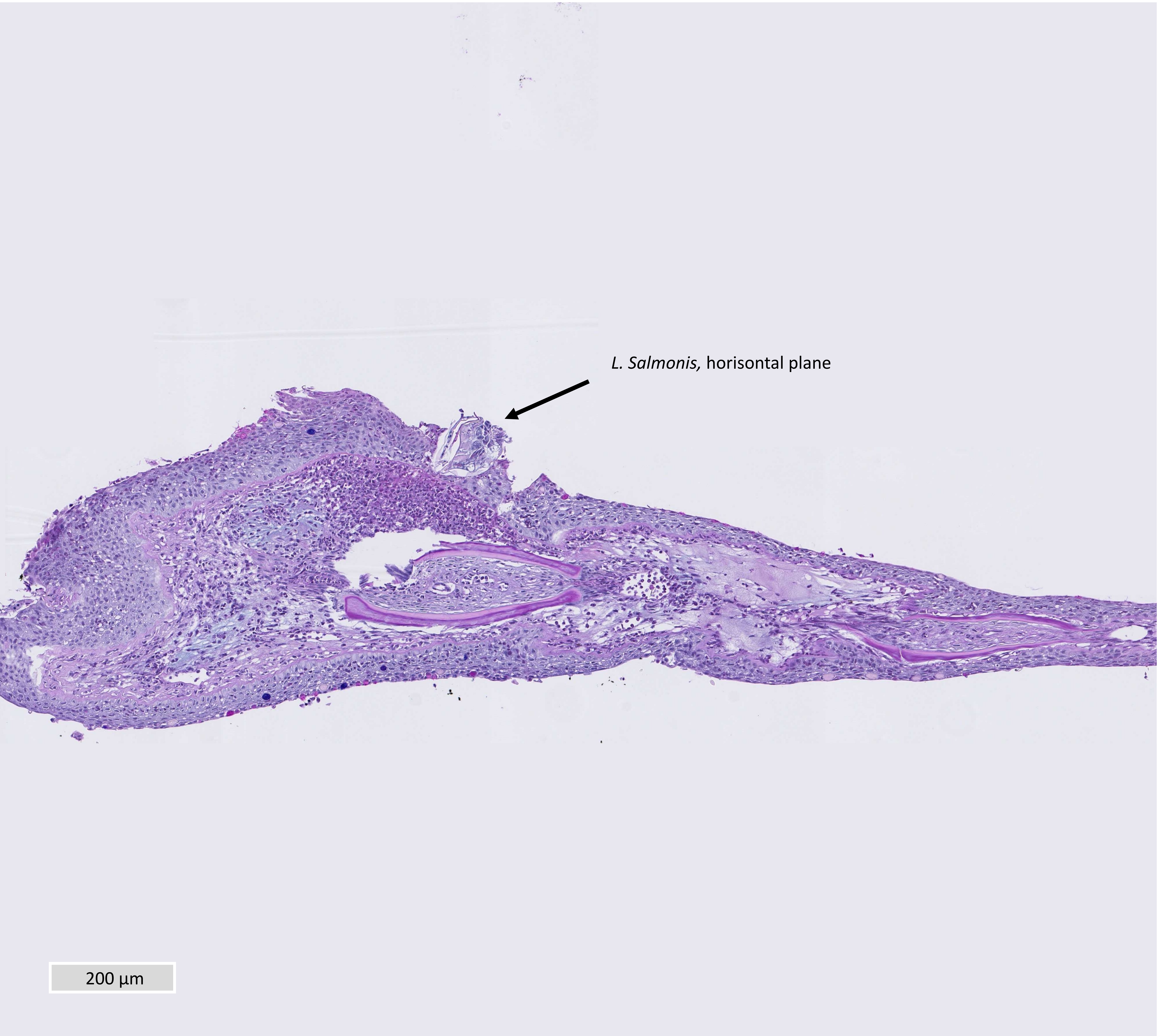

Coho salmon_12_141
Fin_Dorsal_48_hpi

Section 3_3

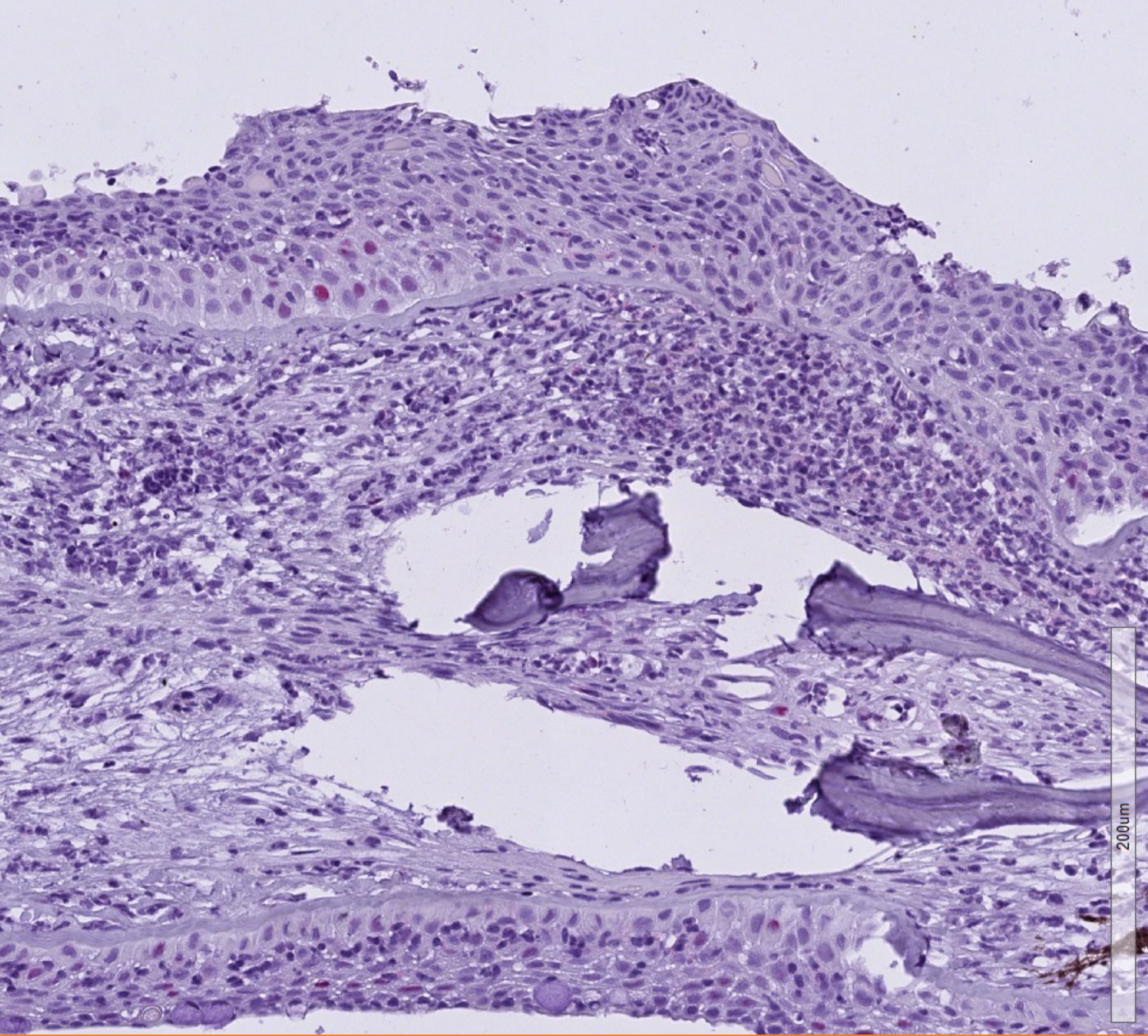

Coho salmon_12_141

Fin_Dorsal_48_hpi

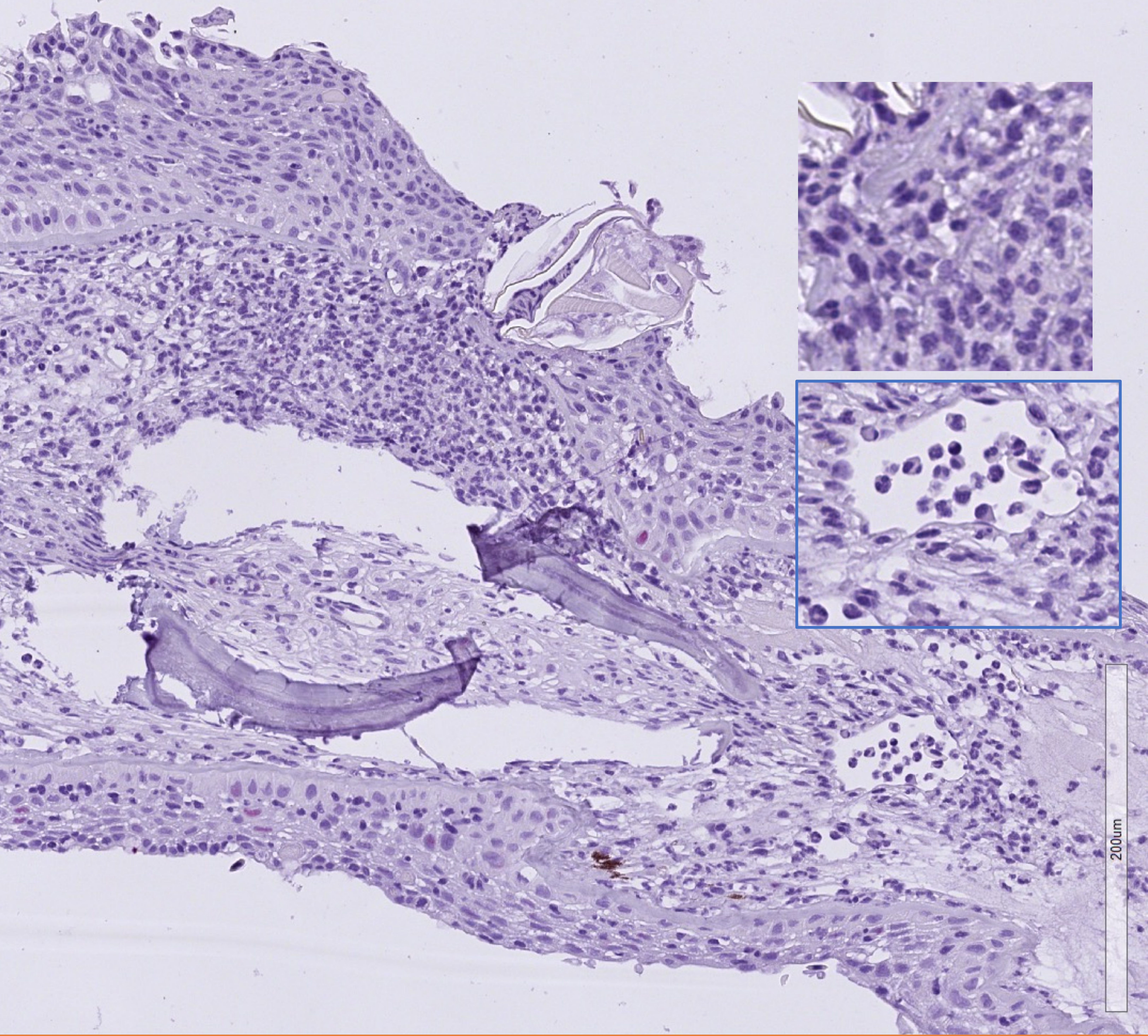

Coho salmon_12_141

Fin_Dorsal_48_hpi

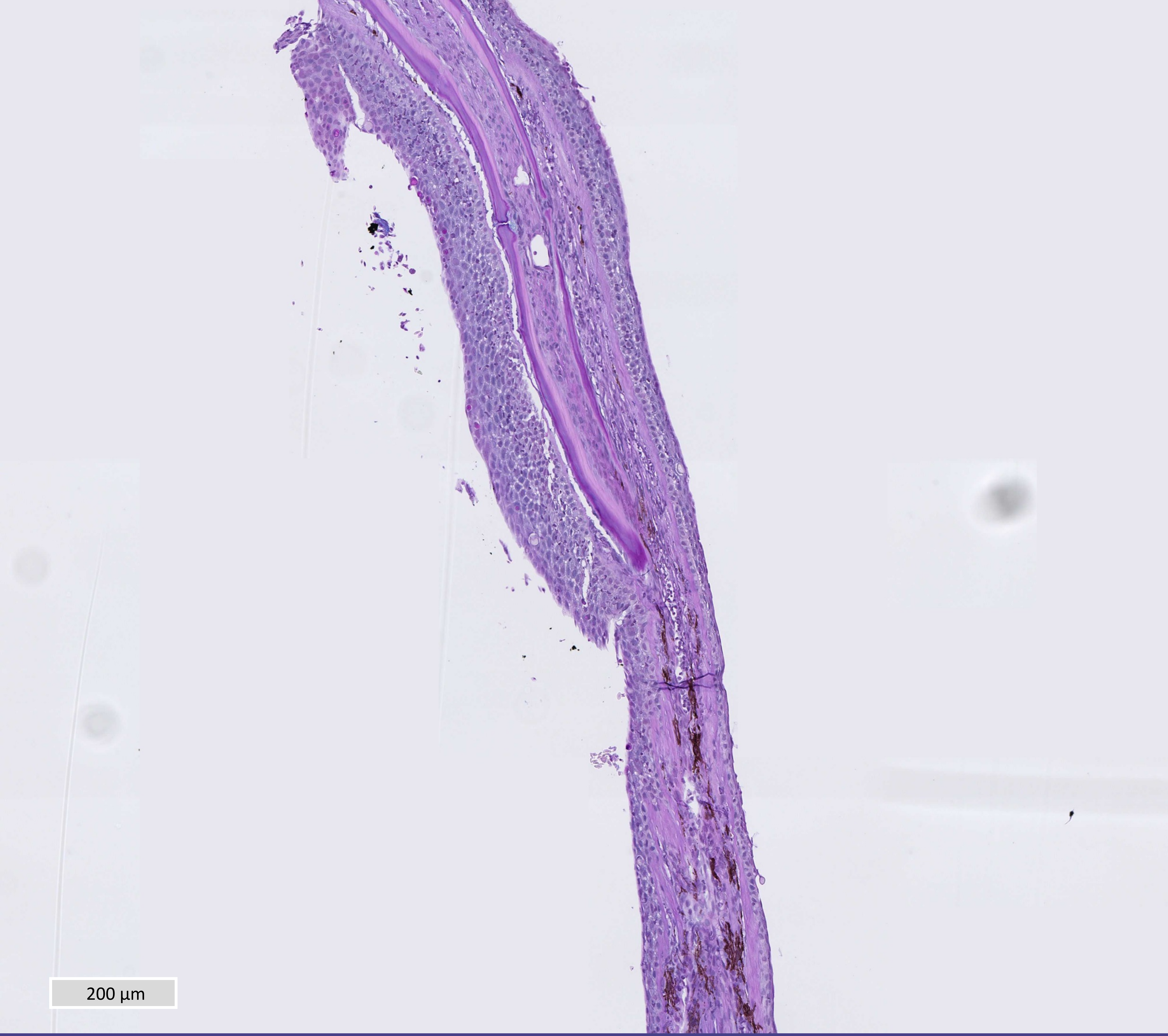

Coho_13_142
Fin_Caudal_48_hpi

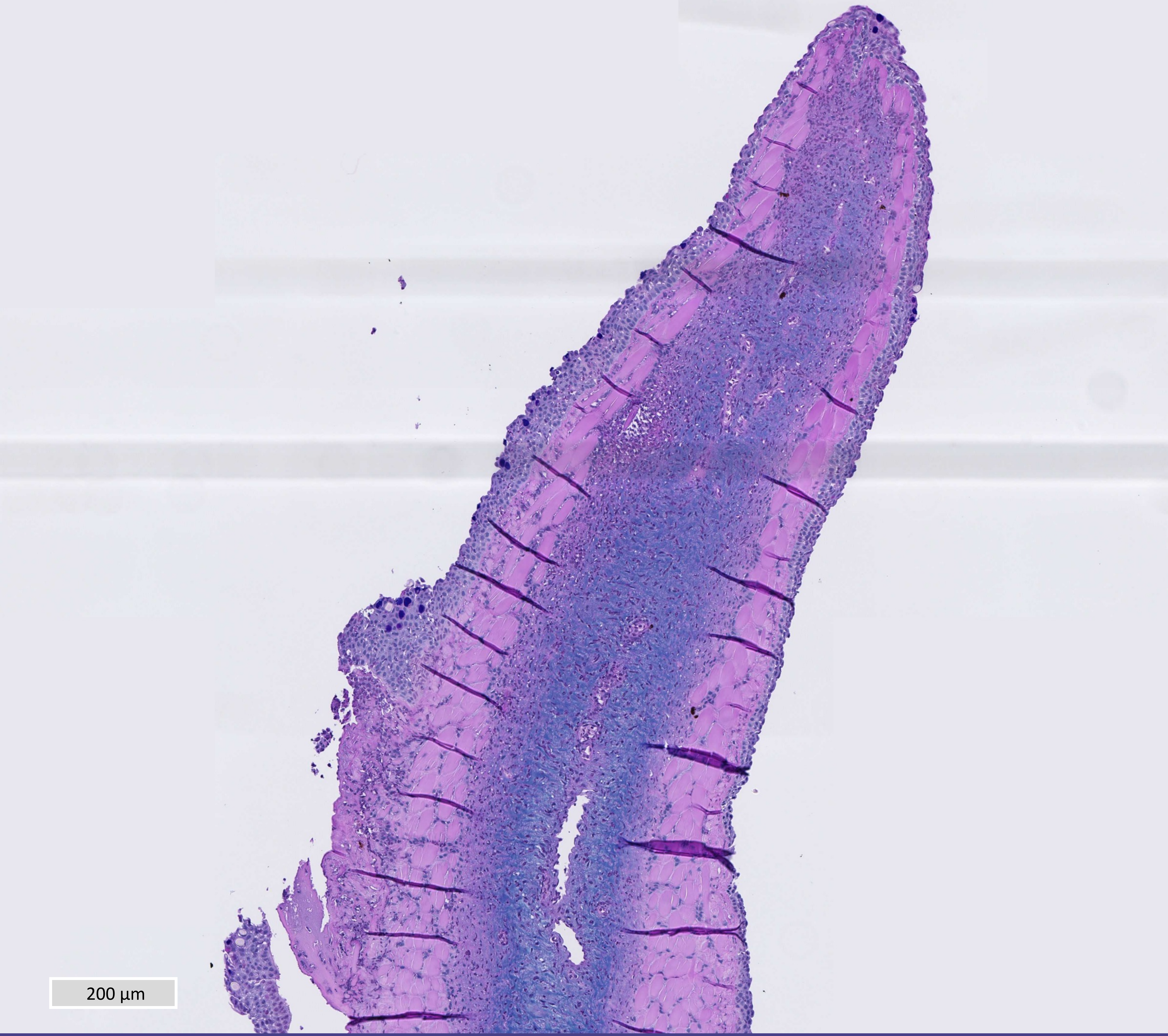

Coho_14_143
Fin_Caudal_48_hpi

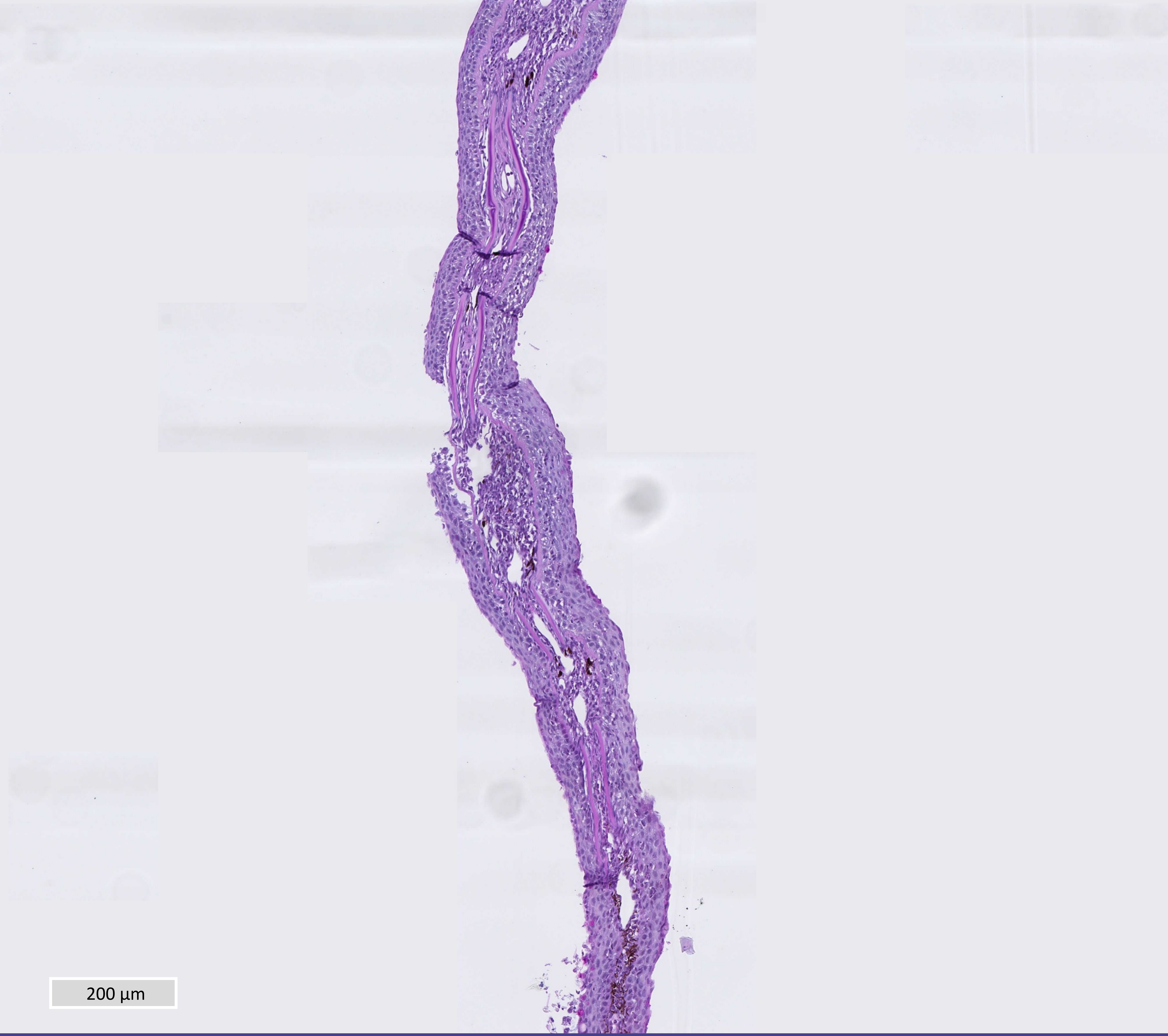

Coho_15_175
Fin_Caudal_168_hpi

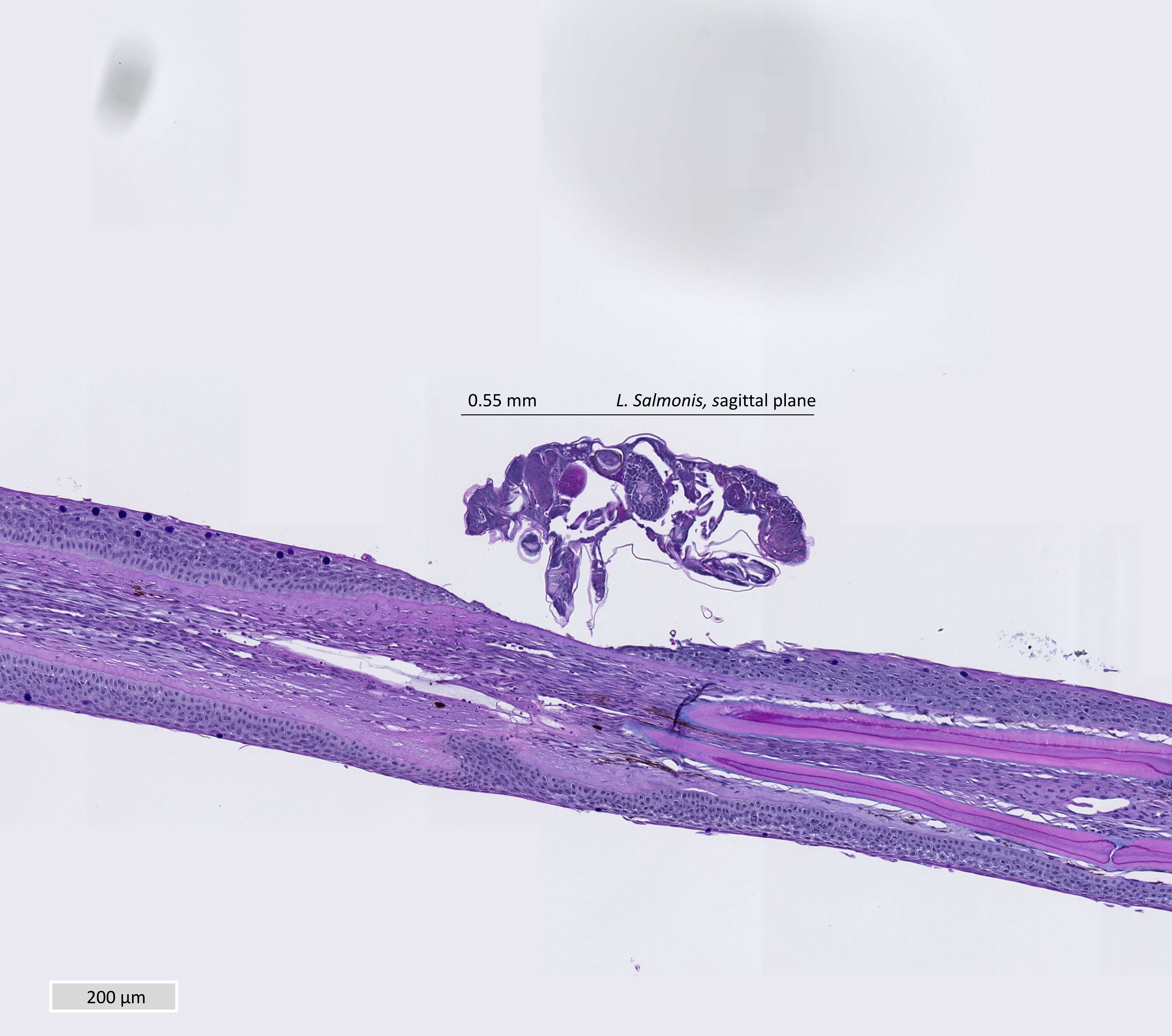

Atlantic salmon_1_1
Caudal fin_12_hpi

Section 1

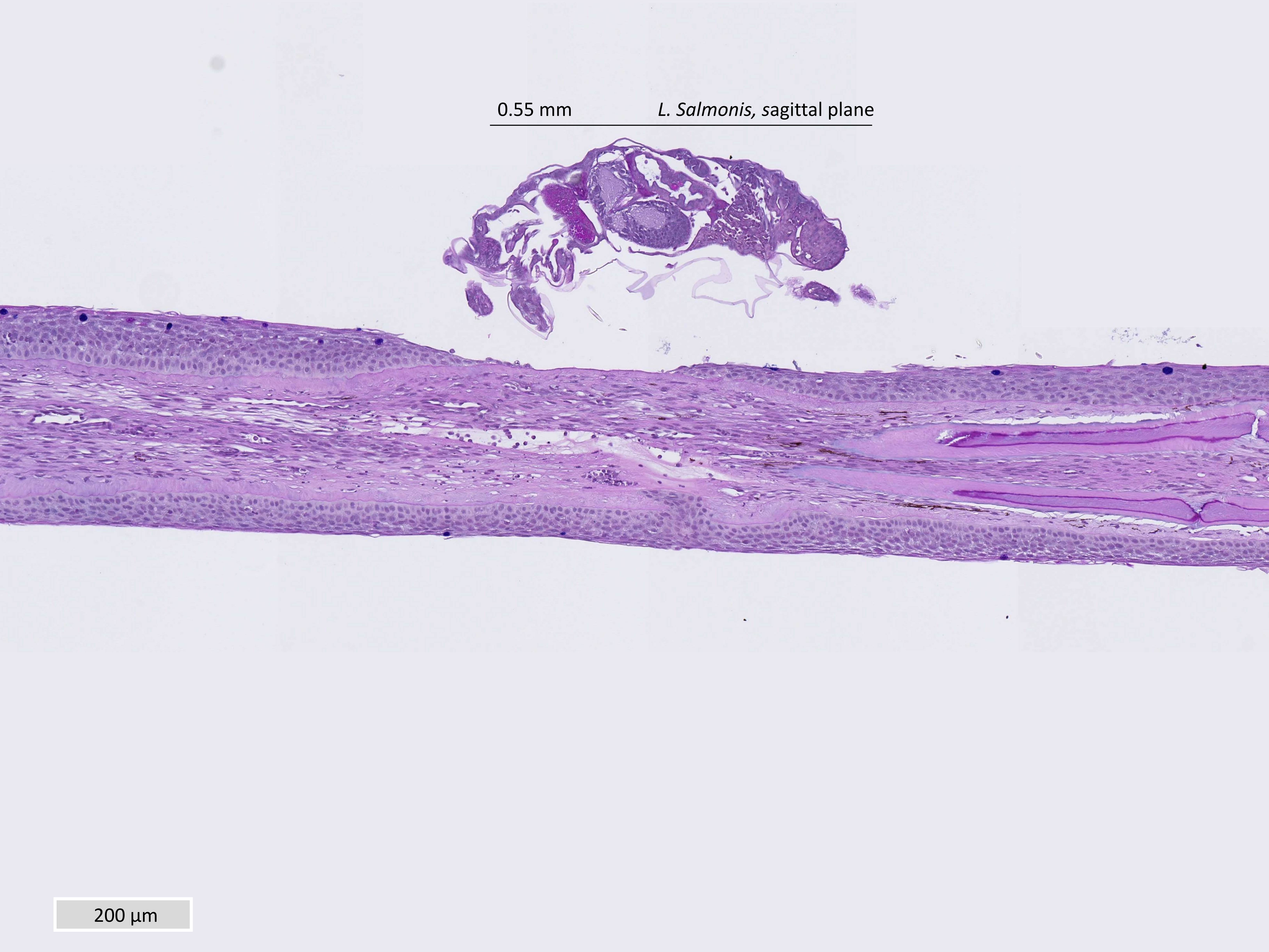

Atlantic salmon_1_1
Caudal fin_12_hpi

Section 2

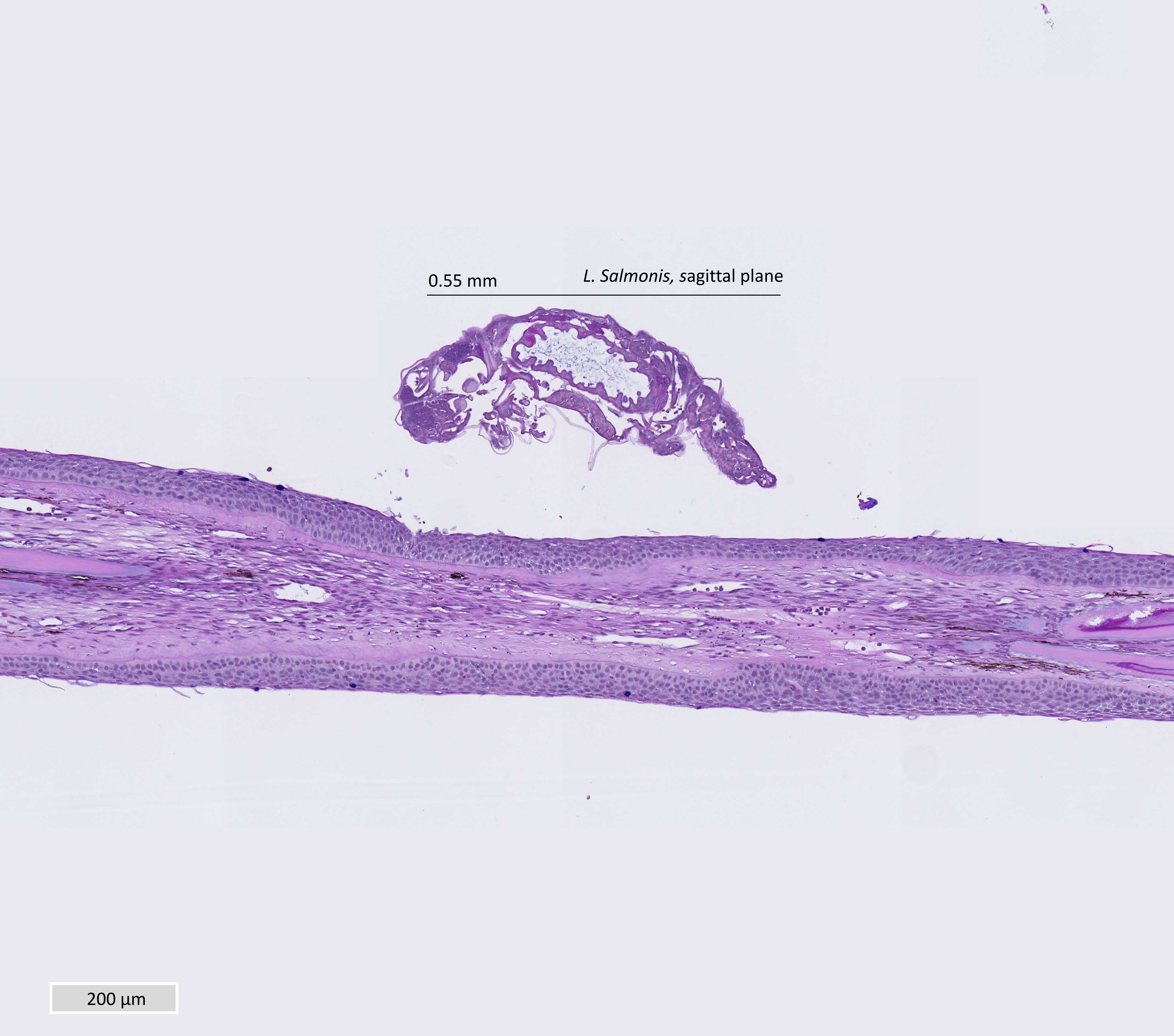

Atlantic salmon_1_1
Caudal fin_12_hpi

Section 3

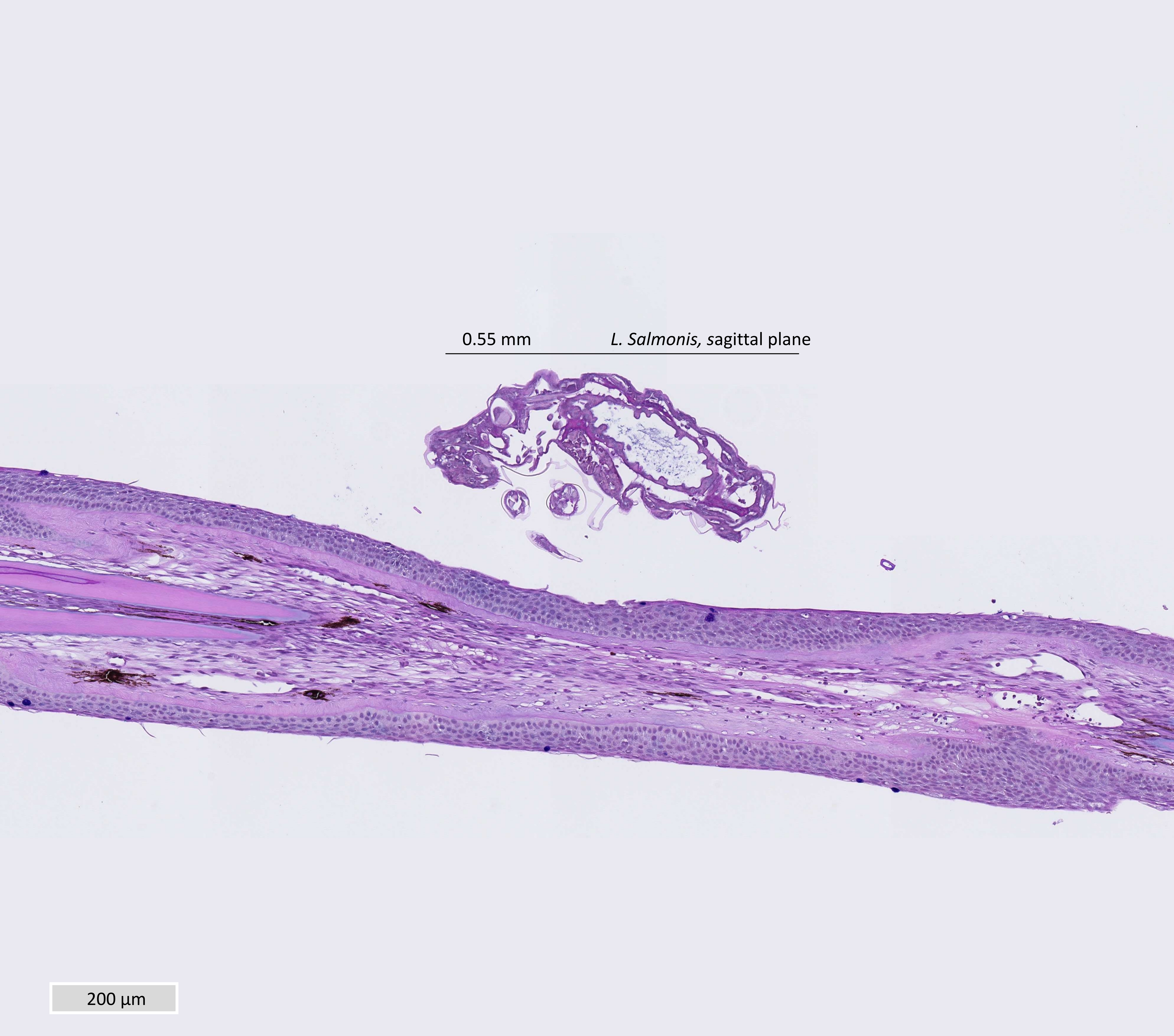

Atlantic salmon_1_1
Caudal fin_12_hpi

Section 4

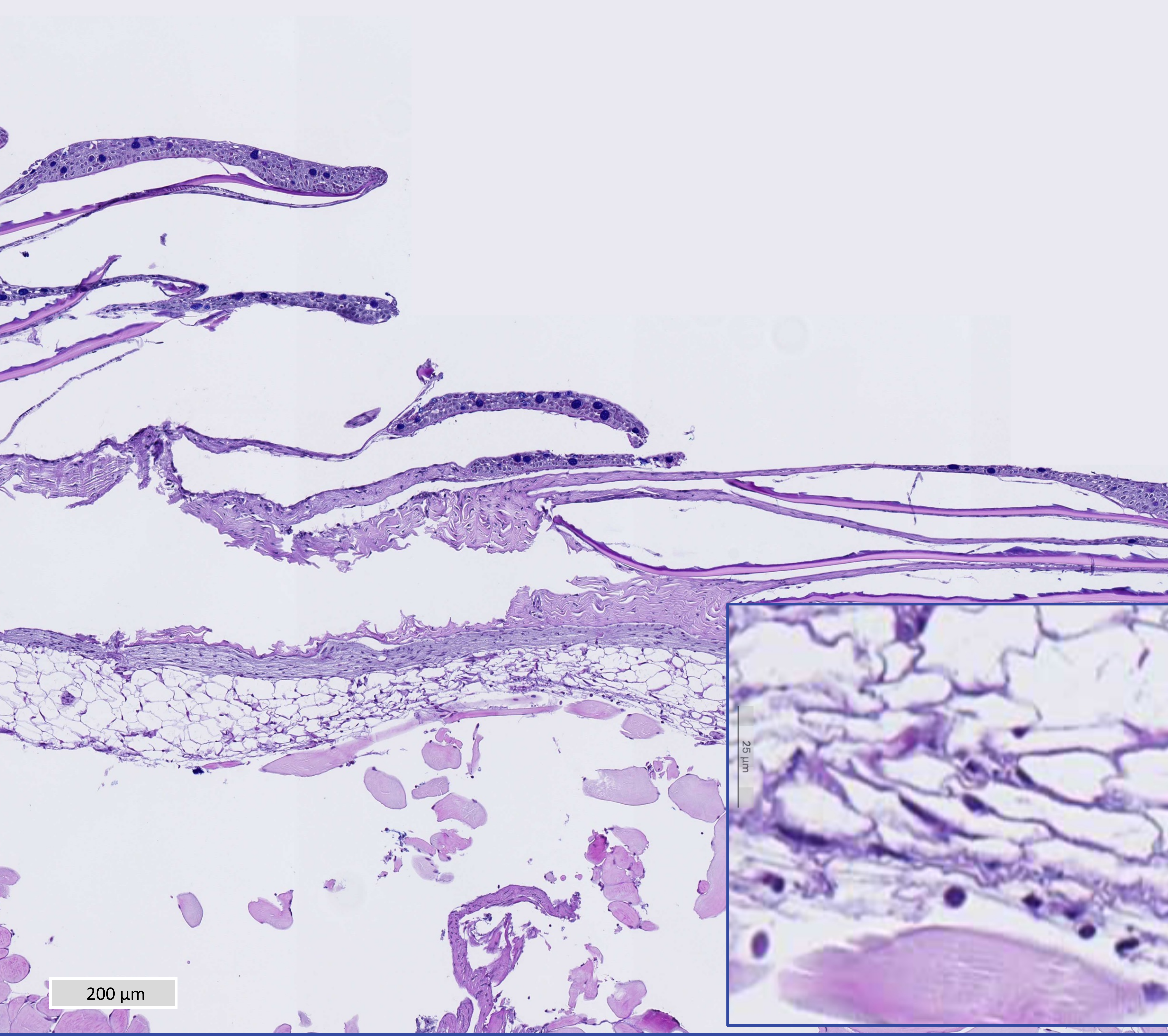

Atlantic salmon_2_7
Scaly skin_12_hpi

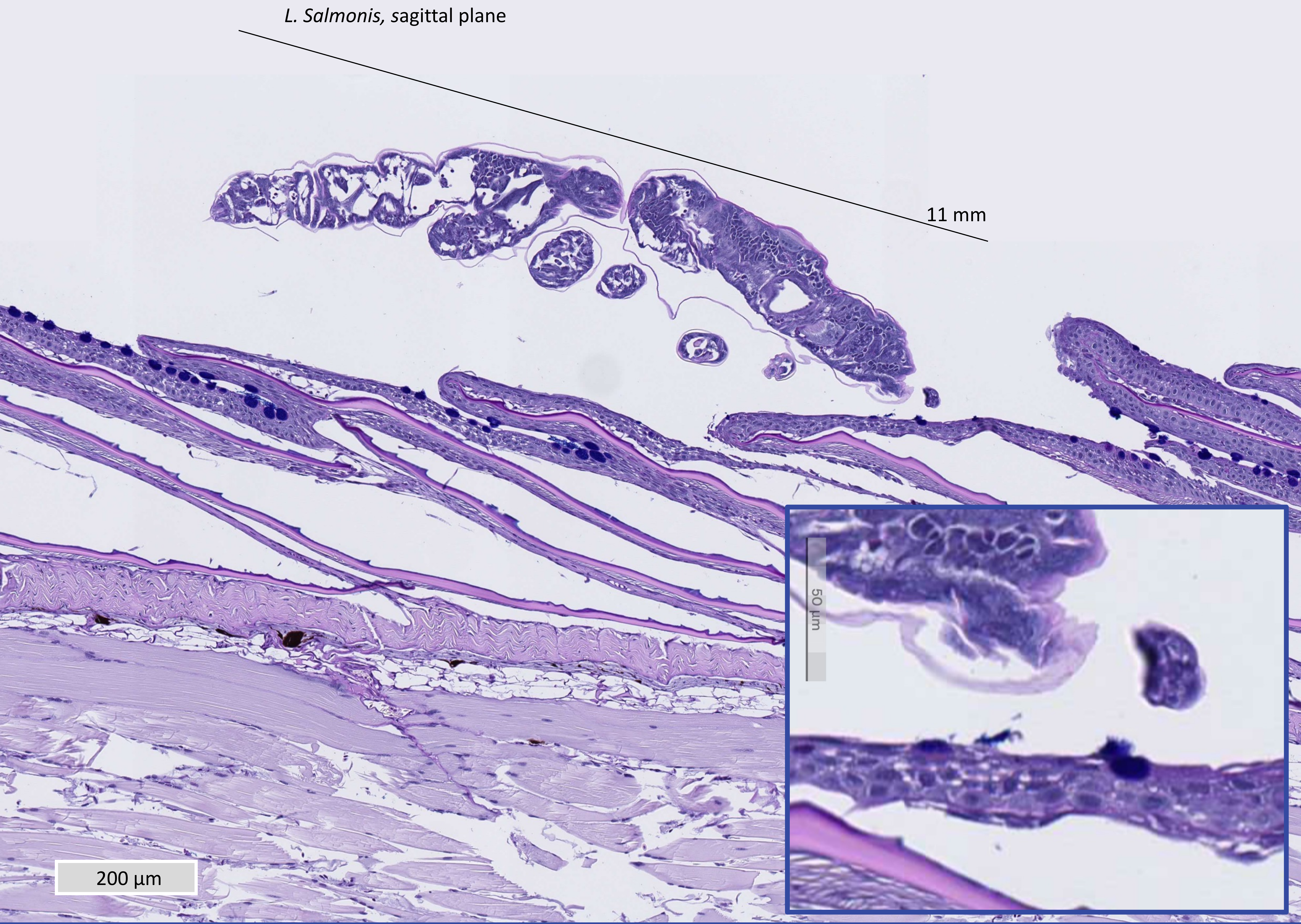

Atlantic salmon_3_8

Scaly skin_12_hpi

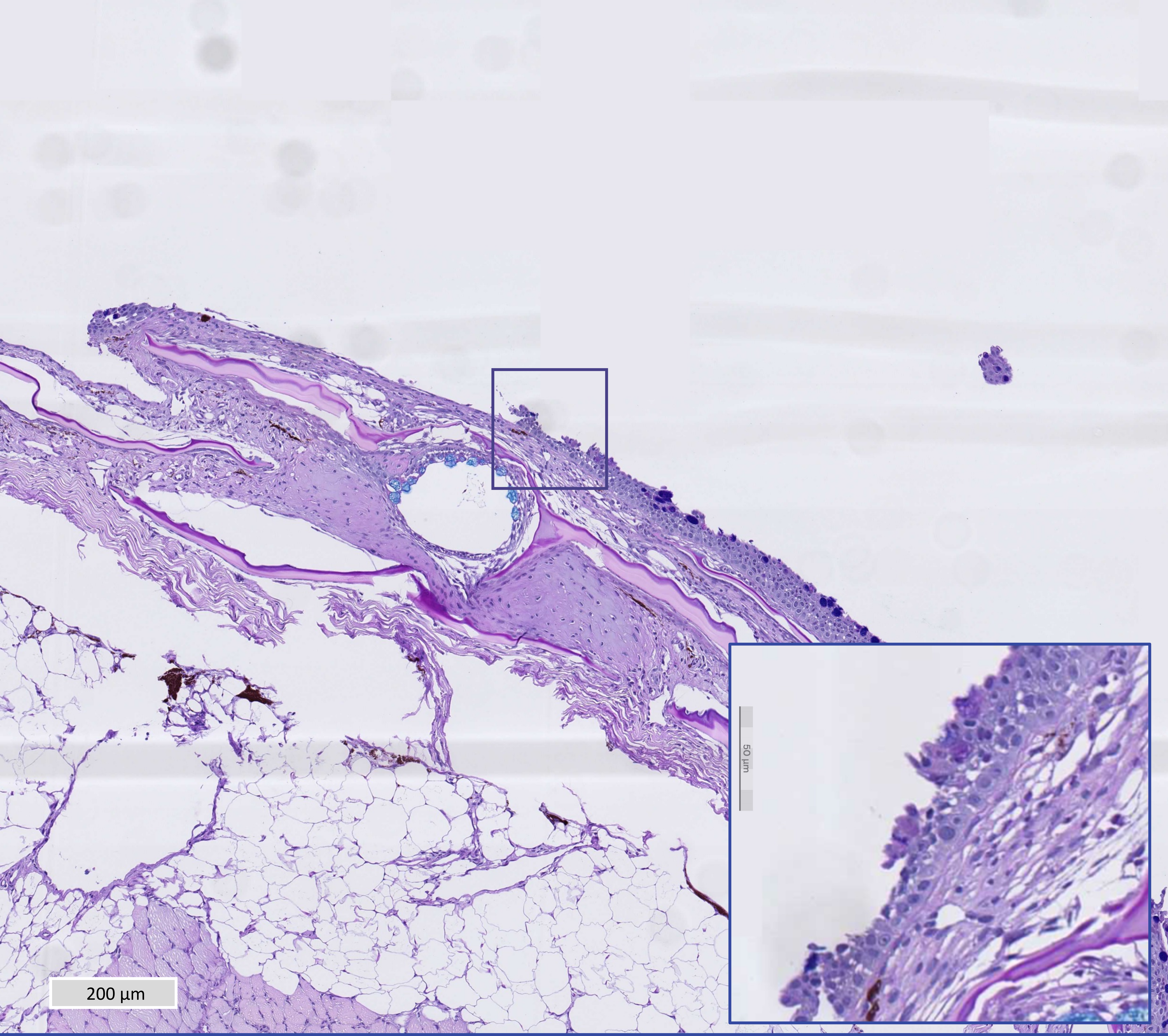

Atlantic salmon_4_39

Scaly skin_24_hpi

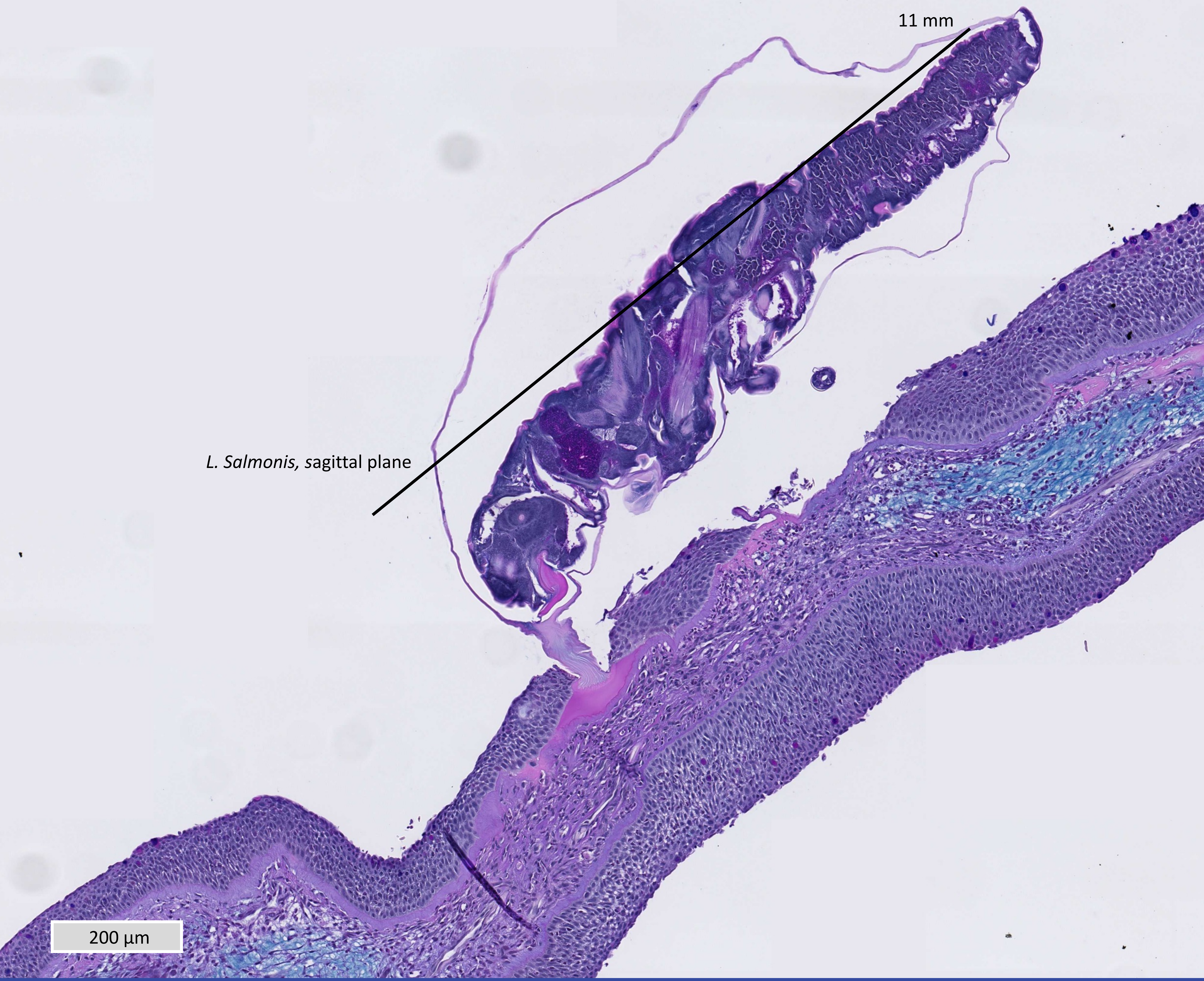

Atlantic salmon_5_41
Scaly skin_24_hpi

Section 1

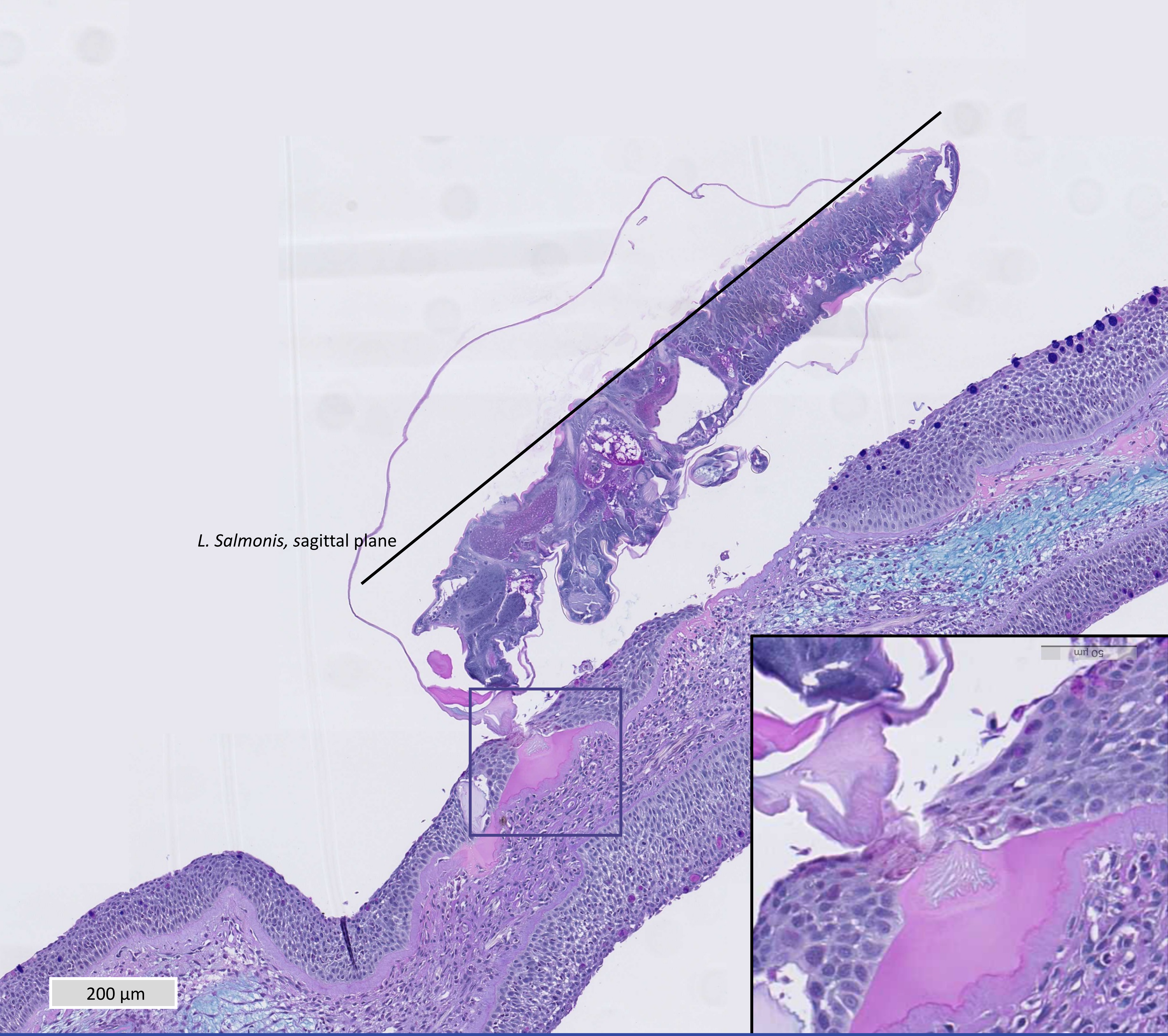

Atlantic salmon_5_41
Scaly skin_24_hpi

Section 2

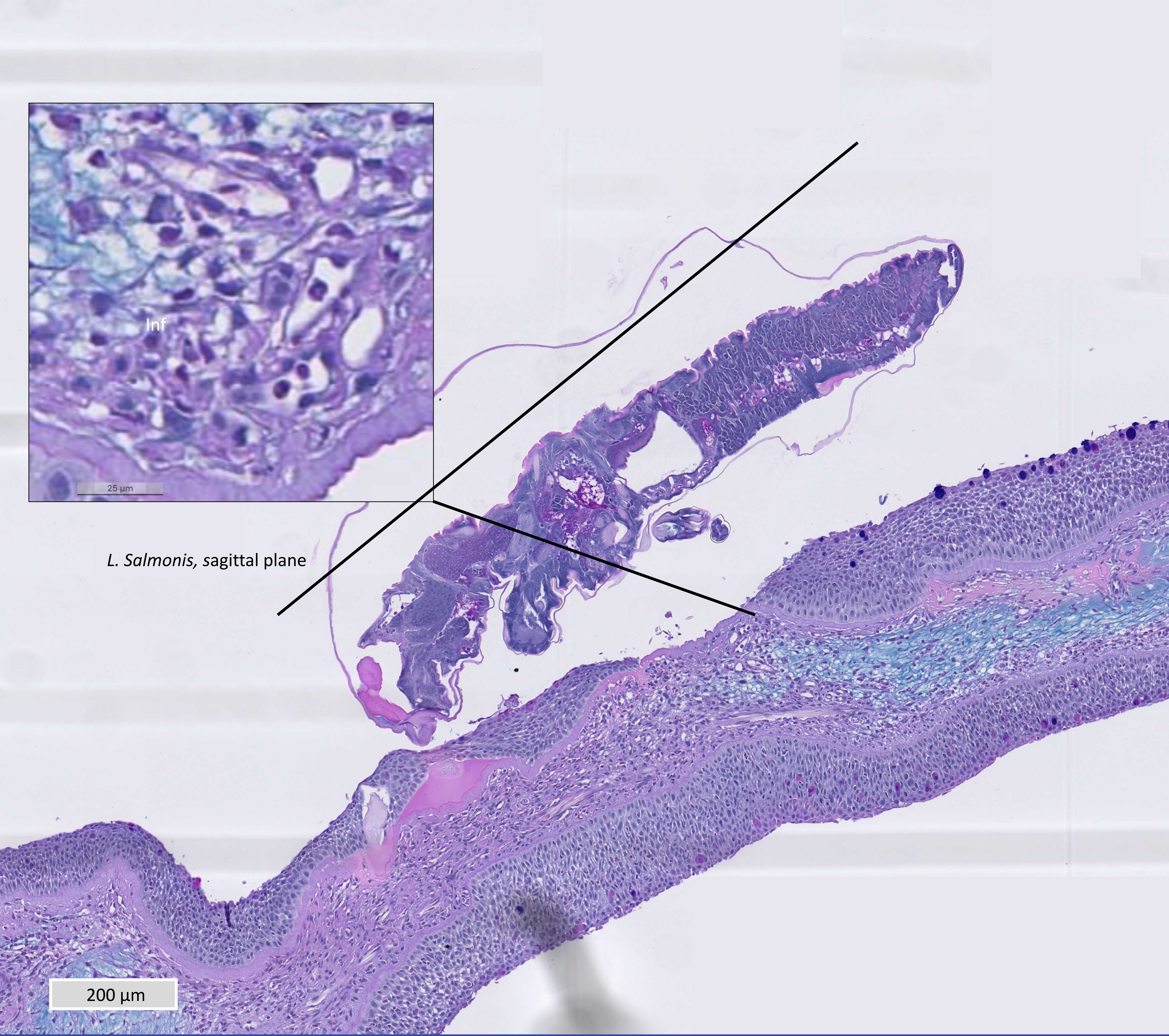

Atlantic salmon_5_41
Scaly skin_24_hpi

Section 3

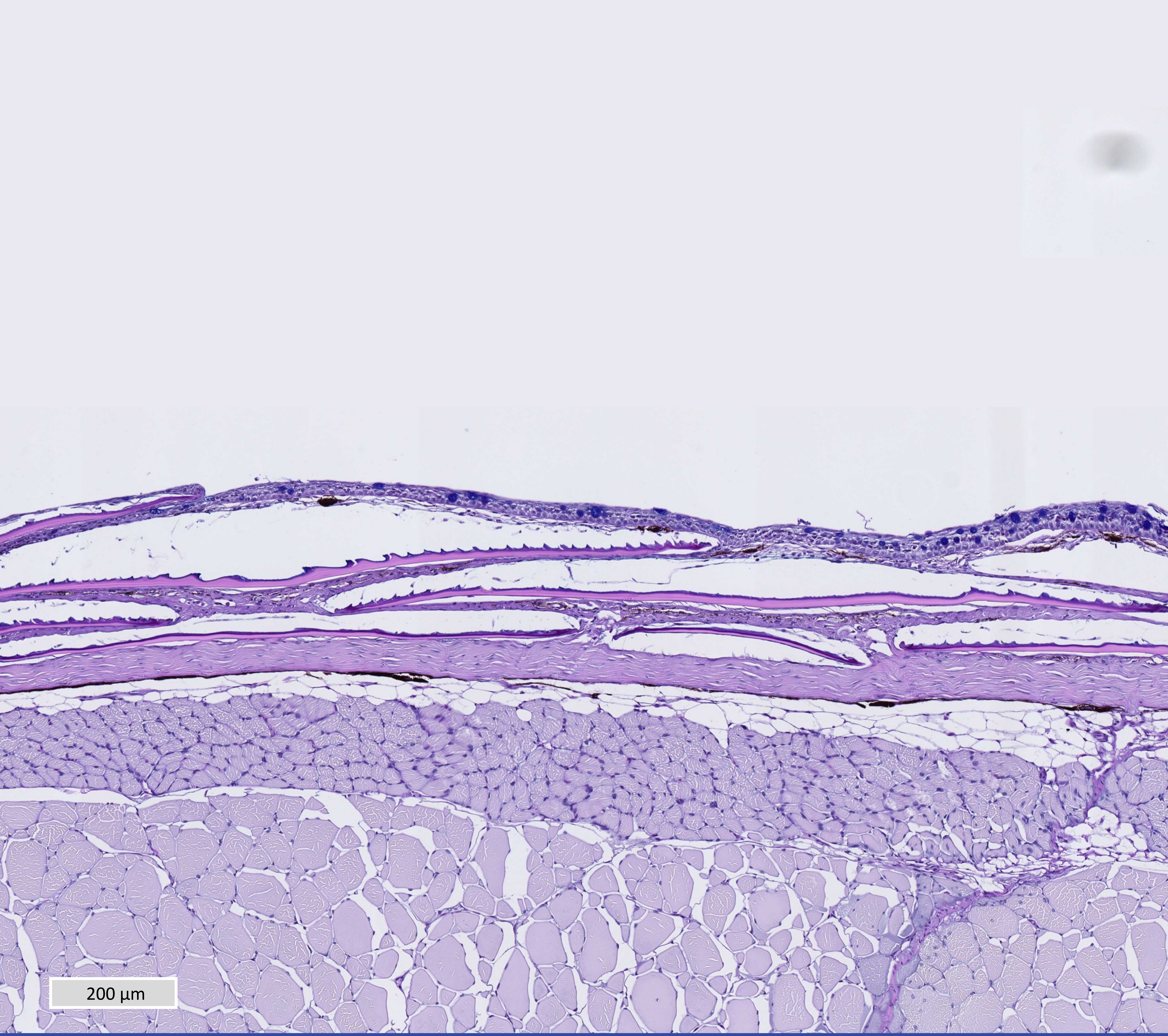

Atlantic salmon_6_45

Scaly skin_24_hpi

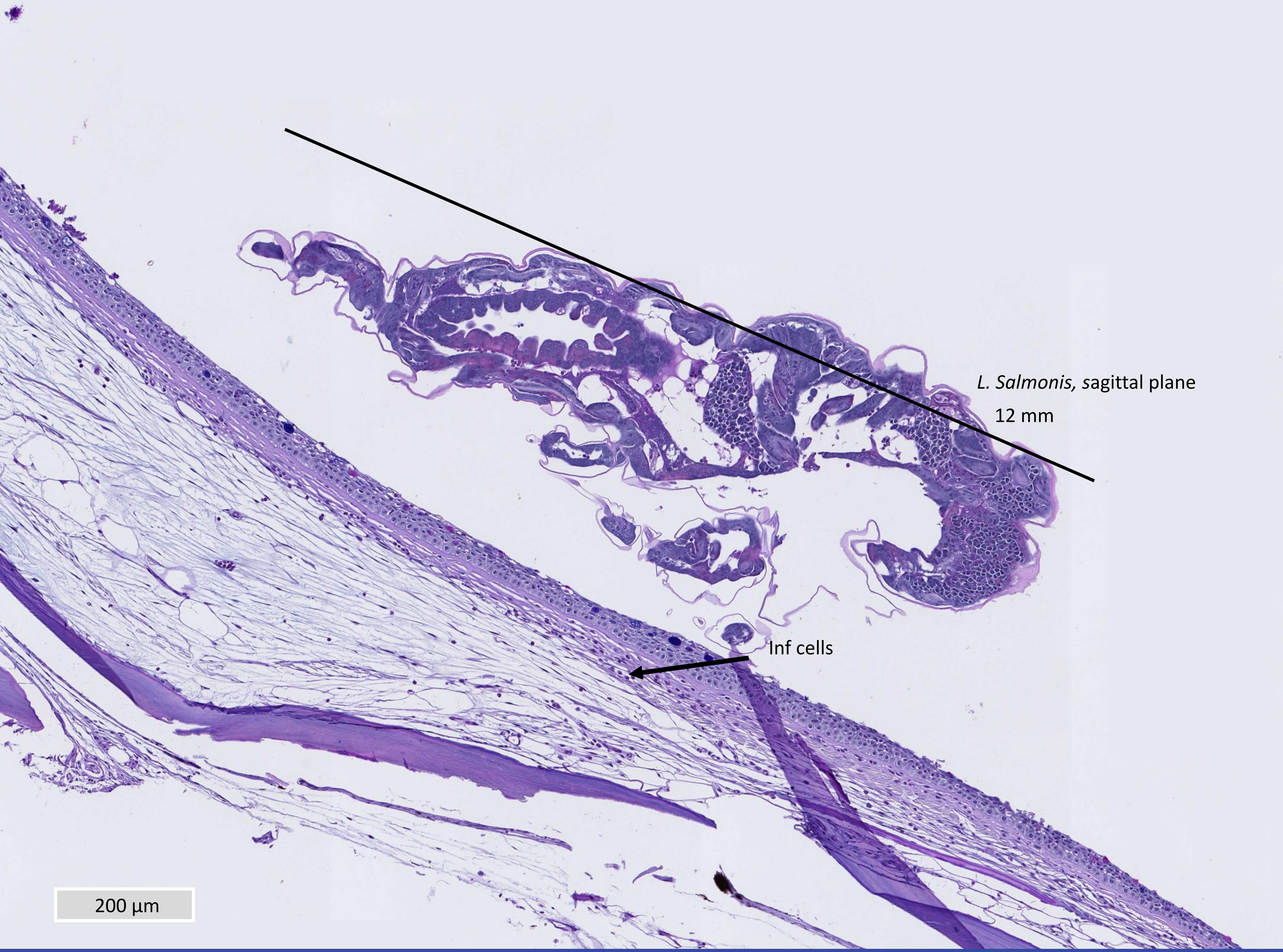

Atlantic salmon_7_74
Scaly skin_36_hpi

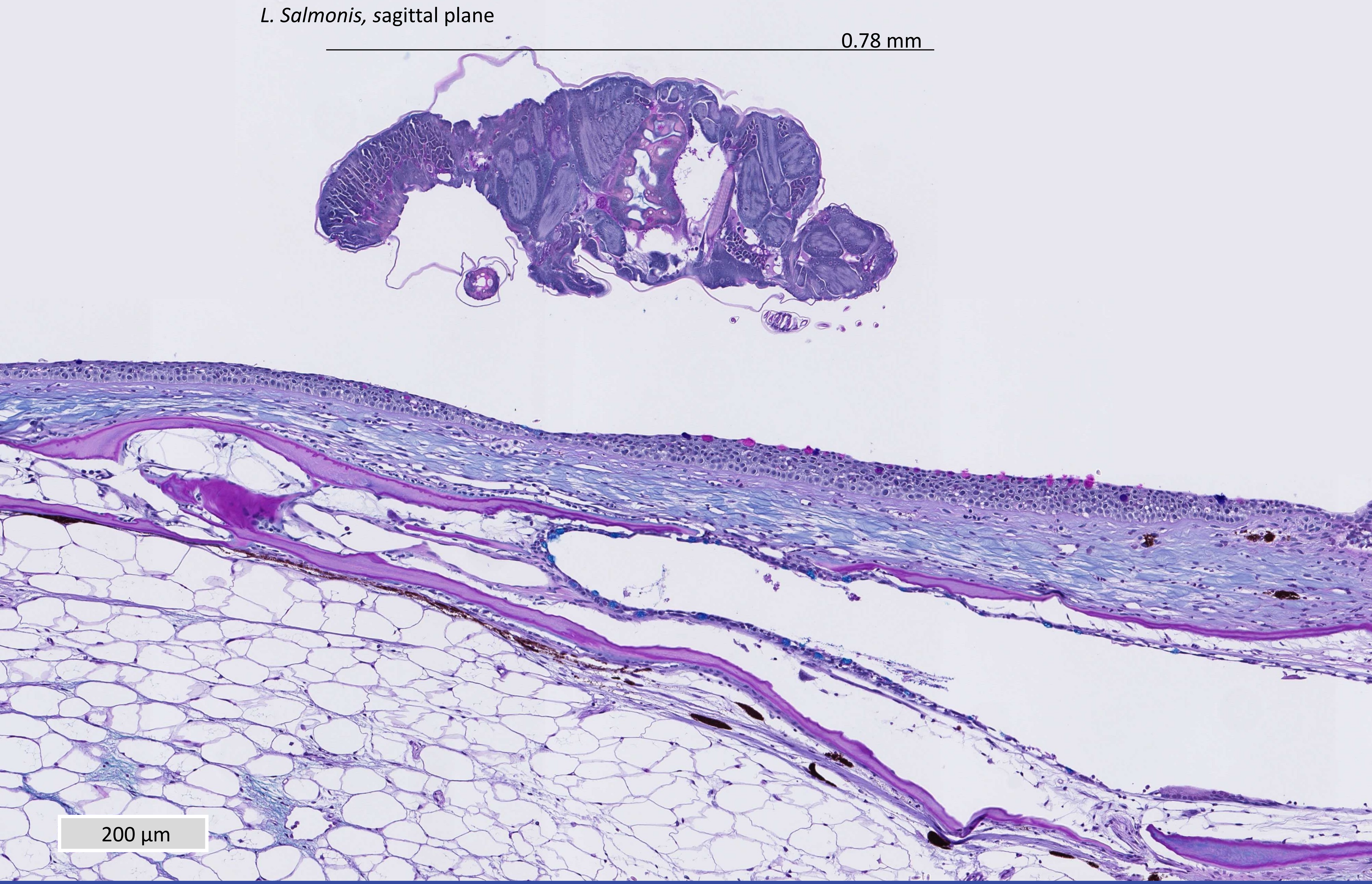

Atlantic salmon_8_77
Scaly skin_36_hpi

Section 1

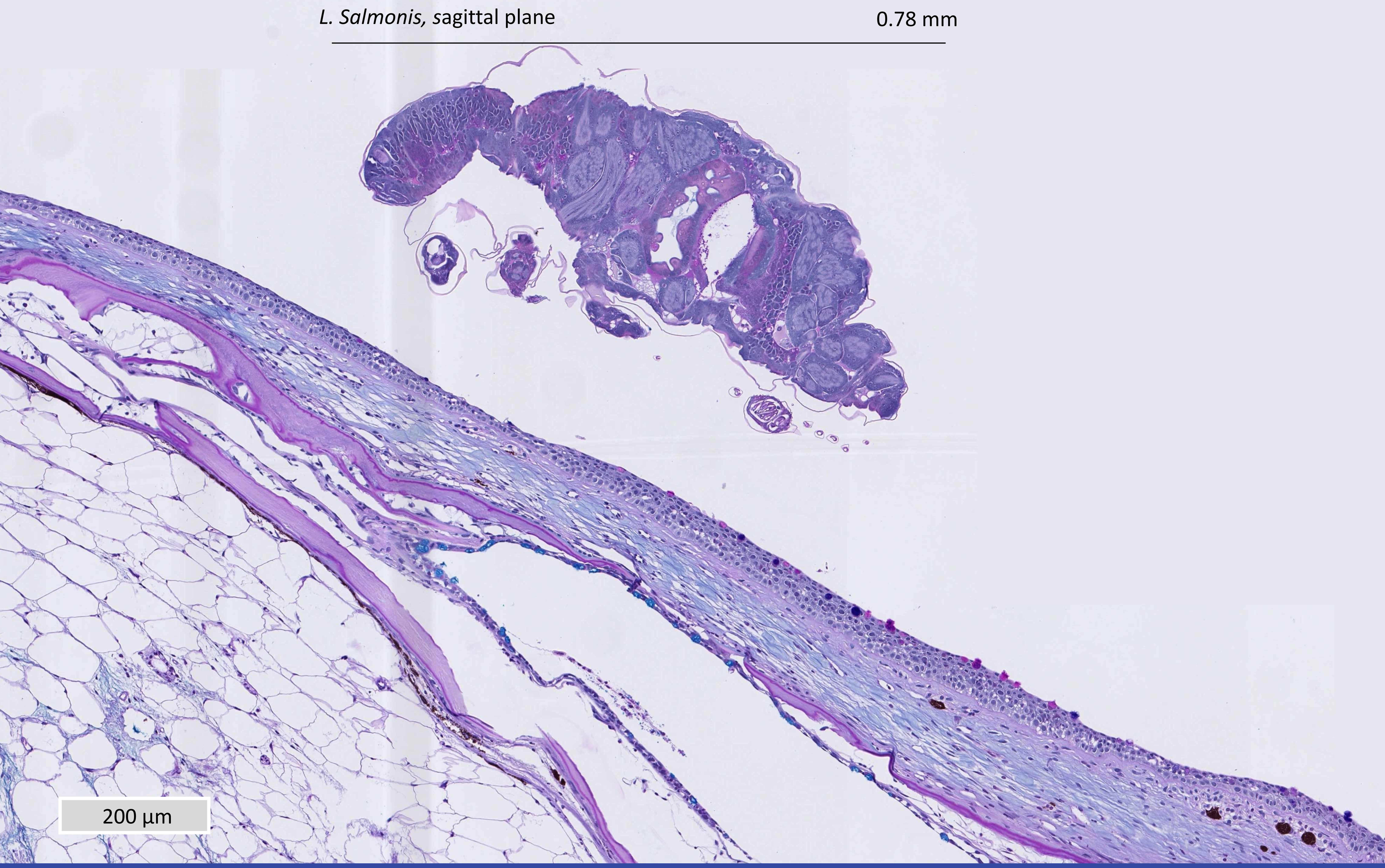

Atlantic salmon_8_77
Scaly skin_36_hpi

Section 2

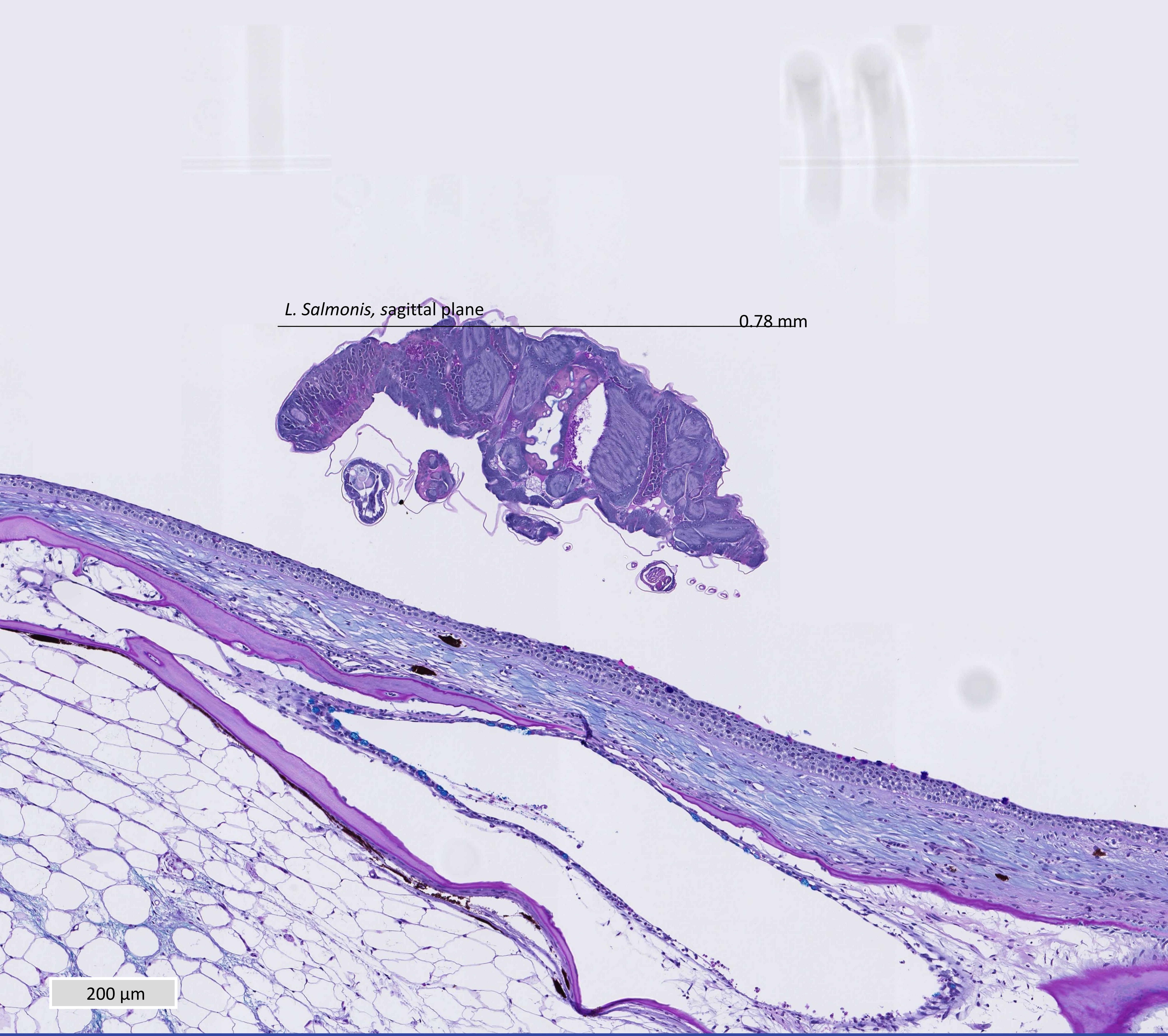

Atlantic salmon_8_77
Scaly skin_36_hpi

Section 3

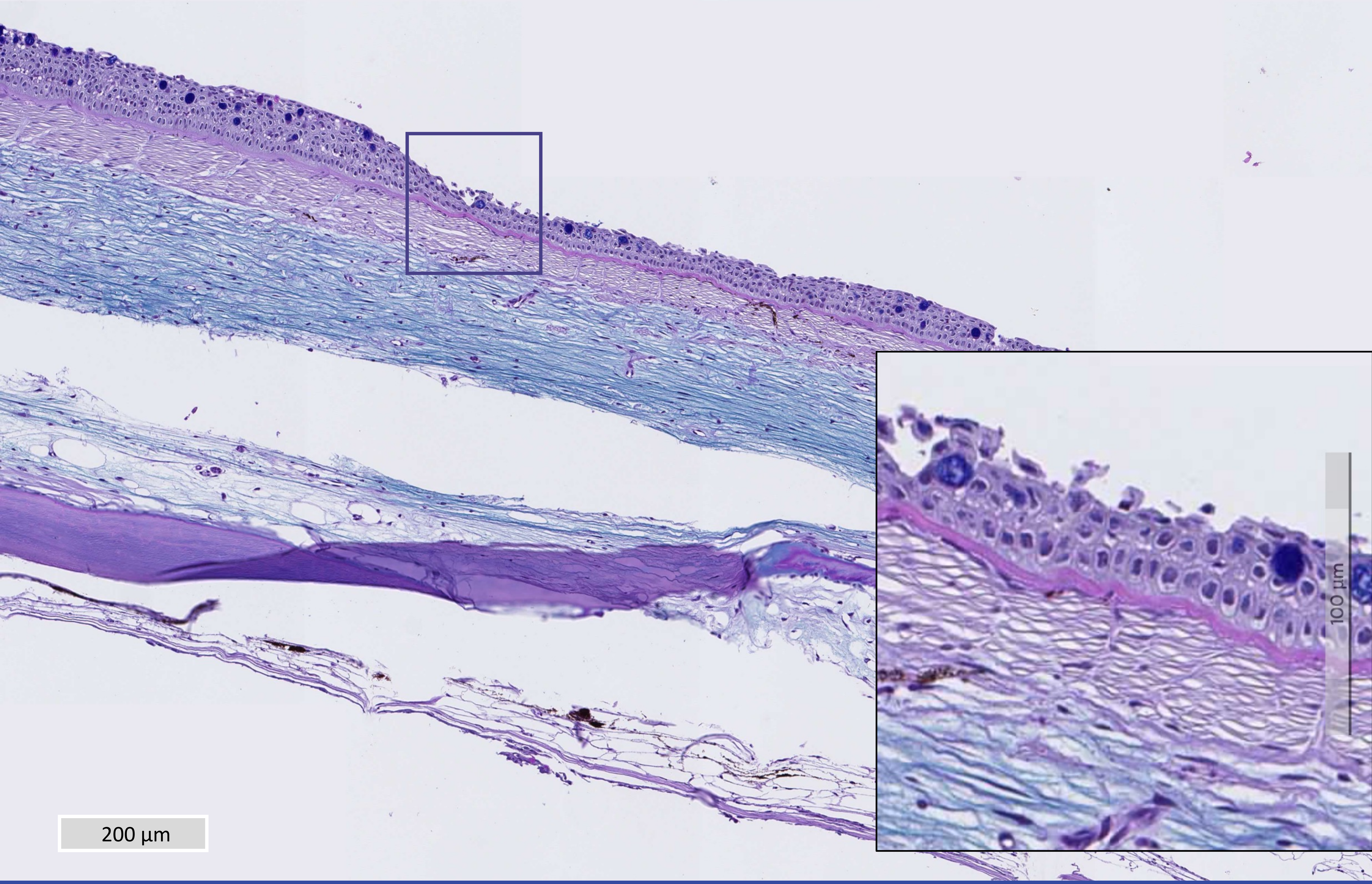

Atlantic salmon_9.1_80
Scaly skin_36_hpi

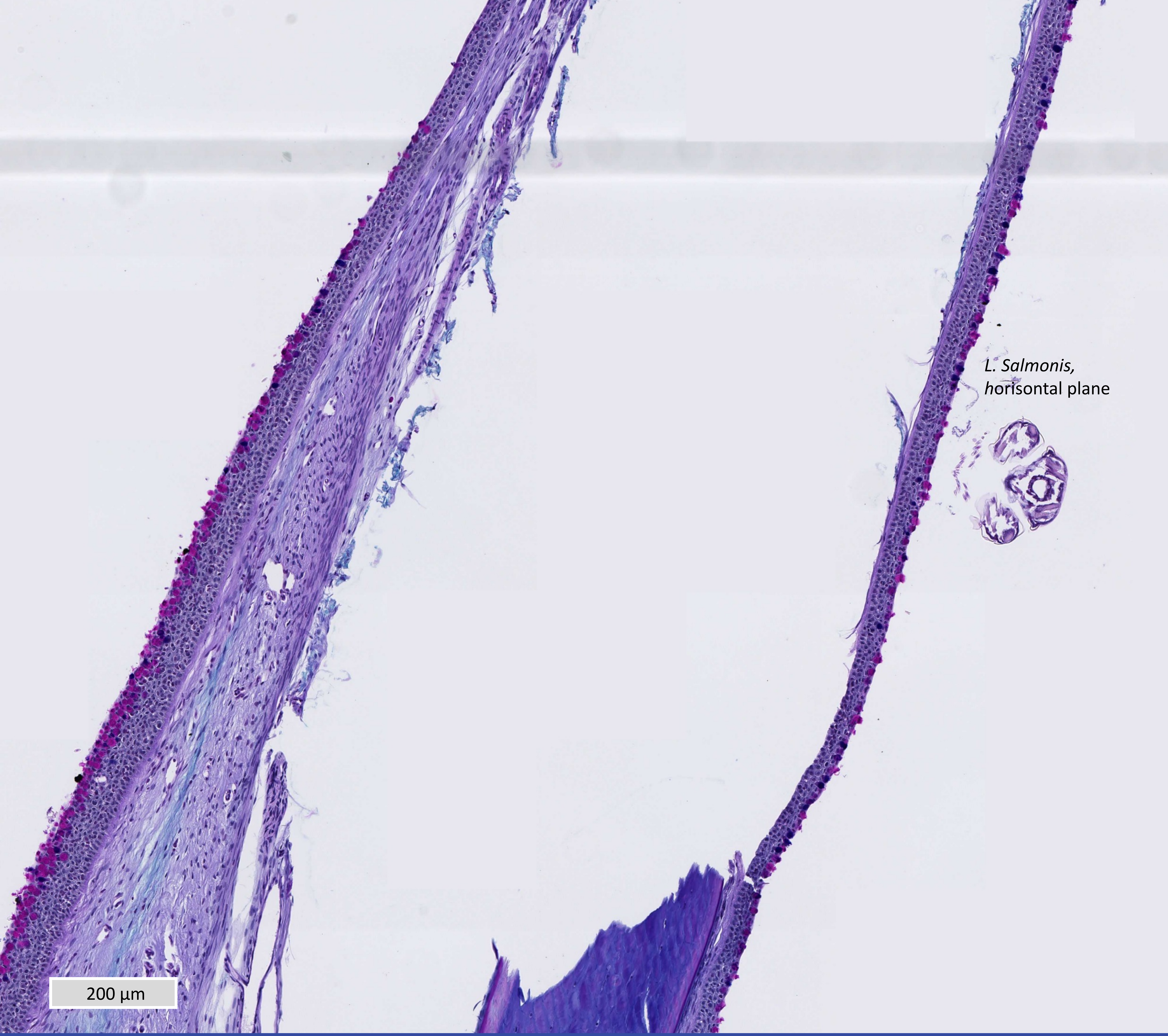

Atlantic salmon_9.2_80
Fin_36_hpi

Section 1

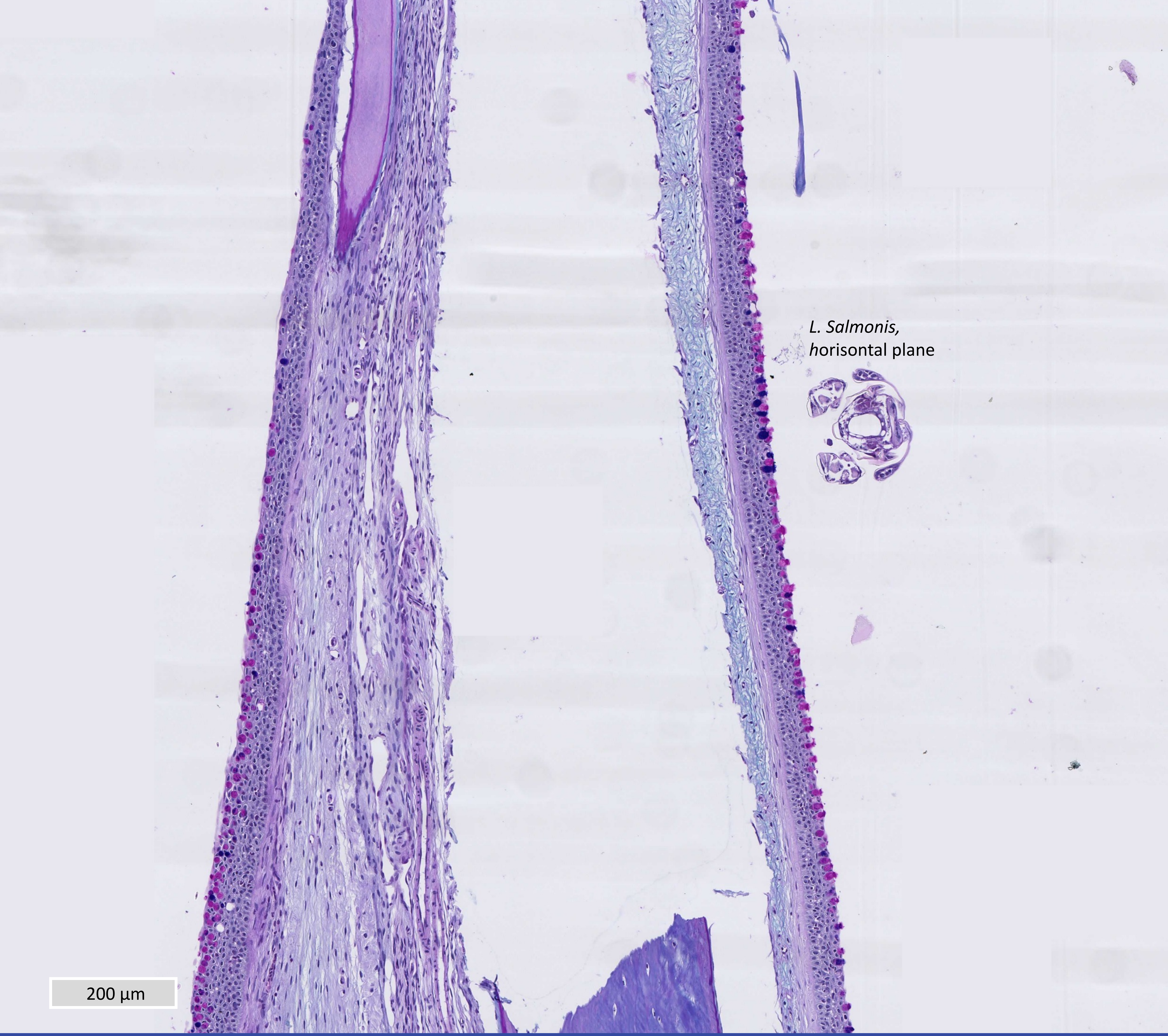

Atlantic salmon_9.2_80
Fin_36_hpi

Section 2

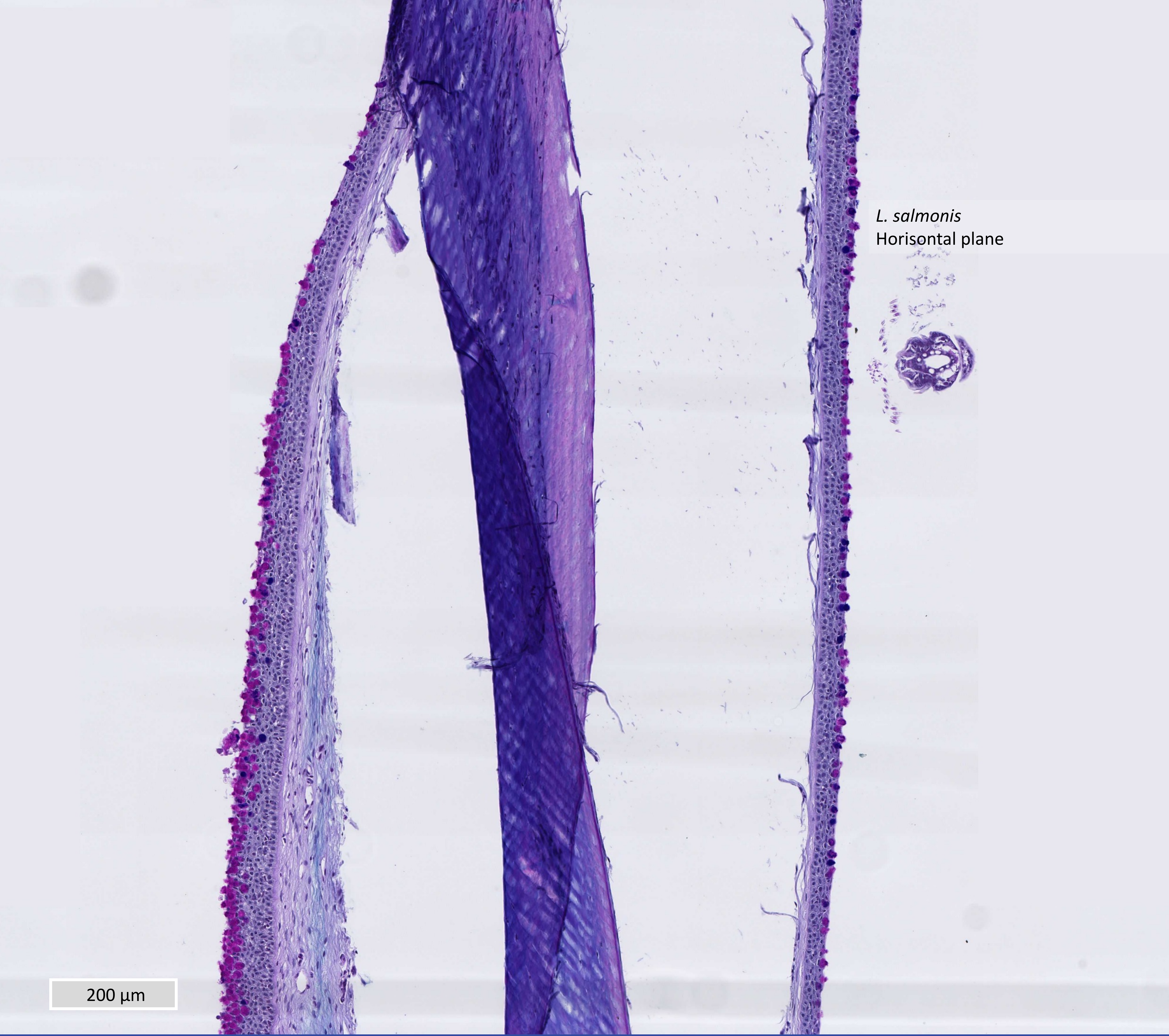

Atlantic salmon_9.2_80
Fin_36_hpi

Section 3

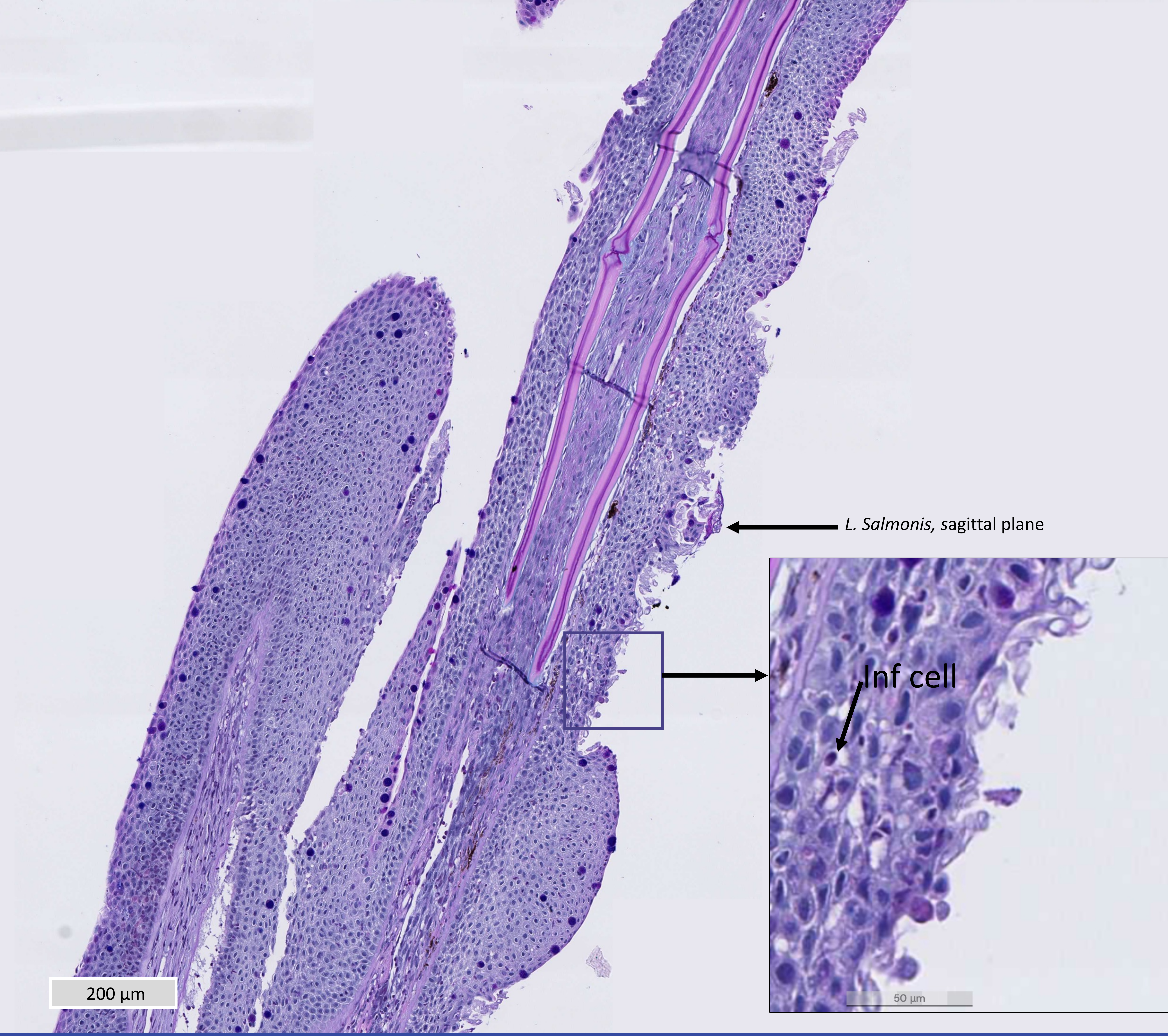

Atlantic salmon_10_114
Fin_48_hpi

Section 1

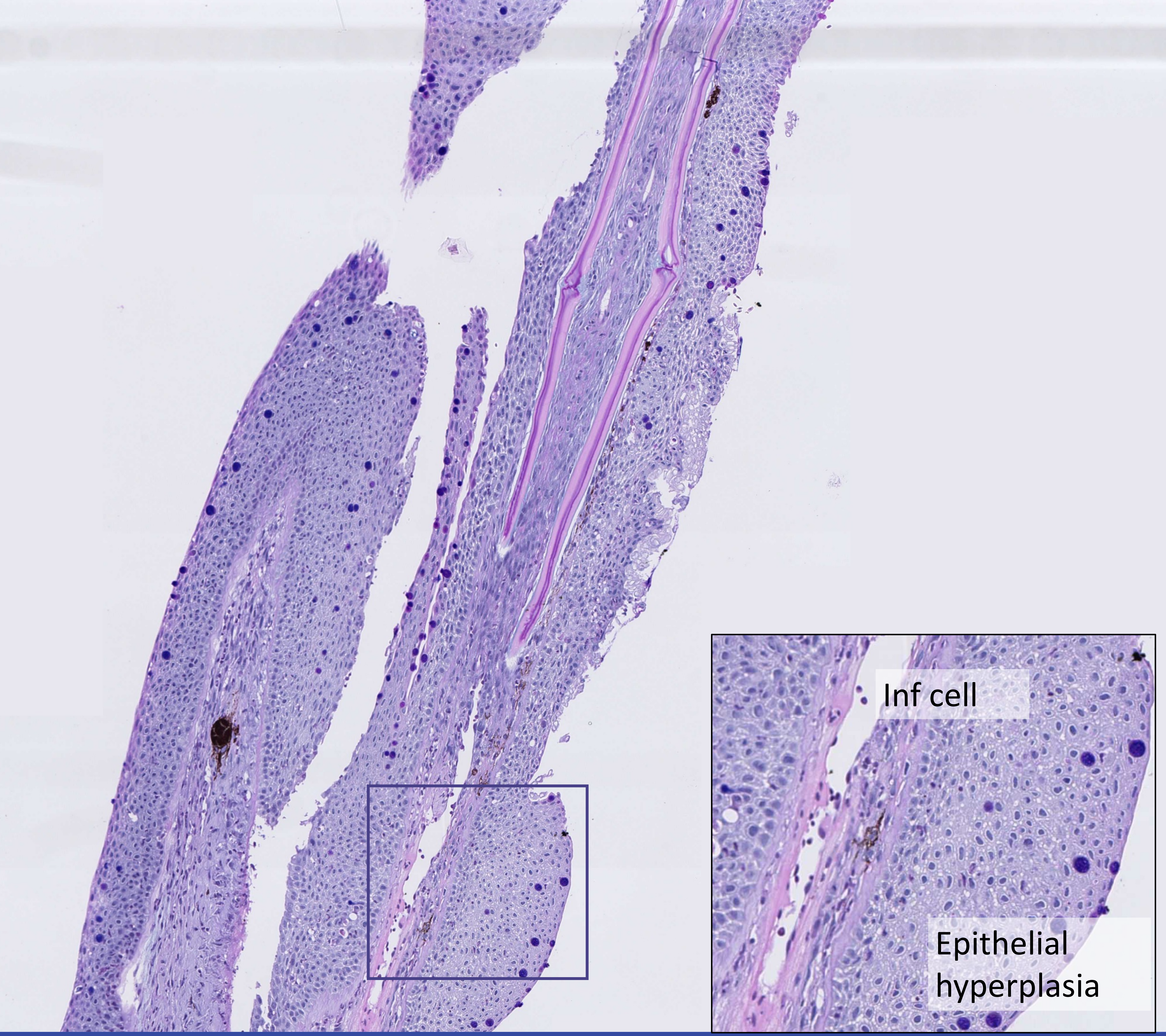

Atlantic salmon_10_114
Fin pelvic_48_hpi

Section 2

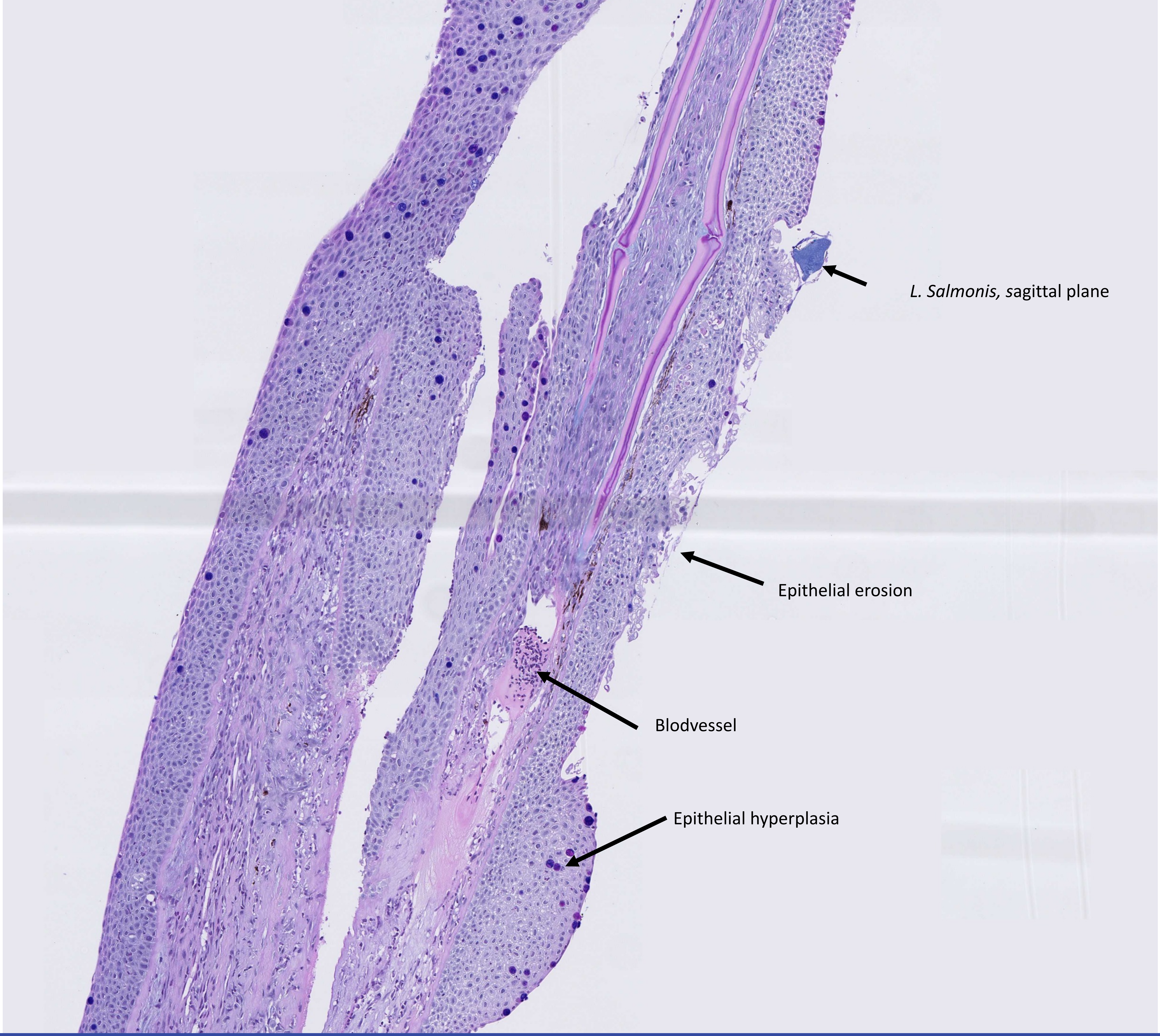

Atlantic salmon_10_114
Fin pelvic_48_hpi

Section 3

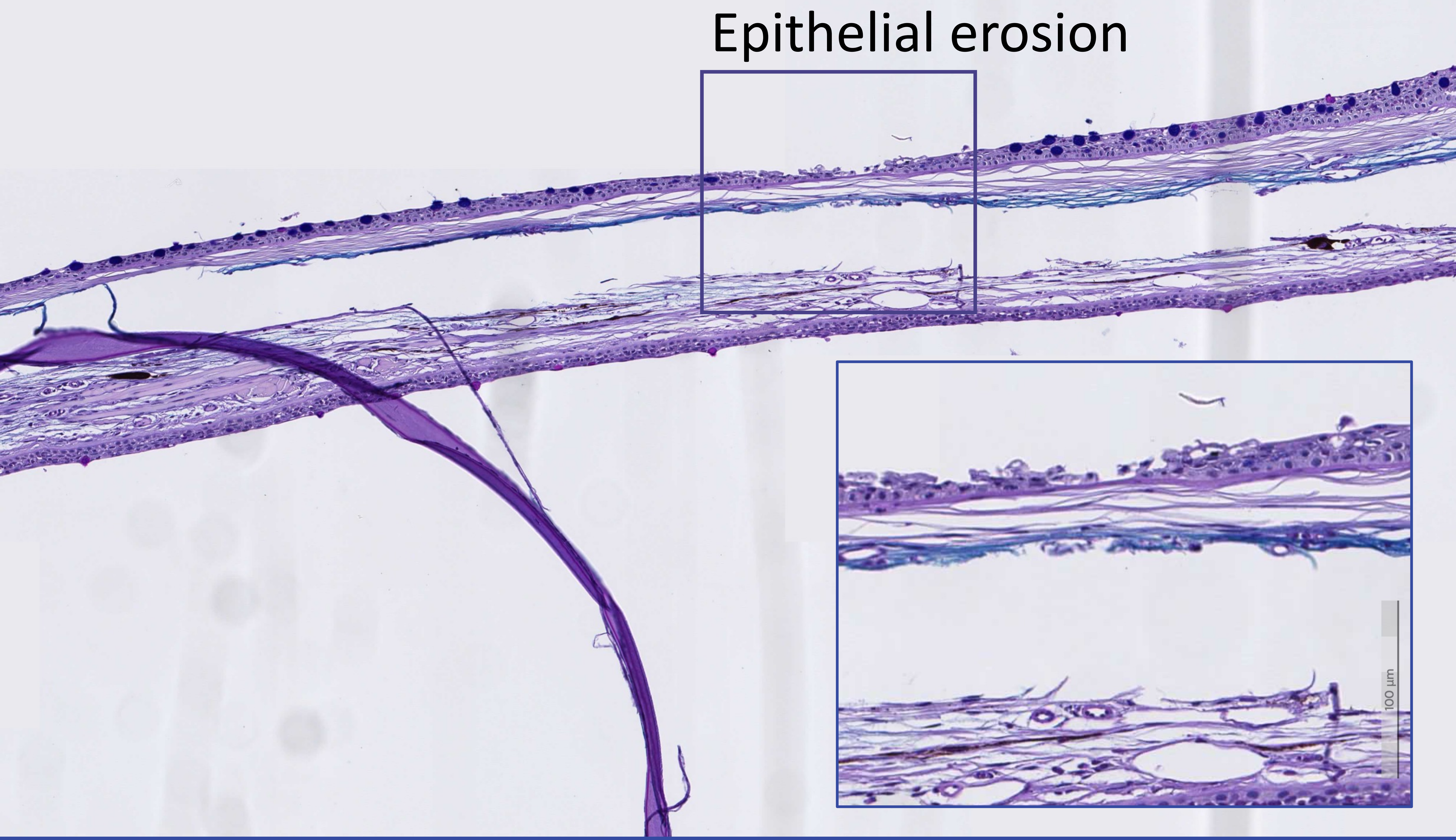

Atlantic salmon_11_116
Fin caudal_48_hpi

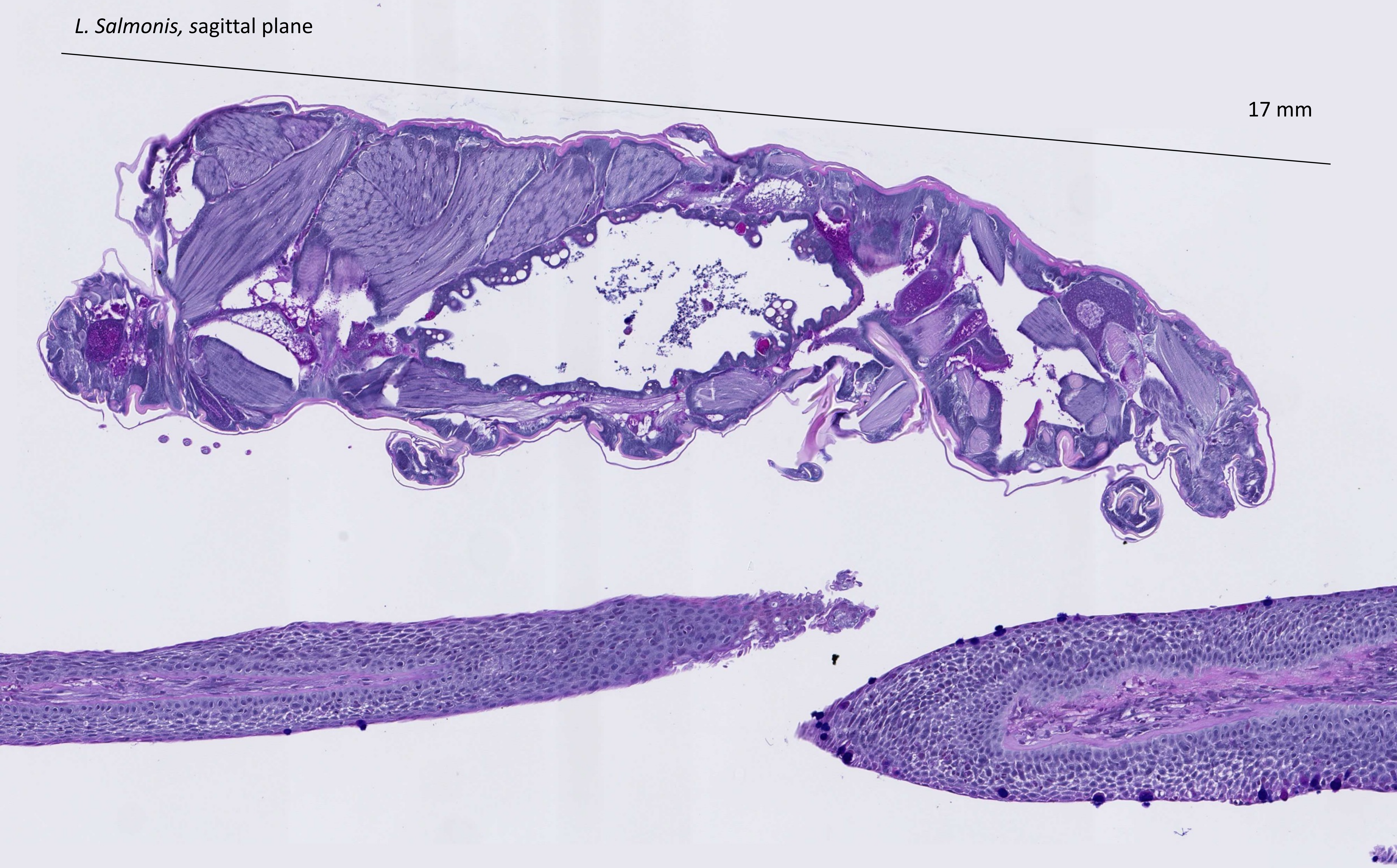

Atlantic salmon_12_145
Fin pelvic_60_hpi

Section 1

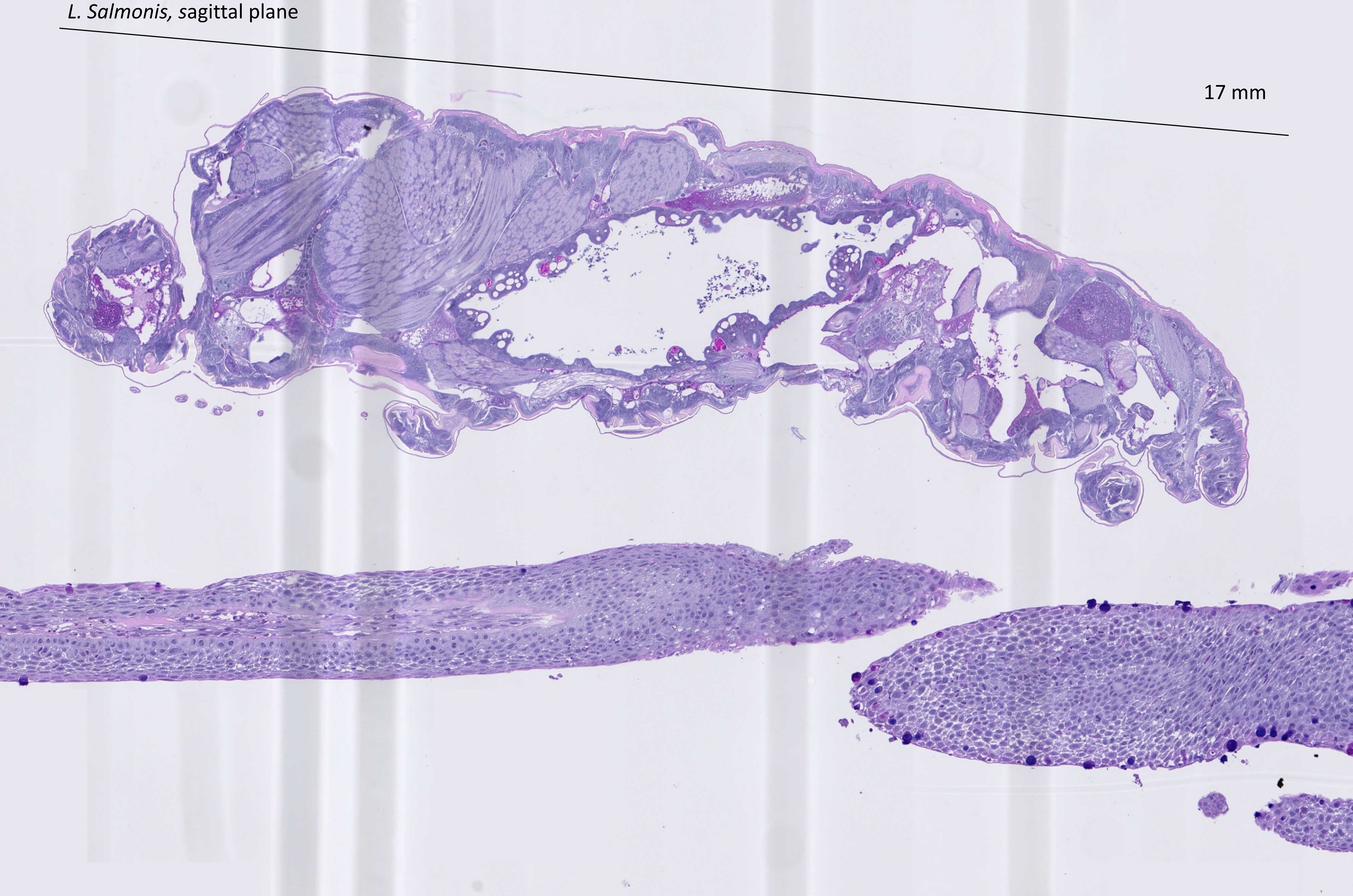

Atlantic salmon_12_145
Fin pelvic_60_hpi

Section 2

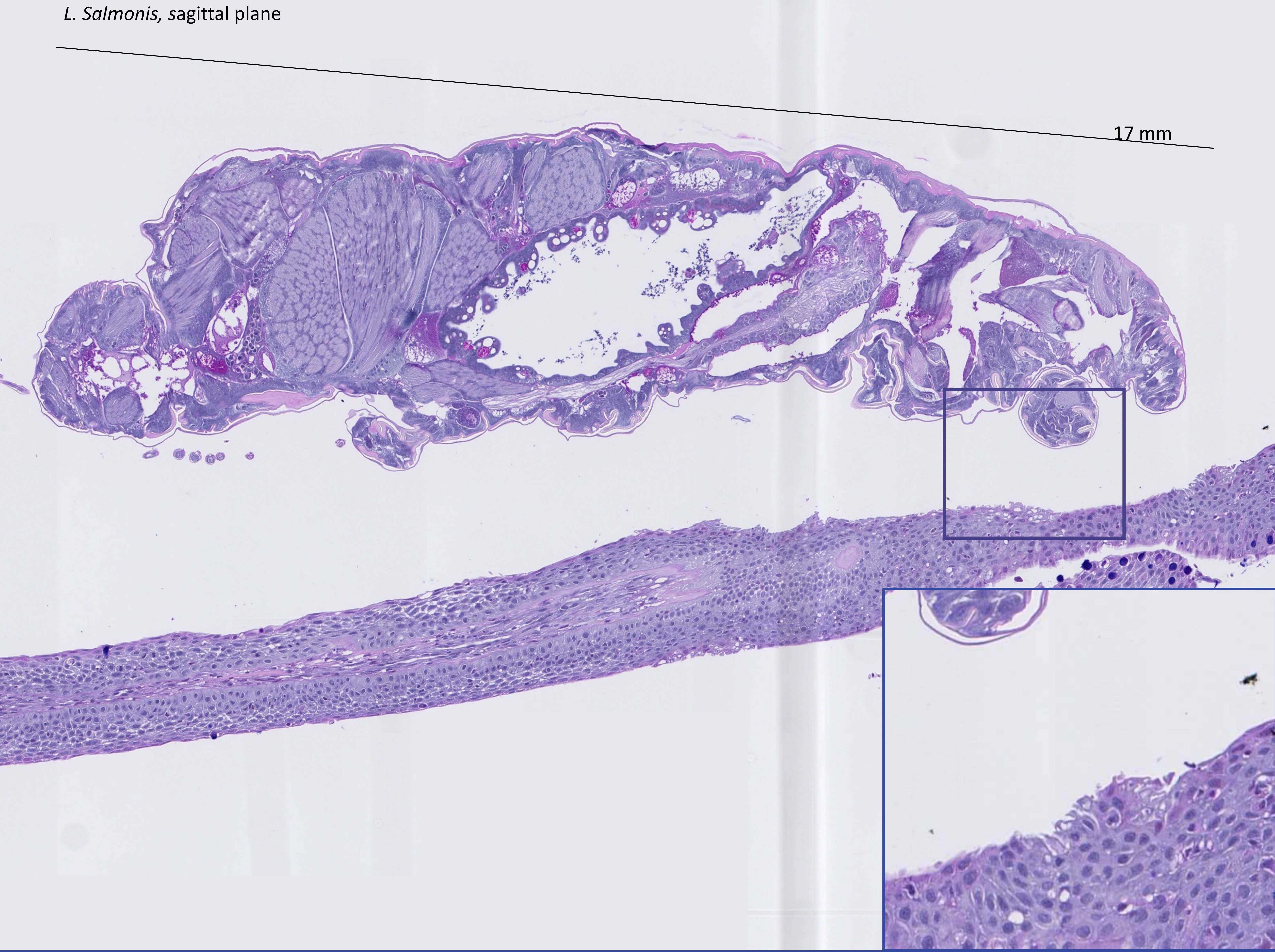

Atlantic salmon_12_145
Fin pelvic_60_hpi

Section 3

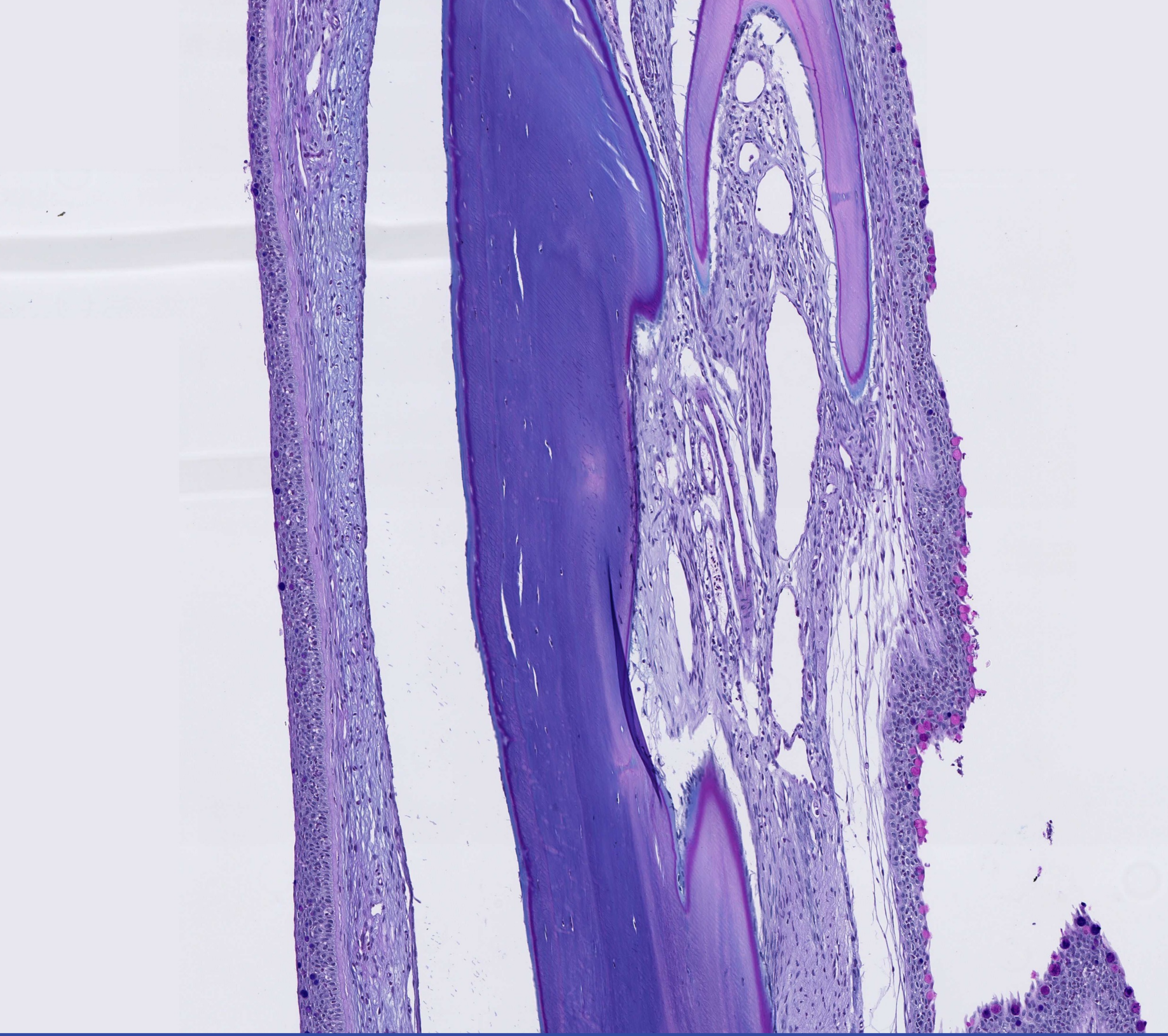

Atlantic salmon_13_148
Operculum_60_hpi

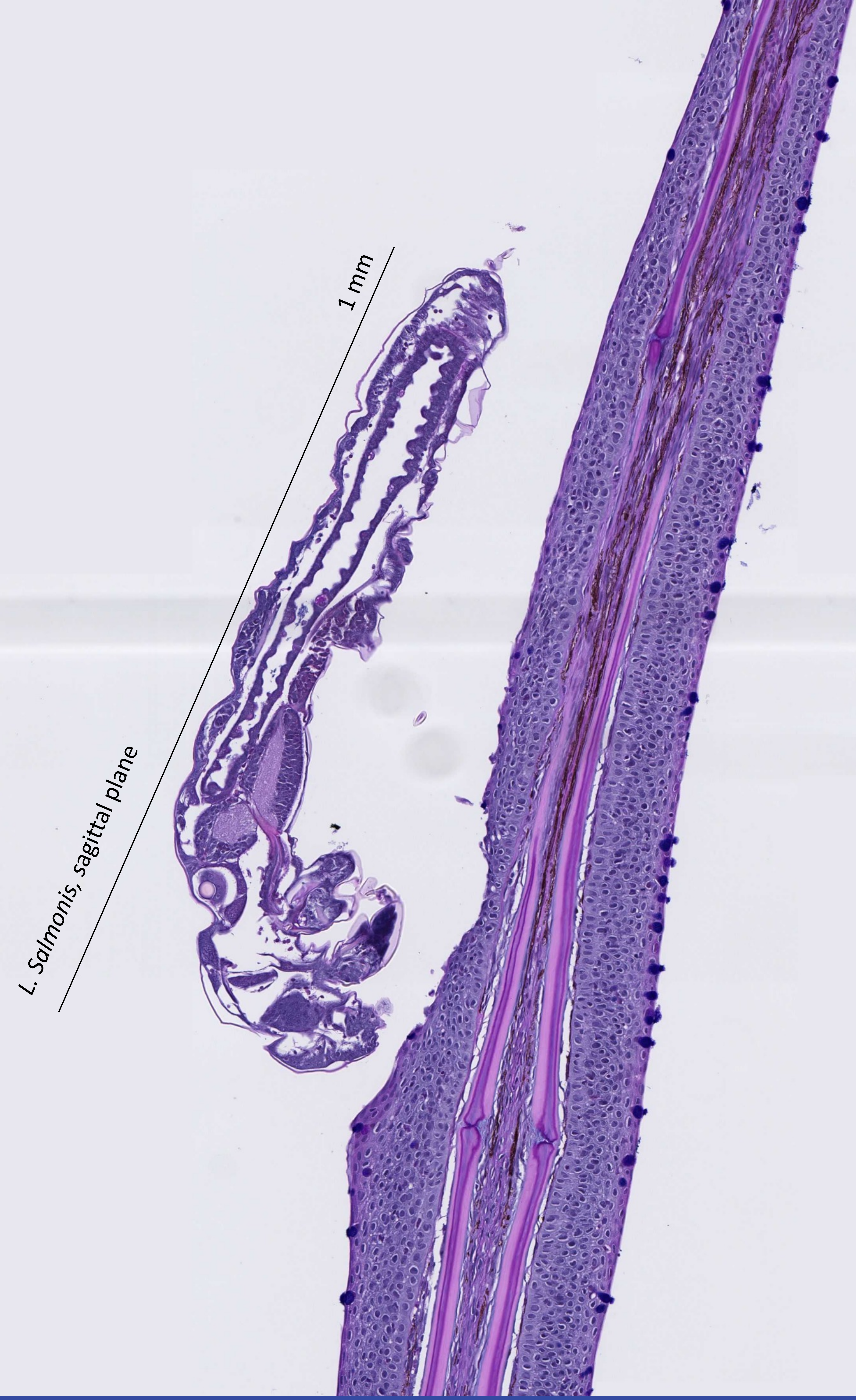

Atlantic salmon_14.1_160
Fin 168 hpi

Section 1

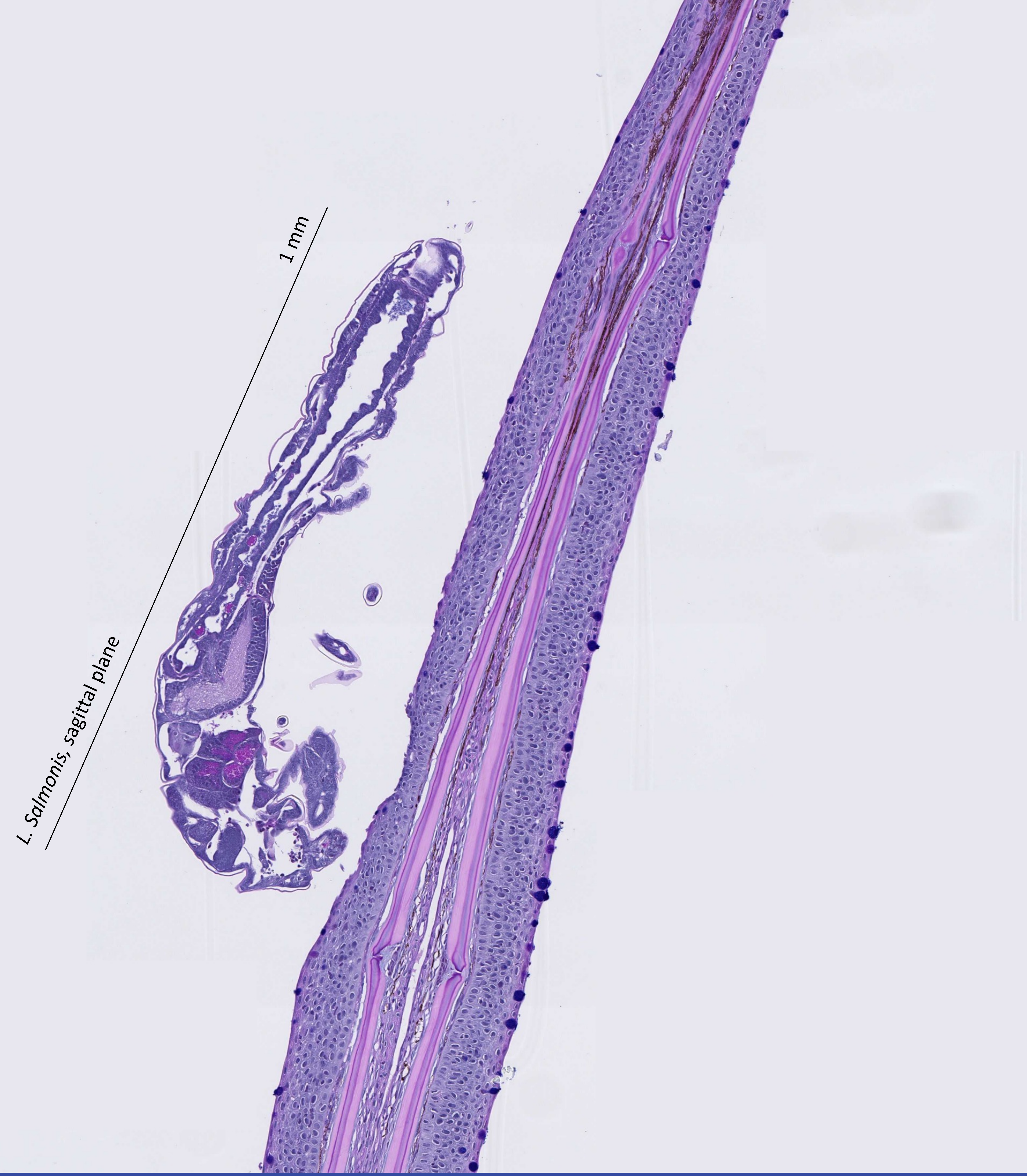

Atlantic salmon_14.1_160
Fin 168 hpi
Section 2

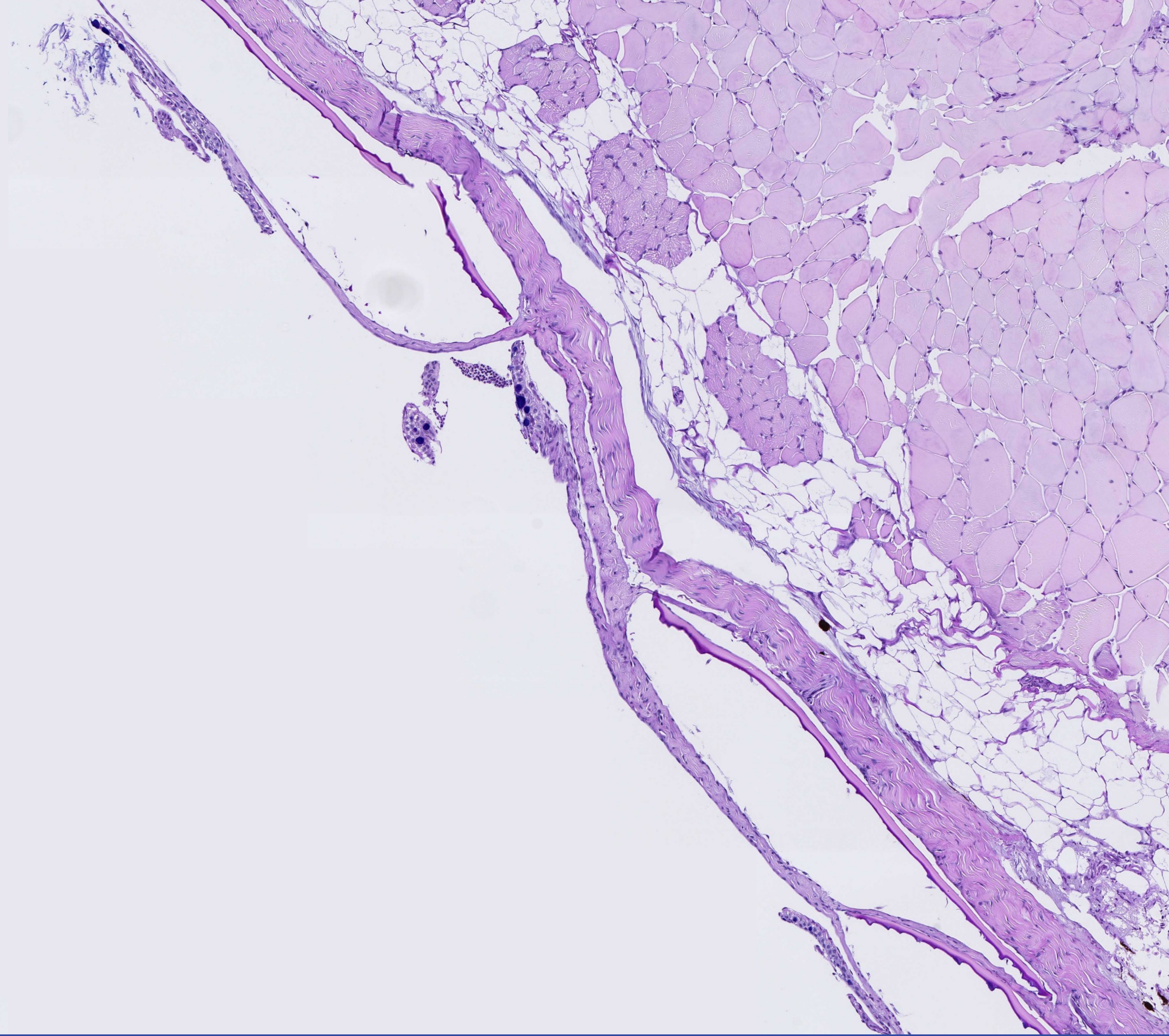

Atlantic salmon_14.2_160
Scaly skin 168 hpi

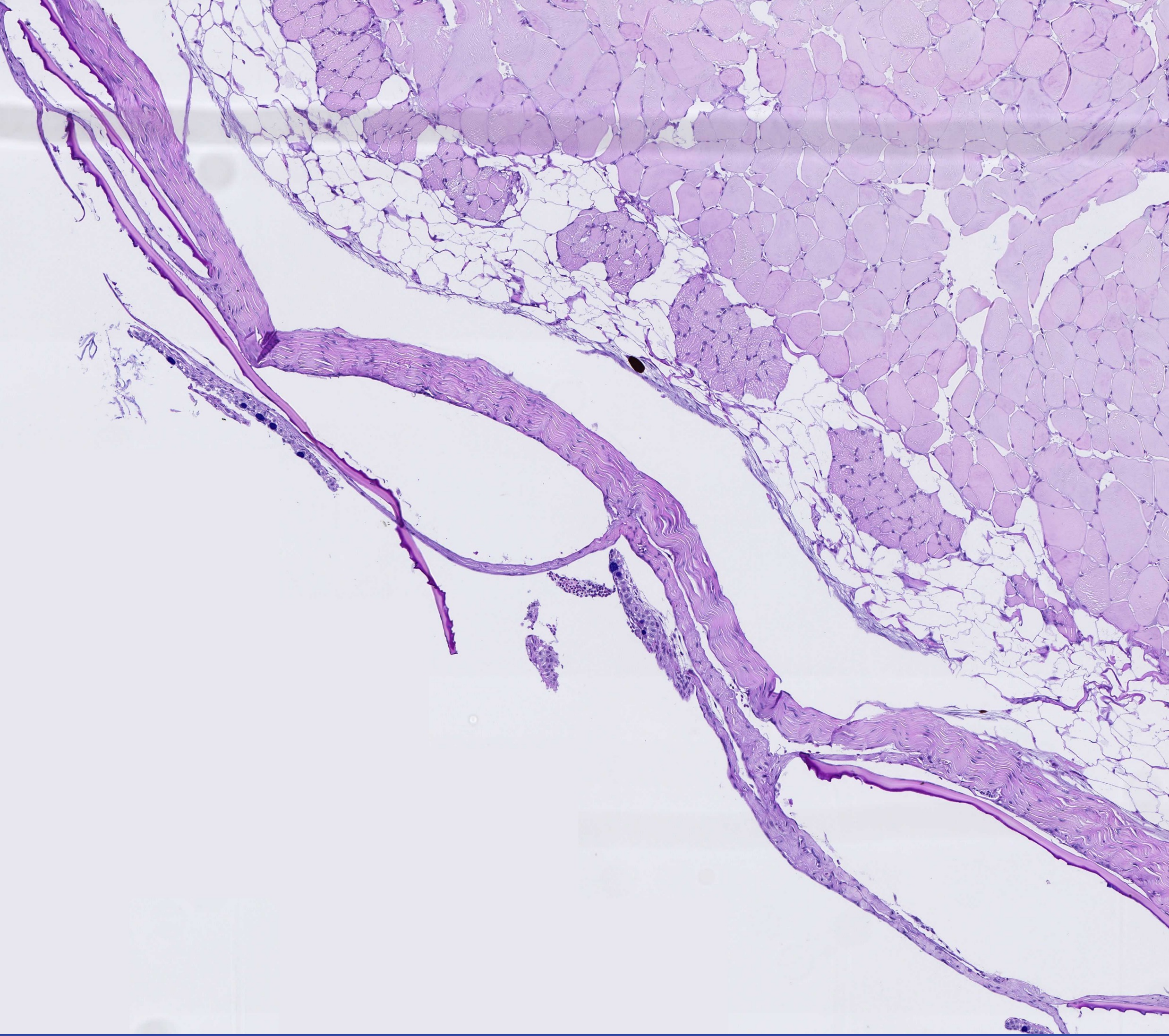

Atlantic salmon X 160

Scaly skin 168 hpi

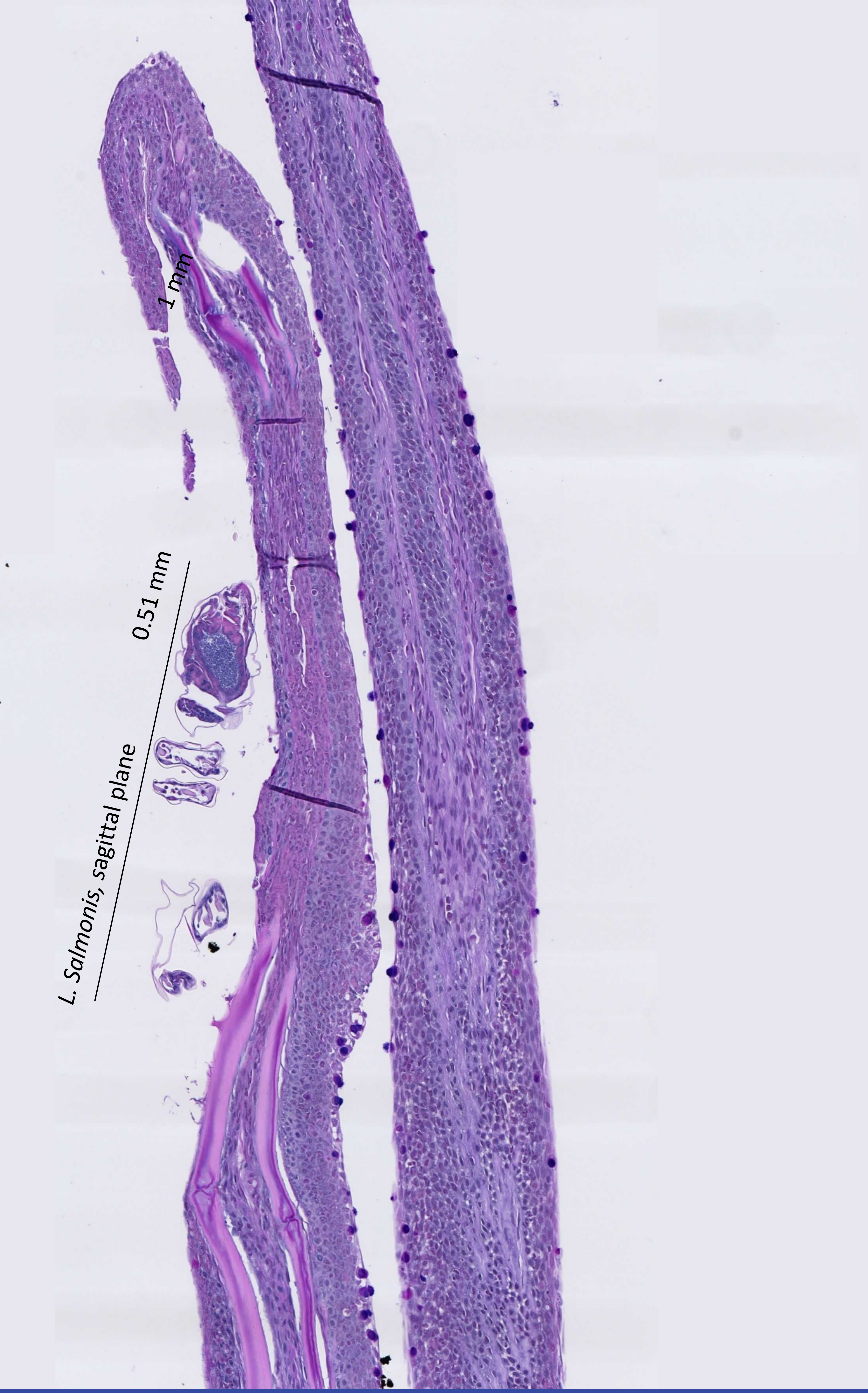

Atlantic salmon_15_163
Fin 168 hpi

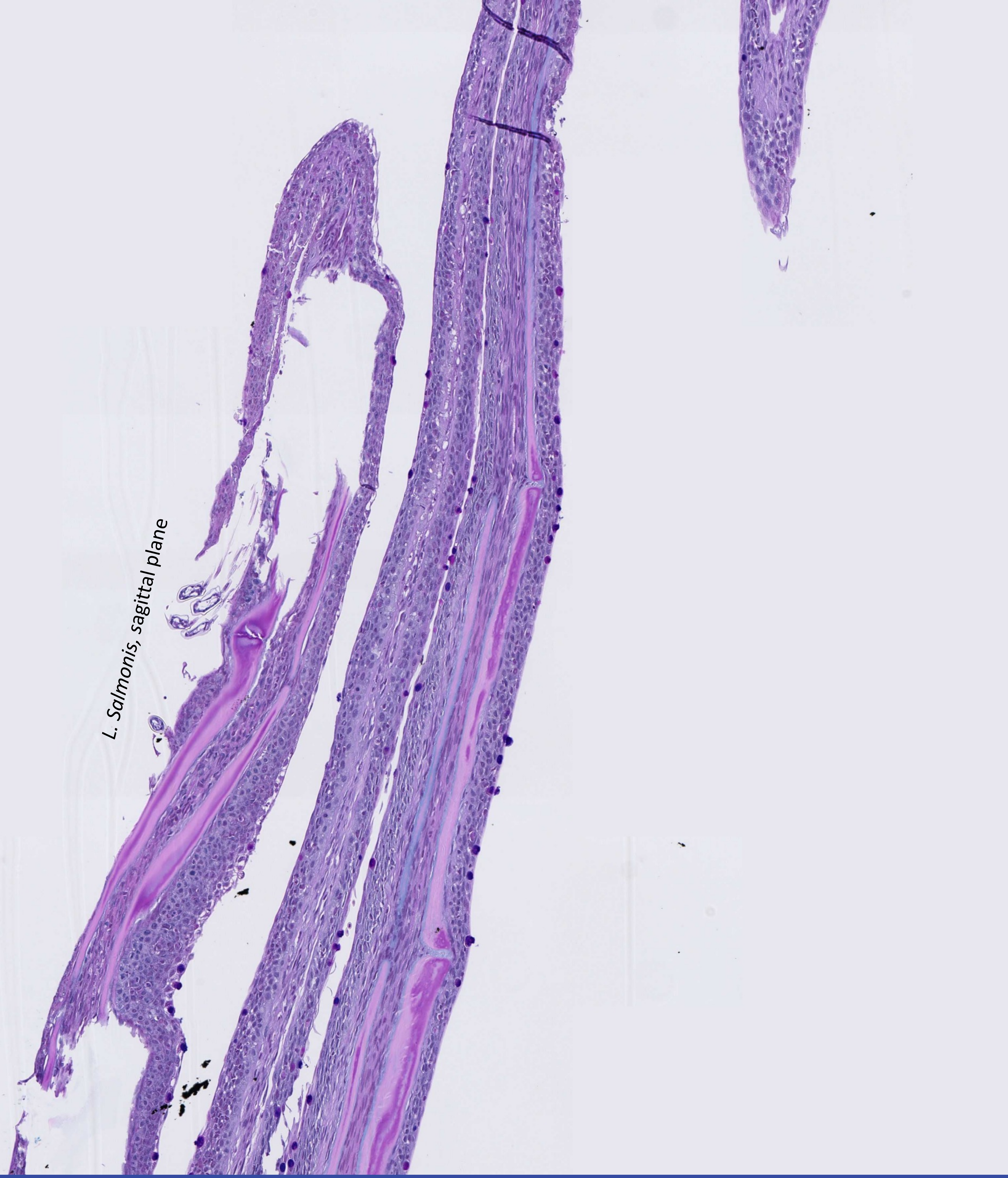

Atlantic salmon_15_163
Fin 168 hpi

Atlantic salmon, AB / PASc

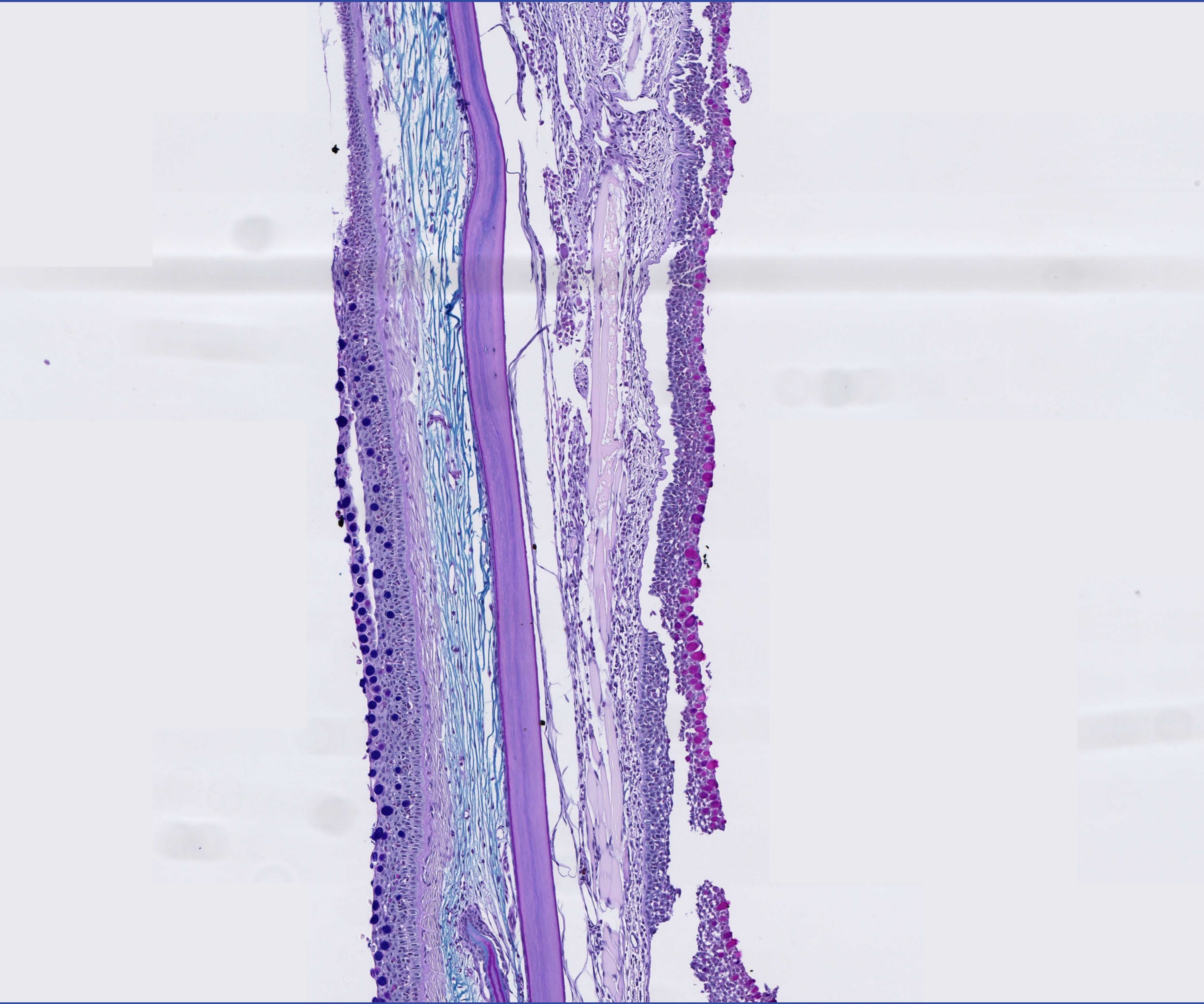

Atlantic salmon_15_163
Skin 168 hpi

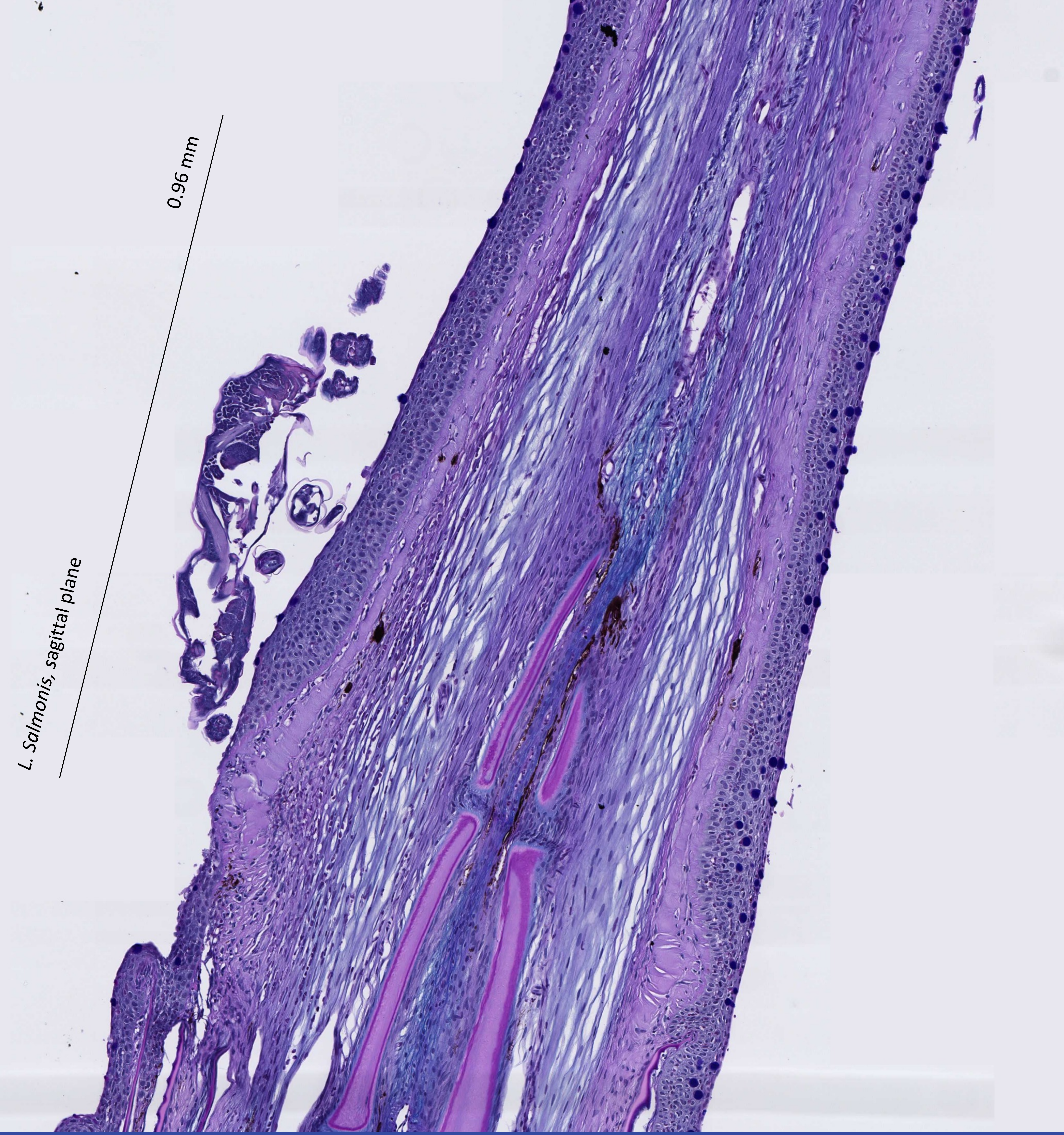

Atlantic salmon_16_167
Fin_168_hpi

Section 1

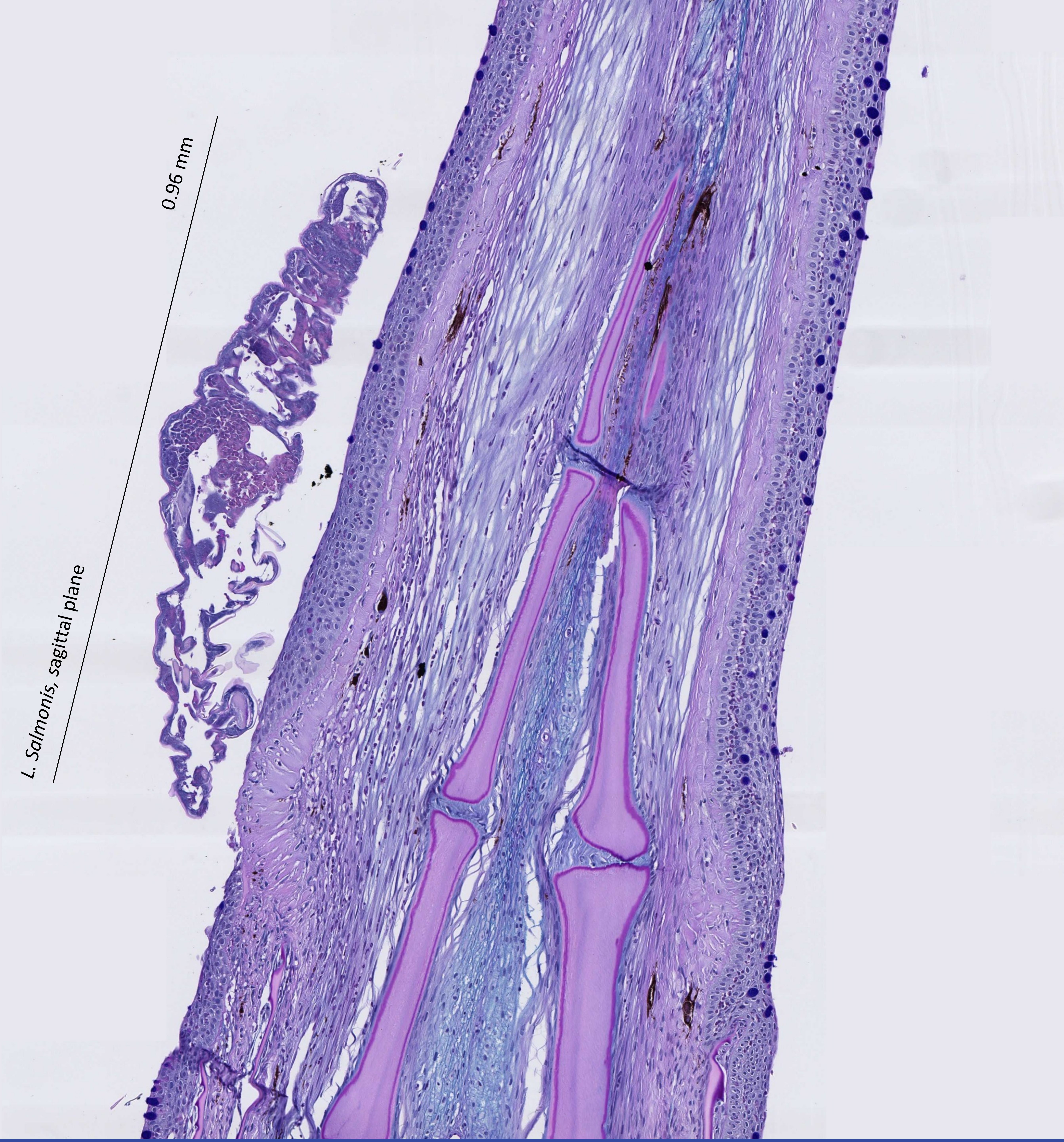

Atlantic salmon_16_167
Fin_168_hpi

Section 2

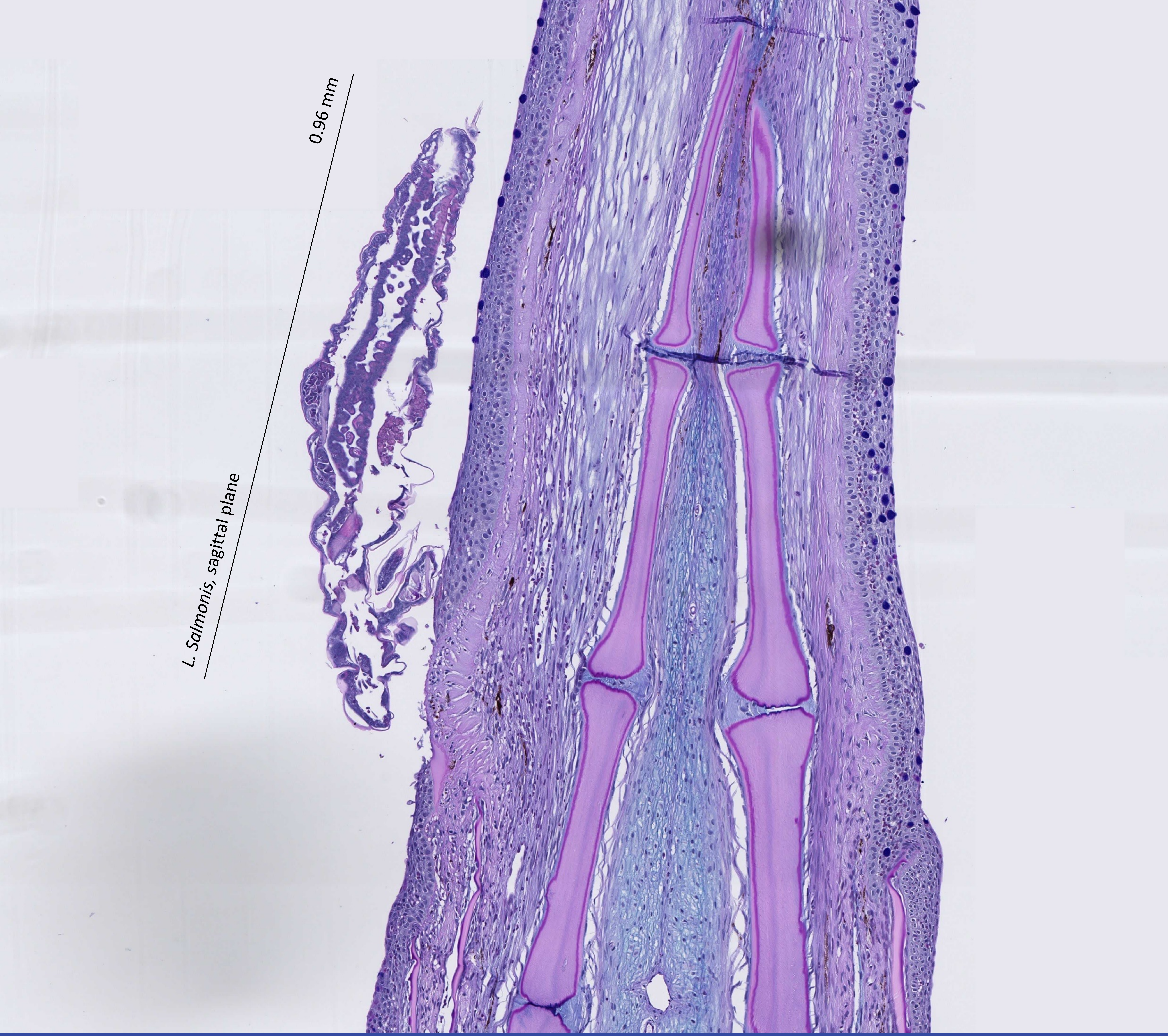

Atlantic salmon_16_167
Fin_168_hpi

Section 3

Spatial transcriptomic slide with coho salmon fin and *L.salmonis,* in the following order:

Frame 1 – coho salmon

Frame 2 – coho salmon

Frame 3 – Atlantic salmon

Frame 4 – Atlantic salmon

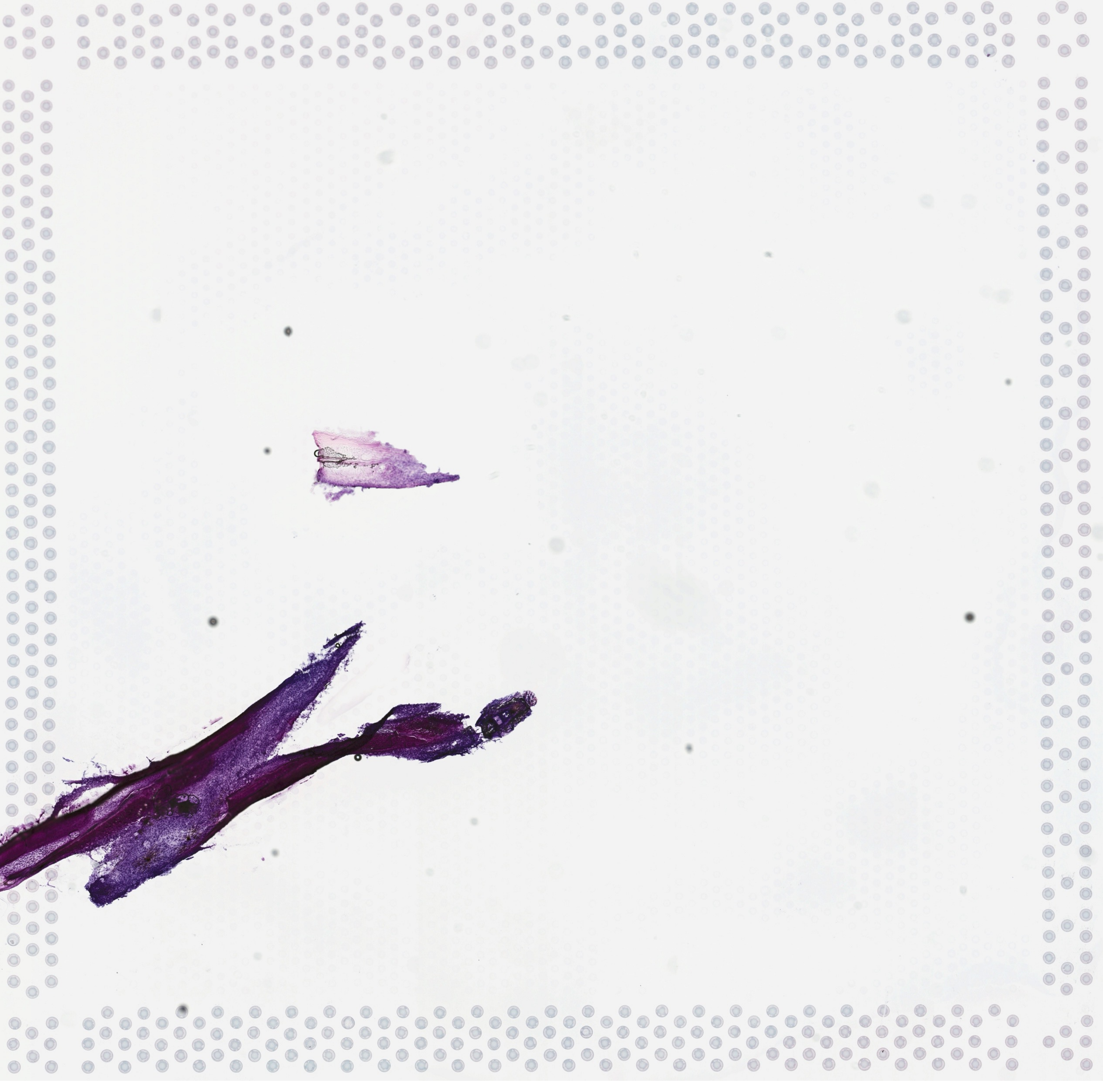

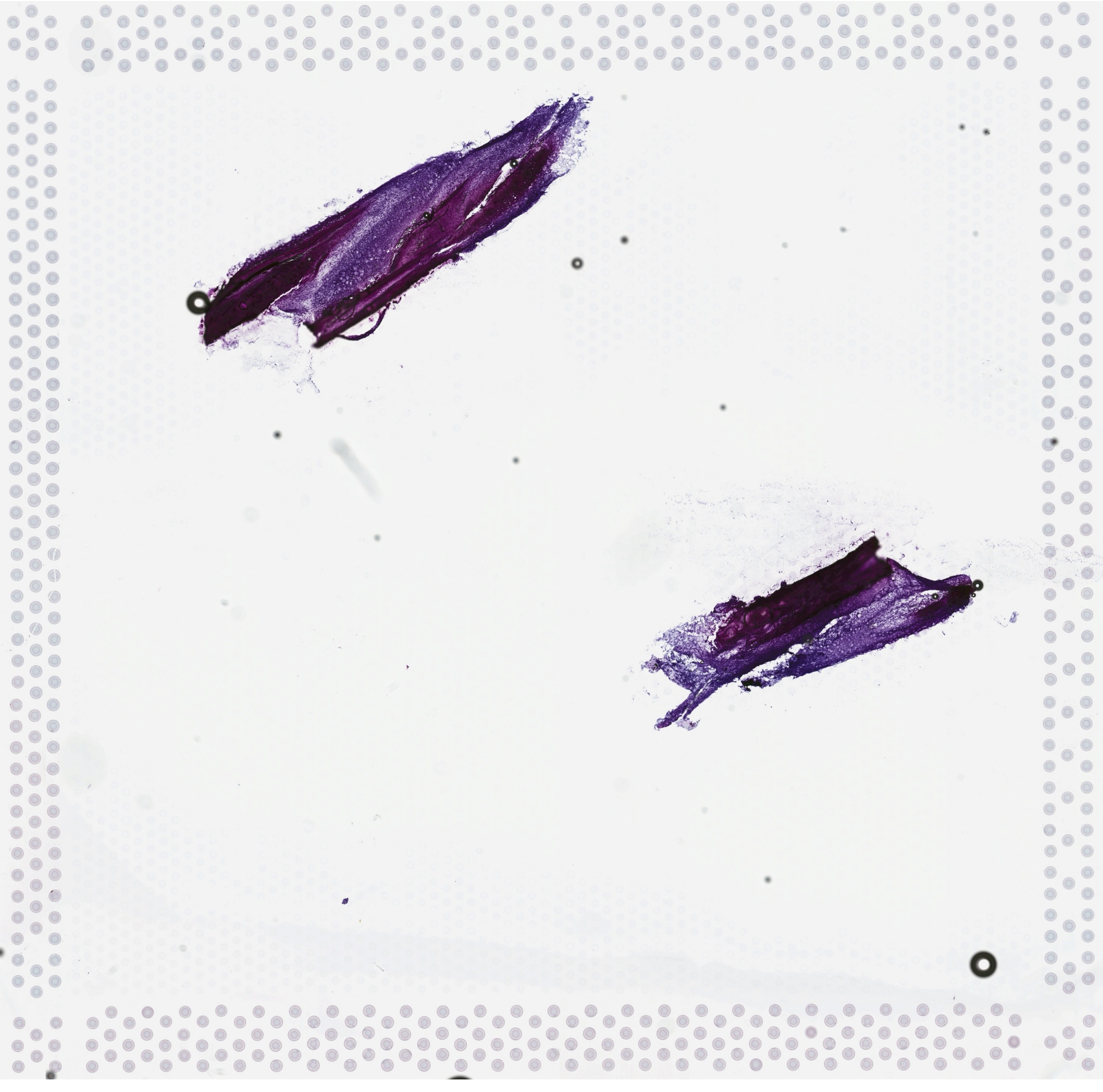

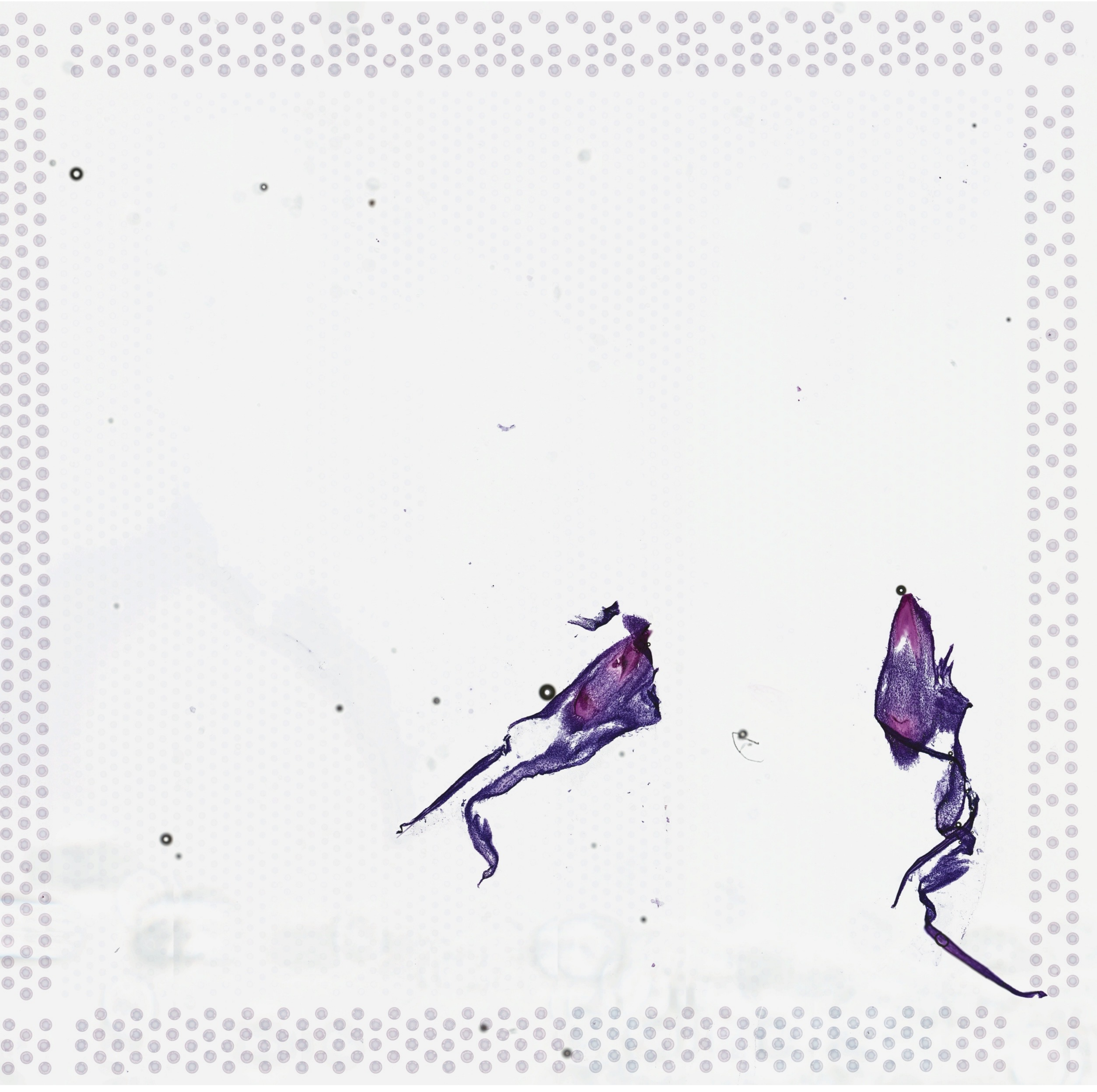

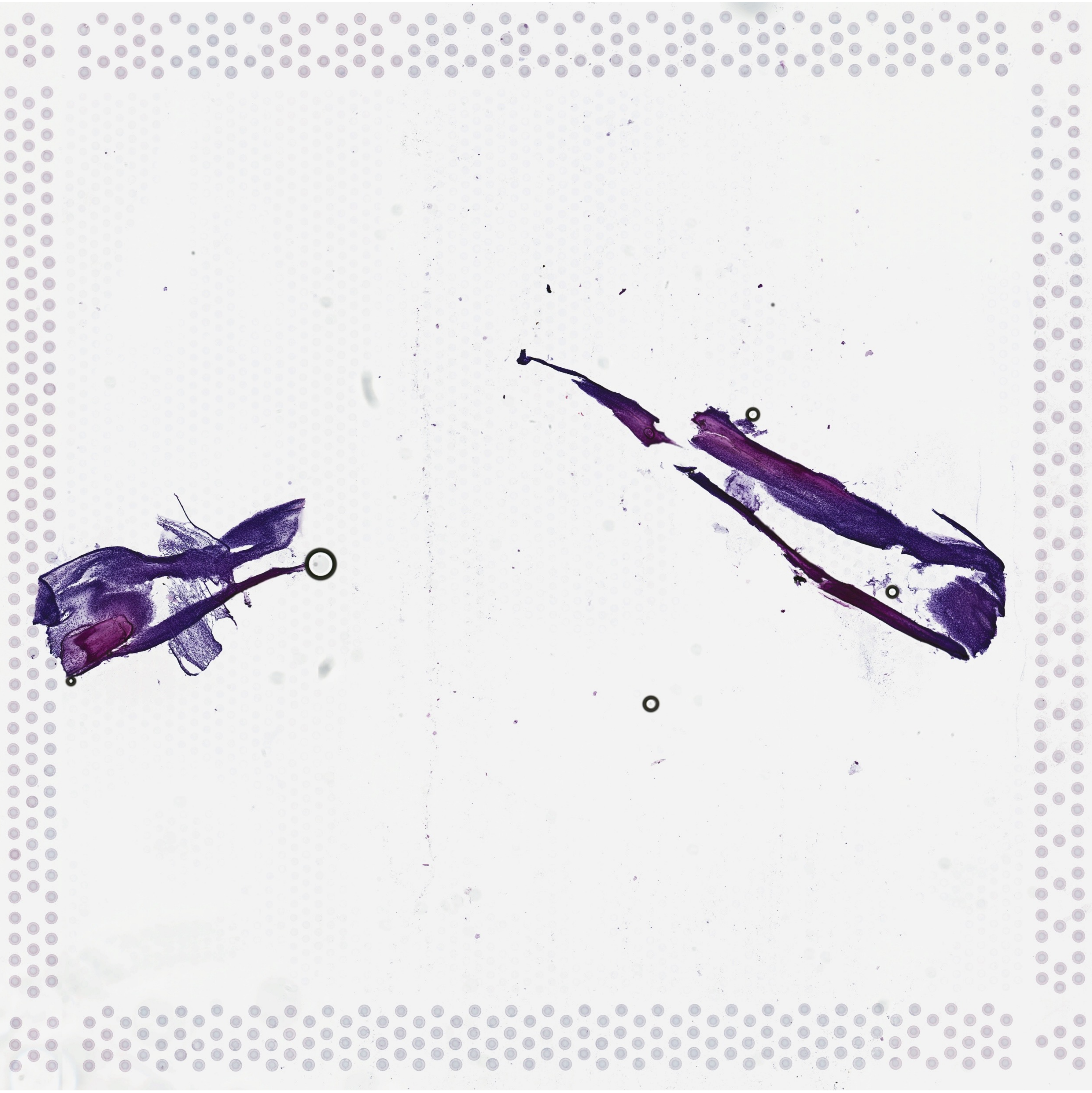

Supplement: Supplementary file 2 — Supplementary file2 (PDF 74818 KB) [file 441_2025_3976_MOESM2_ESM.pdf]
